# Chebulinic acid isolated from aqueous extracts of *Terminalia chebula* Retz inhibits *Helicobacter pylori* infection by potential binding to Cag A protein and regulating adhesion

**DOI:** 10.3389/fmicb.2024.1416794

**Published:** 2024-10-02

**Authors:** Ling Ou, Yajie Hao, Hengrui Liu, Zhixiang Zhu, Qingwei Li, Qingchang Chen, Ruixia Wei, Zhong Feng, Guimin Zhang, Meicun Yao

**Affiliations:** ^1^School of Pharmaceutical Sciences, Sun Yat-sen University, Shenzhen, China; ^2^International Pharmaceutical Engineering Lab of Shandong Province, Feixian, China; ^3^Cancer Institute, Jinan University, Guangzhou, China; ^4^Yinuo Biomedical Company, Tianjin, China; ^5^School of Medicine and Pharmacy, Ocean University of China, Qingdao, China; ^6^Department of Biomedical Engineering, College of Design and Engineering, National University of Singapore, Singapore, Singapore; ^7^State Key Laboratory of Integration and Innovation of Classic Formula and Modern Chinese Medicine, Lunan Pharmaceutical Group Co. Ltd., Linyi, China

**Keywords:** chebulinic acid, *Terminalia chebula*, *Helicobacter pylori*, adhesion, Cag A protein, minimum inhibitory concentration

## Abstract

**Background:**

*Terminalia chebula* Retz, known as the King of Tibet, is considered a functional food in China, celebrated for its antioxidant, immune-modulating, antibacterial, and anti-inflammatory properties. Chebulinic acid, derived from aqueous extracts of *Terminalia chebula* Retz, is known for its anti-inflammatory properties. However, its potential as an anti-*Helicobacter pylori* (HP) agent has not been fully explored.

**Methods:**

Herein, we extracted the main compound from *Terminalia chebula* Retz using a semi-preparative liquid chromatography (LC) system and identified compound 5 as chebulinic acid through Ultra-high performance liquid chromatography-MS/MS (UPLC–MS/MS) and Nuclear Magnetic Resonance (NMR). To evaluate its role, we conducted minimum inhibitory concentration (MIC) and minimum bactericidal concentration (MBC) assays, scanning electron microscope (SEM) imaging, inhibiting kinetics curves, urea fast test, cell counting kit-8 (CCK-8) assay, western blot analysis, griess reagent system, and molecular docking.

**Results:**

Our results showed that chebulinic acid effectively inhibited the growth of the HP strain ATCC 700392, damaged the HP structure, and exhibited selective antimicrobial activity without affecting normal epithelial cells GES-1. Importantly, it suppressed the expression of Cytotoxin-associated gene A (Cag A) protein, a crucial factor in HP infection. Molecular docking analysis predicted a strong affinity (−9.7 kcal/mol) between chebulinic acid and Cag A protein.

**Conclusion:**

Overall, our findings suggest that chebulinic acid acts as an anti-adhesive agent, disrupting the adhesion of HP to host cells, which is a critical step in HP infection. It also suppresses the Cag A protein. These results highlight the potential of chebulinic acid against HP infections.

## Graphical abstract

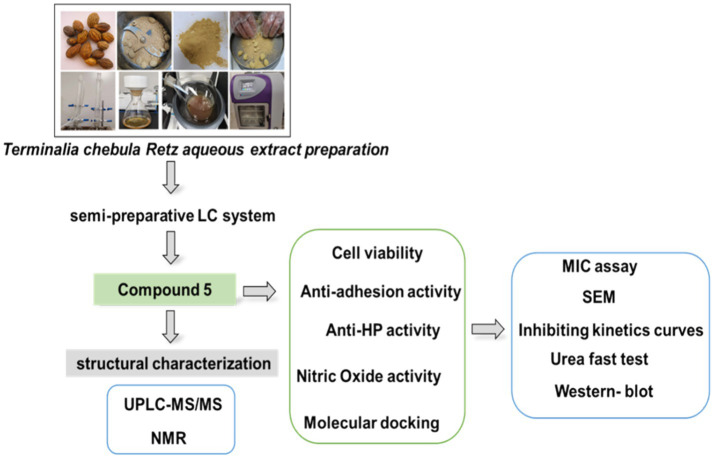

## 1 Introduction

*Helicobacter pylori* (HP) is a gram-negative bacterium that is frequently implicated in various gastrointestinal diseases (Burkitt et al., 2017), with peptic ulcers and gastritis being the most prevalent (Zheng et al., 2023; Moss and Calam, 1992; Hawkey et al., 1998; Miwa et al., 2004). Additionally, HP acts as a risk factor in gastric carcinogenesis (Lim and Chung, 2023; Ding et al., 2010; Bahnassy et al., 2018), one of the most prevalent cancer types (Ghorbani et al., 2024). It is estimated that around half of the world’s population is infected with HP. The presence of HP in the stomach triggers an immune response, leading to inflammation and the recruitment of immune cells to the site of infection (Yang et al., 2021). Over time, this chronic inflammation can result in the disruption of the gastric mucosal barrier. In addition, HP can produce toxins that directly damage the gastric epithelial cells, leading to gastritis (Lim and Chung, 2023; Sayed et al., 2020; Zhou et al., 2023; Zhao et al., 2023). This damage can progress through the transition from chronic atrophic gastritis to metaplastic changes, eventually, inducing gastric cancer (Bornschein and Malfertheiner, 2014).

Treatment of HP infection usually involves a combination of antibiotics and acid-suppressing medications to eradicate the bacterium and promote healing of the gastric mucosa (Malfertheiner et al., 2017; Ranjbar et al., 2017). Prevention of HP infection primarily focuses on improving sanitation and hygiene practices, as well as avoiding the consumption of contaminated food and water (Aziz et al., 2015; Baker and Hegarty, 2001). Additionally, strategies to reduce the transmission of HP within families and communities can also be effective in preventing new infections (Soares et al., 2023; Yu et al., 2022).

The efficacy of commonly used antimicrobial agents, including clarithromycin, levofloxacin, and metronidazole (Sousa et al., 2022), in treating HP infection has been compromised due to the increasing antibiotic resistance of HP (Dascalu et al., 2023; Smith and Yamaoka, 2023; Fauzia and Tuan, 2024). The emergence of antibiotic resistance has led to a significant failure in eradicating the infection. Therefore, it needs to search for natural products as alternative therapies. In recent years, there has been growing evidence supporting the efficacy of traditional Chinese medicines (TCMs) in the treatment of various diseases (Zhong et al., 2023; Liu et al., 2018). A review evaluating the effectiveness and safety of TCMs for HP treatment has suggested their potential as alternative therapies for HP infection (Zhong et al., 2022). In addition, studies proved that some natural products found in food possess anti-HP activity and serve as potential anti-HP agents, such as sulforaphane, curcumin, and hesperetin (Mohammadi et al., 2022; Haristoy et al., 2003; Kim et al., 2021).

Chebulinic acid, a natural compound abundant in several medicinal plants, particularly *Terminalia chebula*, known as *black myrobalan* or *Haritaki*, showcases a wide array of pharmacological properties and has garnered significant attention for its potential therapeutic advantages. Renowned for its potent antioxidant traits, chebulinic acid efficiently scavenges free radicals, safeguarding cells against oxidative harm (Biradar et al., 2019; Song et al., 2018). This attribute holds paramount importance in averting various chronic ailments and age-related degenerative processes. Moreover, chebulinic acid demonstrates anti-inflammatory properties by impeding pro-inflammatory mediators and signaling pathways, rendering it valuable in the management of conditions like arthritis (Lu et al., 2020), antiulcer activity (Negi et al., 2022), and inflammatory bone loss (Sharma et al., 2023). Furthermore, chebulinic acid demonstrates broad-spectrum antimicrobial activity against bacteria, fungi, and viruses (Thomas et al., 2022; Li et al., 2020), aligning with its traditional use in treating infections and promoting overall health. Research indicates potential anticancer properties of chebulinic acid through apoptosis induction and inhibition of cancer cell proliferation, positioning it as a promising candidate for cancer prevention and treatment (Aashima et al., 2024; Wang et al., 2018; Sharma et al., 2020). In addition, chebulinic acid exhibits hepatoprotective activity by mitigating liver damage caused by toxins, alcohol, or other harmful agents, offering benefits for liver disorders and overall liver health (Feng et al., 2021). Its multifaceted pharmacological actions encompass antioxidant, anti-inflammatory, antimicrobial, anticancer, gastrointestinal, and hepatoprotective properties, highlighting its potential therapeutic value across various health conditions.

In our recent study, we focused on investigating the anti-HP activity of specific TCMs, such as *Sanguisorba officinalis* L. (Shen et al., 2021), *Syzygium aromaticum* (Peng et al., 2022), *Canarium album* Raeusch (Yan et al., 2022), *Terminalia chebula* Retz (Ou et al., 2024). However, whether chebulinic acid as one of the main components of *Terminalia chebula* Retz is responsible for this activity has not been fully identified. In this study, we present our preliminary findings that highlight chebulinic acid as the potential active compound extracted from the aqueous extract of *Terminalia chebula* Retz, demonstrating the potential of chebulinic acid as a targeted Cag A agent, effectively inhibiting the activity of HP and functioning as an anti-adhesive agent against HP infections.

## 2 Materials and methods

### 2.1 Reagents

Columbia agar base and brain heart infusion (BHI) were acquired from Oxoid Ltd. Defibrinated sheep blood was obtained from Hongquan Biotechnology. Phosphate-buffered saline (PBS), Roswell Park Memorial Institute (RPMI) 1640 medium, and Fetal Bovine Serum (FBS) were supplied by Gibco-life Technologies LLC. Beyotime provided various products including RIPA reagent, PMSF, phosphatase inhibitor cocktail (50×), sodium dodecyl sulfate–polyacrylamide gel electrophoresis (SDS-PAGE) Gel Quick Preparation Kit, BeyoColorTM Prestained Color Protein Marker, BeyoECL Star Ultrasensitive Chemiluminescence Kit, and secondary antibodies against rabbit-HRP and mouse-HRP. Roche supplied the protease inhibitor cocktail, while Santa Cruz provided Anti-HP Cag A and m-IgG Fc BP-HRP.

### 2.2 *Terminalia chebula* Retz aqueous extract preparation

The mature fruit of *Terminalia chebula* Retz (Yunnan, China, Guangzhou Zhining Pharmaceutical Co. Ltd., Lot No. 210901) was authenticated as *T. chebula* by the Chief Pharmacist, Weixing Zhu. It underwent extraction and preparation as follows: a 20 g sample of the mature fruit was boiled three times at 90°C for 1 h with 10-fold double-distilled water. The resulting extract was concentrated by centrifugation, freeze-dried, and stored at −20°C, as previously described (Ou et al., 2024).

### 2.3 The preparation of compound 5 using a semi-preparative LC system

The preparation of compound 5 involved several steps using a semi-preparative LC system. Initially, a 5 g water extract of *Terminalia chebula* Retz was dissolved in 170 mL of water and filtered through a 0.22 μm filter. Subsequently, the sample underwent separation and preparation using a semi-preparative LC system with specific chromatographic conditions. A YMC Actus Triart C18 column was utilized with a gradient elution profile of 0 min: 95% A, 5% B; 50 min: 86% A, 14% B; 80 min: 76% A, 24% B; 80.10 min: 0% A, 100% B; 85 min: 0% A, 100% B; 85.1 min: 95% A, 5% B; 90 min: 95% A, 5% B. Compound 5, identified during the 76-min peak, was freeze-dried for structural characterization.

### 2.4 Ultra-high performance liquid chromatography-MS/MS

Compound 5 underwent liquid chromatography-mass spectrometry analysis utilizing a YMC Triart C18 column with specified gradient conditions. The analysis was performed under the following conditions: 0 min: 95% A, 5% B; 20 min: 70% A, 30% B; 27 min: 70% A, 30% B; 27.10 min: 95% A, 5% B; 40 min: 95% A, 5% B. The detector operated at 270 nm with a flow rate of 0.2 mL/min in Full MS ddMS2 mode. Spray voltage, auxiliary gas flow rate, auxiliary gas heater temperature, sheath gas flow rate, and capillary temperature were adjusted accordingly.

### 2.5 Nuclear magnetic resonance identification

Compound 5 was dissolved in Methanol-D4 for structural identification and analyzed using the AVANCE NEO 600 M system from Bruker BioSpin GmBH in Rheinstetten, Germany.

### 2.6 HP culture, cell culture, and co-culture

The standard HP strains, including ATCC 43504, ATCC 700392, and GES-1 cells, were acquired from the American Type Culture Collection (ATCC, Manassas, VA, United States). CS01 was generously provided by Professor Jing Liu from the University of Shanghai for Science and Technology, China, while QYZ-003 and QYZ-004 were obtained from Qingyuan Hospital of Traditional Chinese Medicine (Qingyuan, Guangdong, China). All HP strains underwent verification, culture, and storage at the School of Pharmaceutical Sciences (Shenzhen), Sun Yat-sen University. The strains were cultivated on Columbia agar base supplemented with 5% sterile defibrinated sheep blood or in BHI broth with 10% FBS. Cultures were agitated at 150 rpm and placed in a tri-gas incubator (ESCO, Singapore) at 37°C with a gas mixture of 10% CO_2_, 5% O_2_, and 85% N_2_ for a period of 2–3 days. GES-1 cells were cultured in RPMI 1640 medium with 10% FBS. For co-culture experiments, GES-1 cells were initially incubated overnight in 24-well plates. Subsequently, HP strains were added at a multiplicity of infection (MOI) ratio of 100:1, along with varying drug concentrations, and co-incubated for a period of 6 h before subsequent analyses.

### 2.7 Minimum inhibitory concentration assay and minimum bactericidal concentration assay

The MIC assays against HP were performed using the broth microdilution method in 96-well plates, as outlined by Shen et al. (2021). Following the determination of MIC values, 100 μL of the drug-treated solution at concentrations 1, 2, 4, and 8 times the MIC were spread onto Columbia agar base supplemented with 5% sheep blood. Subsequently, the minimum bactericidal concentration (MBC) was assessed after incubation for 5 days.

### 2.8 Scanning electron microscope

Subsequently, the bacterial cells were centrifuged at 6,000 rpm for 3 min, followed by two washes with phosphate-buffered saline. The samples were then fixed in a 2.5% glutaraldehyde solution and incubated overnight at 4°C. Initial dehydration was achieved using a stepwise ethanol series, succeeded by lyophilization and further fixation. The specimens were prepared for observation on a Scanning Electron Microscope (ZEISS Sigma 500, Germany) by subjecting them to metal coating, following the methodology detailed in a previous research study (Ou et al., 2024).

### 2.9 Inhibiting kinetics curves

Inhibitory kinetics curves were constructed by exposing HP ATCC 700392 to different drug concentrations. Samples were collected at specified time intervals (0, 12, 24, 36, 48, 60, and 72 h), and 100 μL aliquots were taken for absorbance measurements at OD 600 nm, in accordance with established protocols (Ou et al., 2024).

### 2.10 Nitric oxide activity

After a 6-h co-culture period, the supernatant from the cell culture was collected for assessing NO activity using the Griess Reagent System kit following the manufacturer’s instructions (Gotoh and Mori, 1999). Subsequently, the optical density at 540 nm was measured using a microplate reader (Multiskan GO, Thermo Scientific, United States).

### 2.11 Urea fast test

After treating the HP strain ATCC 700392 with drugs for 24 h, urea test solutions (7 mM phosphate buffer pH 6.8, 110 mM urea, 10 mg/L phenol red) (Rokka et al., 2008) were added. Following this, the optical density at 560 nm was determined using a microplate reader (Multiskan GO, Thermo Scientific, United States).

### 2.12 Cell viability

Cell viability was assessed using the Cell Counting Kit-8 (CCK-8) assay following the manufacturer’s protocol. Initially, 10,000 GES-1 cells were seeded in a 96-well plate and allowed to incubate overnight. Following this, the cells were treated with the drug for 24 h. After the treatment period, CCK-8 solutions were added to the plate and incubated for an additional 2 h. Subsequently, the optical density at 450 nm was measured using a microplate reader (Multiskan GO, Thermo Scientific, United States).

### 2.13 Cell adhesion assay

After a 6-h co-culture, the cells underwent two washes with PBS. The quantification of adherent bacteria was achieved by applying a urease assay, as previously established (Shmuely et al., 2004). Following this, urea test solutions were introduced, and the optical density at 560 nm was measured using a microplate reader (Multiskan GO, Thermo Scientific, United States).

### 2.14 Western blot

Western blot analysis was performed as in previous studies (Liu et al., 2018; Li et al., 2017). In this study, we applied it to the HP strain ATCC 700392 after 24 h of drug treatment. Protein extraction and lysis were carried out using RIPA buffer supplemented with PMSF and a protein inhibitor. Subsequently, protein concentrations were determined using the BCA method, and the lysates were heated with a loading buffer at 100°C for 10 min. Thirty micrograms of proteins from each sample were loaded onto SDS-PAGE gels, transferred to PVDF membranes, blocked with 5% milk, and then exposed to anti-Cag A as the primary antibody overnight at 4°C. After three washes with PBST for 10 min each, the membranes were incubated with the secondary antibody for 1 h at room temperature. Finally, membrane visualization was performed using BeyoECL (Beyotime, China) with a ChemiScope 6200 Visualizer (Clinx Science Instruments).

### 2.15 Molecular docking

Computational has been used in many previous studies (Morris and Lim-Wilby, 2008; Beaudoin et al., 2024). The AlphaFold2 (Bryant et al., 2022) algorithm was utilized to predict the structure of the Cag A protein (UniProt ID: L7VTT0) from HP. Additionally, the structure of Chebulinic acid was retrieved from the PubChem database. CB-Dock, specifically designed for blind docking within predicted protein binding sites, was employed to facilitate the binding interaction between Cag A protein and Chebulinic acid (CAS:18942-26-2). The initial step involved identifying potential binding sites through cavity detection. Subsequently, several top cavities, typically indicative of ligand binding sites, were selected based on their size for further analysis, a process known as cavity sorting. Following this, the docking center was determined, and the size of the docking box was adjusted accordingly. These parameters were crucial for conducting molecular docking utilizing AutoDock Vina (Trott and Olson, 2010) (Center and Size). Upon completion of the docking procedure, bound poses were re-evaluated based on docking scores (Dock and Rerank). The top-ranked conformation was considered the optimal binding pose, with the corresponding site identified as the optimal binding site for the queried ligand.

### 2.16 Statistical analysis

Statistical analysis was performed using GraphPad Prism 8 software. Either the two-sided Student’s *t*-test or one-way ANOVA followed by a suitable *post-hoc* test was employed, with statistical significance indicated by *p < 0.05 or **p < 0.01.

## 3 Results

### 3.1 Identification of compound 5

**Figure 1:**
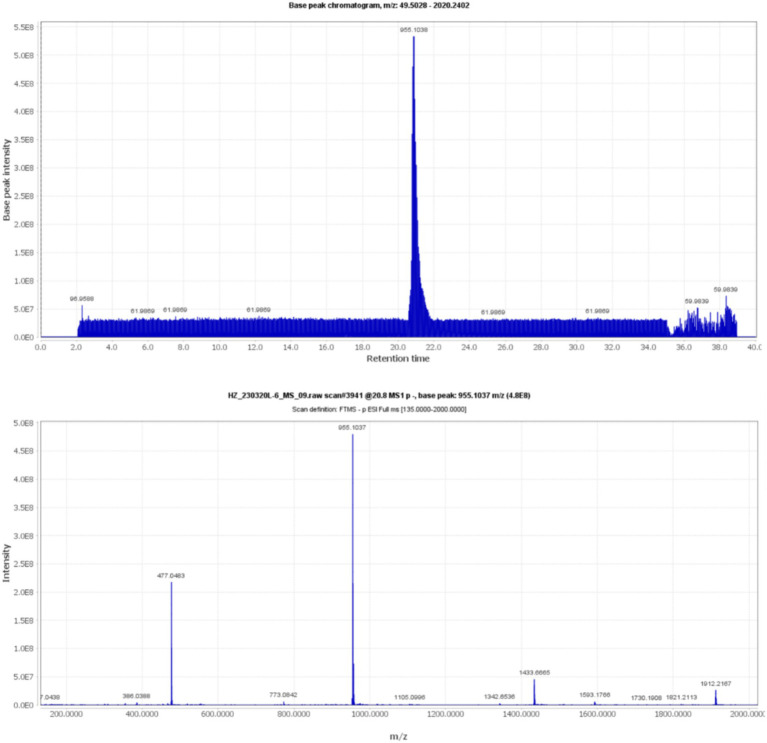
The UPLC-MS/MS data for compound 5 shows an m/z of 955.1038 in negative ion mode.

**Figure 2:**
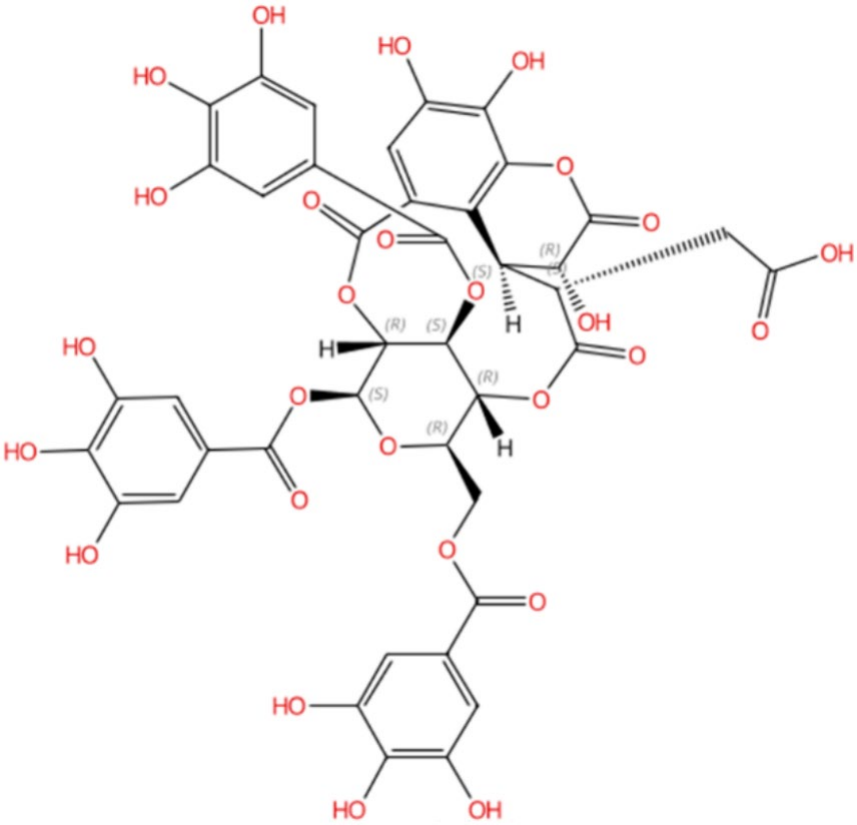
Compound 5 was identified as chebulinic acid through NMR analysis. The absolute stereochemistry was determined and visualized using the SciFinder website (https://scifinder-n.cas.org/search/all/65f851e04f64a43339f18374). The CAS number of Compound 5 is 18942-26-2.

Compound 5, detected with an m/z value of 955.1038 in negative ion mode (Figure 1), underwent additional structural elucidation via NMR analysis. Analysis of the data presented in Supplementary Figure S1, along with pertinent literature, led to the conclusive identification of compound 5 as chebulinic acid, with a CAS Registry Number of 18942-26-2, depicted in Figure 2.

### 3.2 Chebulinic acid demonstrated anti-HP activity characterized by bacteriostatic effects rather than bactericidal properties

To evaluate the influence of chebulinic acid on HP, MIC and MBC tests were conducted. The results presented in Table 1. revealed a range of MIC values for chebulinic acid, ranging from 16 to 64 μg/mL. MIC is an important measure in microbiology and antimicrobial testing because it indicates the potency of an antimicrobial agent against a particular microorganism. Lower MIC values indicate stronger antimicrobial activity. To further explore the killing effect of chebulinic acid on HP, we select representative strains for the MBC test, including a standard strain ATCC 700392 and a clinical strain QYZ004. The MBC of both strains tested were over 256 μg/mL, which exceeded 8 times the concentration of MIC. These findings suggest that chebulinic acid might exert a bacteriostatic effect on HP, impeding bacterial growth rather than inducing bacterial death.

**Table 1:** The minimum inhibitory concentration (MIC) and minimum bactericidal concentration (MBC) of chebulinic acid against *Helicobacter pylori* (HP) strains.

HP strains	MIC of Compound 5 (μg/mL)	MBC of Compound 5 (μg/mL)	MIC of clarithromycin (μg/mL)
ATCC 700392	32	>256	0.004
ATCC 43504	32	–	0.016
CS01	64	–	0.064
QYZ003	16	–	3.2
QYZ004	32	>256	>3.2

HP represents *Helicobacter pylori*; MIC represents minimum inhibitory concentration; MBC represents minimum bactericidal concentration; – represents not detect.

### 3.3 Chebulinic acid caused substantial harm to the structure of the bacteria

**Figure 3:**
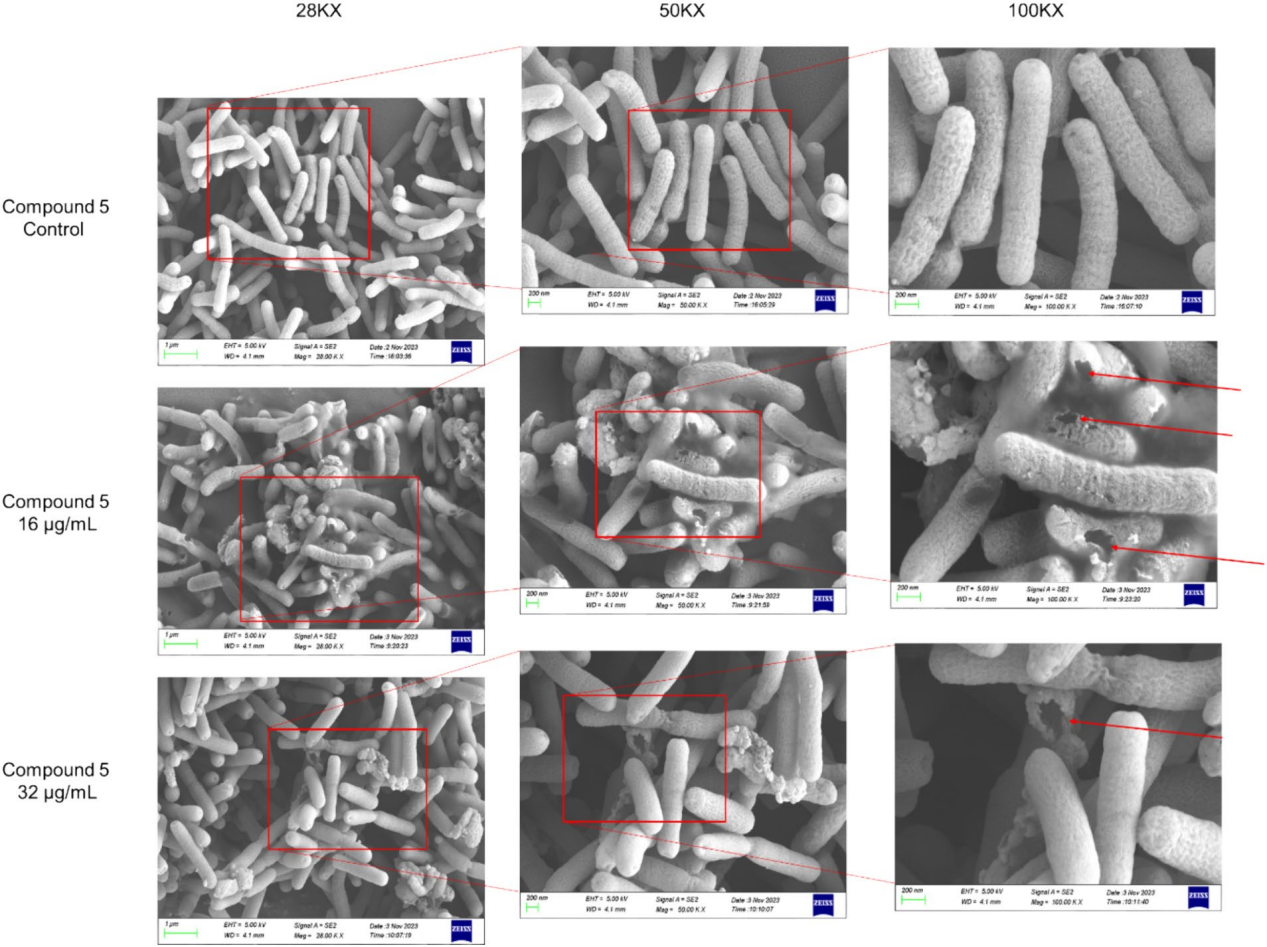
The SEM images depicting the structure of HP strain ATCC 700392 following a 24-h treatment with chebulinic acid.

Further investigation into the effects of chebulinic acid on the bacterial structure was conducted through SEM experiments. Figure 3 depicts that bacterial surfaces displayed damage after a 24-h exposure to chebulinic acid. The results suggest that chebulinic acid significantly disrupted the bacterial structure, leading to bacterial rupture.

### 3.4 Chebulinic acid suppressed the proliferation of HP strain ATCC 700392

**Figure 4:**
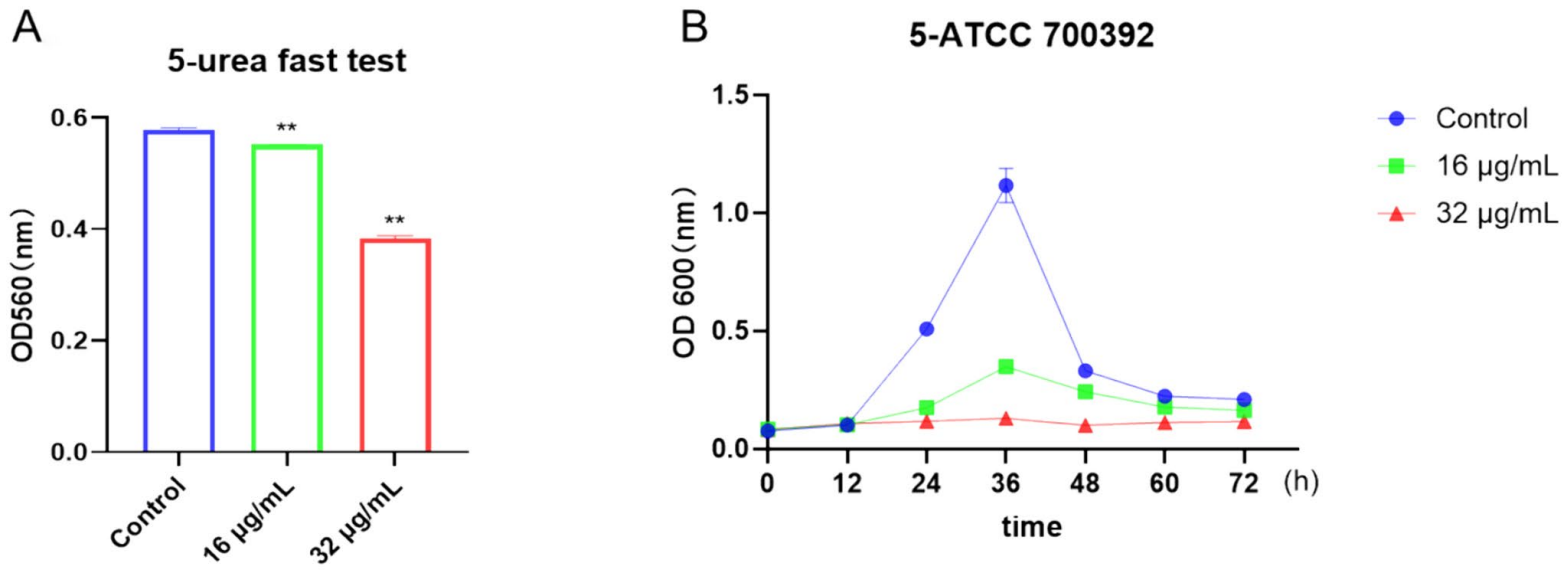
Chebulinic acid suppressed the growth of the HP strain ATCC 700392. **(A)** The rapid urease test. **(B)** The growth curve analysis of ATCC 700392. The statistical significance was indicated by **p < 0.01.

To visually evaluate the effects of drugs on bacteria, we utilized the rapid urease test and growth curve method. As depicted in Figure 4A, urease activity was notably inhibited after treatment with 16 μg/mL or 32 μg/mL of chebulinic acid. Moreover, growth curve results illustrated a significant suppression of bacterial growth at concentrations of 16 μg/mL or 32 μg/mL within the 0 to 72-h timeframe (Figure 4B). Collectively, these findings demonstrate that chebulinic acid effectively hindered the growth of the HP strain ATCC 700392. Further analysis unveiled a sustained impact of chebulinic acid on bacteria at these concentrations, resulting in a reduction in bacterial numbers. Additionally, the substantial inhibition of urease activity implies that chebulinic acid may disrupt crucial biochemical processes within the bacteria.

### 3.5 Chebulinic acid inhibited the expression of the Cag A protein

**Figure 5:**
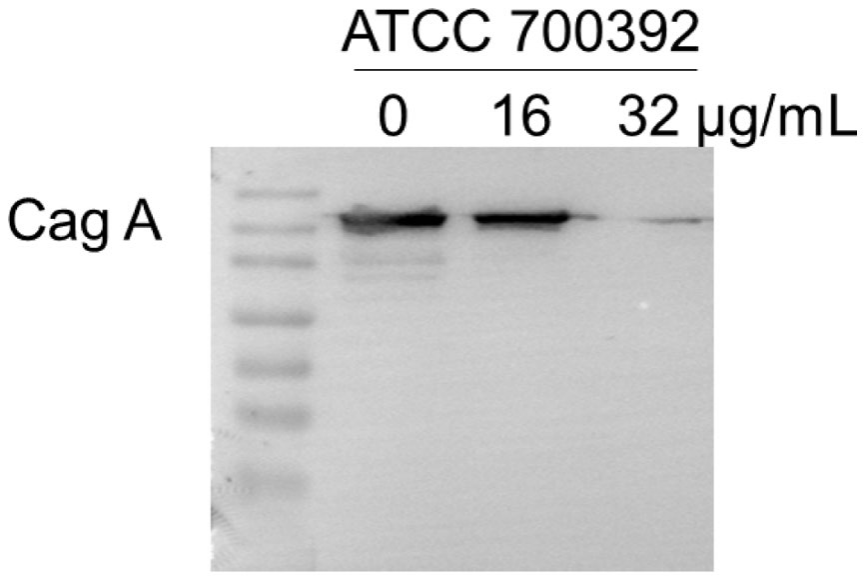
Chebulinic acid suppressed the expression of the Cag A protein. ATCC700392 were exposed to varying concentrations of chebulinic acid (0, 16, 32 μg/mL) for 24 h.

The Cag A protein, a critical element in HP infection, plays a central role in shaping the pathogenesis of the disease. To explore the potential impact of chebulinic acid on Cag A expression, we employed western blot analysis as a reliable method for detecting and quantifying proteins. Our study findings unequivocally revealed that the presence of 32 μg/mL of chebulinic acid significantly inhibited the expression of the virulence factor Cag A. This significant discovery not only sheds light on the therapeutic potential of chebulinic acid in combating HP-related infections but also emphasizes the necessity for further investigations to elucidate the underlying molecular mechanisms. The visual representation of our results in Figure 5 vividly illustrates the substantial influence of chebulinic acid on Cag A expression, underscoring the potential therapeutic value of this natural compound.

### 3.6 Chebulinic acid exhibited no significant cytotoxic effects on normal epithelial cells GES-1

**Figure 6:**
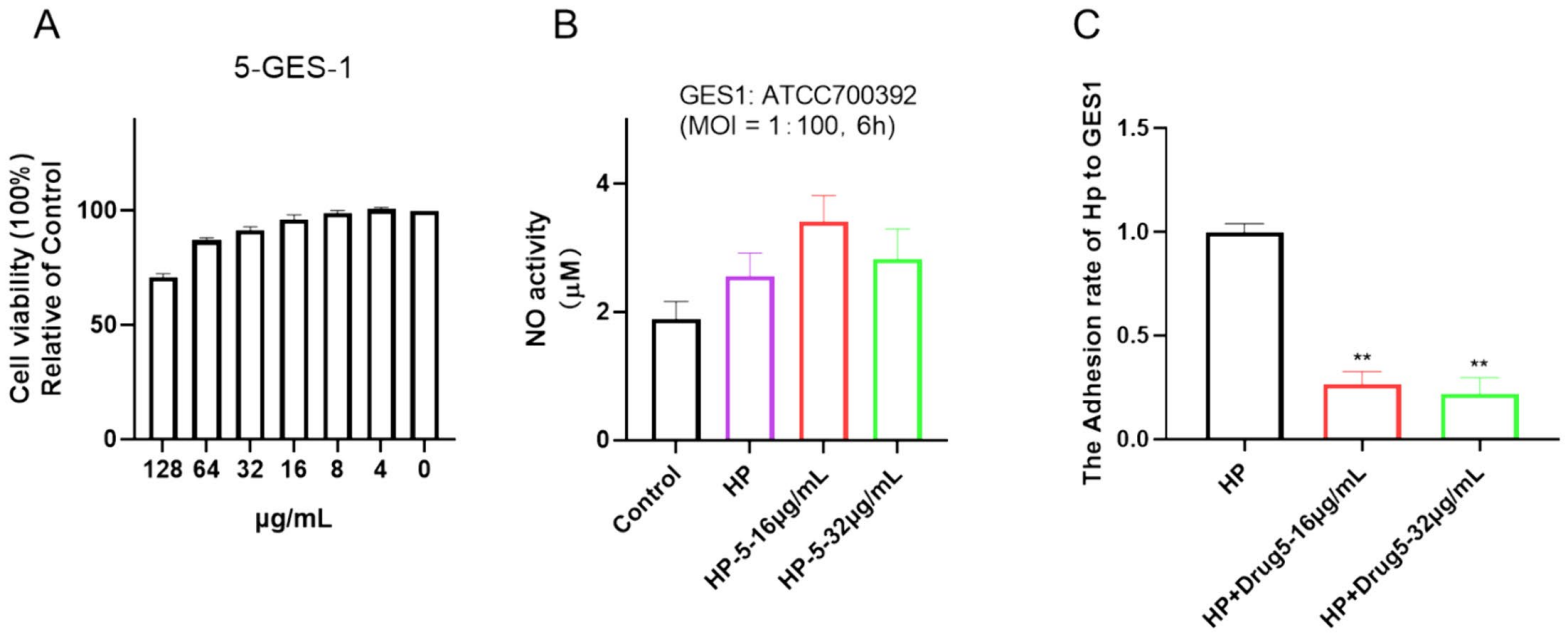
Chebulinic acid demonstrates anti-adhesive properties *in vitro*. **(A)** Cell viability. GES-1 cells were exposed to varying concentrations of chebulinic acid (0–128 μg/mL) for 24 h. **(B)** NO activity. **(C)** Anti-adhesive efficacy. GES-1 cells were co-cultured with *Helicobacter pylori* at a 1:100 ratio and treated with different concentrations of chebulinic acid (0, 16, 32 μg/mL) for 6 h. The statistical significance was indicated by **p < 0.01.

Cell viability assays were applied in many previous studies to determine cytotoxic effects (Li et al., 2018; Liu et al., 2020; Liu et al., 2021). In this study, the cytotoxicity of chebulinic acid was assessed using a CCK-8 assay. As depicted in Figure 6A, exposure to 64 μg/mL of chebulinic acid did not significantly affect the viability of normal epithelial cells GES-1, indicating its safety profile in this regard despite its antibacterial properties. To further explore its potential impacts, cells were co-cultured with bacteria and treated with chebulinic acid for 6 h, we investigated its anti-NO effects and observed no impact on NO activity (Figure 6B). Additionally, our findings revealed that chebulinic acid at concentrations of 16 or 32 μg/mL notably suppressed bacterial adhesion to the cells, suggesting its potential as an antibacterial adhesion inhibitor (Figure 6C).

### 3.7 Binding between Cag A in HP and chebulinic acid by molecular docking

**Figure 7:**
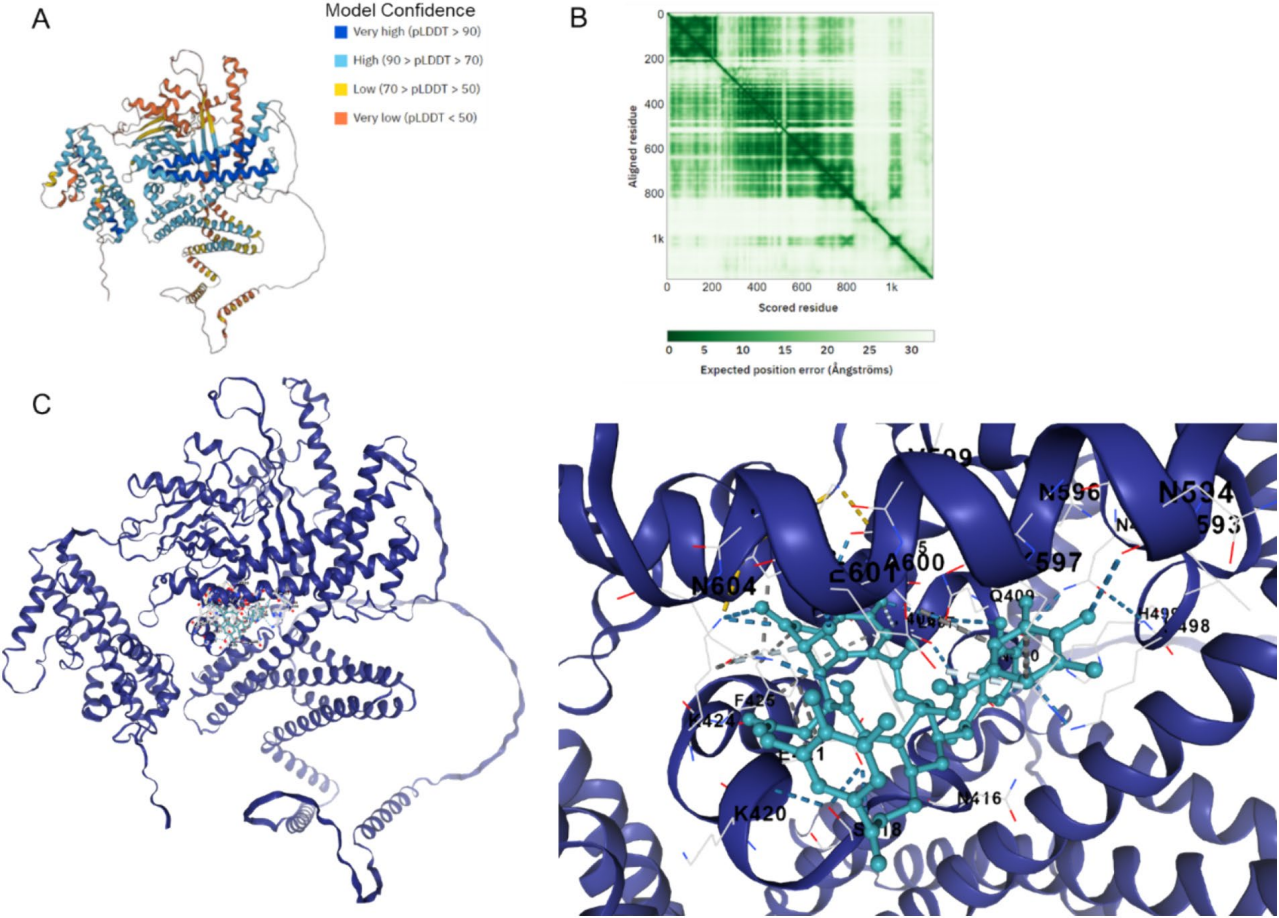
Binding between Cag A in HP and chebulinic acid. **(A)** Cag A protein structure calculated by the AlphaFold2 with model confidence. AlphaFold produces a per-residue model confidence score between 0 and 100. Some regions below 50 pLDDT may be unstructured in isolation. **(B)** Predicted aligned error. This data is useful for assessing inter-domain prediction accuracy. **(C)** Docking Binding between Cag A in HP and Chebulinic acid. The docking affinity is −9.7 Kal/mol.

Figure 7 illustrates the AlphaFold2-calculated structure of the Cag A protein, displaying model confidence scores for each residue, with regions scoring below 50 pLDDT potentially adopting unstructured states when considered individually (Figure 7A). Additionally, predicted alignment error data was utilized to depict the accuracy of predicted structural domains within the model (Figure 7B). Furthermore, the docking binding affinity between Cag A and chebulinic acid in HP demonstrates a robust binding interaction with a calculated affinity of −9.7 kcal/mol, indicating a high degree of binding affinity between these molecules (Figure 7C). Collectively, these results suggest that molecular docking predicted the binding between Cag A in HP and chebulinic acid.

## 4 Discussion

Marshall’s groundbreaking research provided the initial evidence that HP colonizes the stomach and can survive and thrive in its acidic environment. Infection by HP is typically acquired during childhood or within families, often through oral-oral or fecal-oral routes. Adhesion is the first step for persistent colonization after directional motility and HP secretes several virulence factors or outer membrane proteins to promote adhesion to gastric mucosa, afterward adhesion to the gastric mucosa, HP finally colonizes in the gastric mucosa, thus enabling it to establish chronic infection and cause damage to the gastric mucosa (Yang et al., 2023). The cag pathogenicity island plays a vital role in the colonization of the human stomach (Cover et al., 2020). Cag A protein is a critical virulence factor and a key part of the cag pathogenicity island, which is injected into host cells and has been linked to the development of gastric cancer (Tran et al., 2024; Graham and Yamaoka, 1998; Chung et al., 2019; Canzian et al., 2020).

Traditional medicine has been widely used for human disease treatment (Liu et al., 2018; Chen et al., 2016). Our current investigation has unveiled the anti-HP activity of chebulinic acid sourced from aqueous extracts of *Terminalia chebula* Retz. Electron microscopy analysis has revealed substantial damage to the structure of HP bacteria, resulting in structural impairment and fragmentation. Results showed that the treatment using 16 μg/mL chebulinic acid damages the structure of HP more severely than that with 32 μg/mL chebulinic acid. As for this observation, we postulate that at elevated concentrations, the drug may disrupt the delicate balance of intracellular ion concentrations and pH, subsequently influencing the drug’s efficacy and the functionality of various intracellular biomolecules. Moreover, these high concentrations can significantly alter the cell membrane’s fluidity, leading to a more compact or rigid structure. This alteration in membrane integrity can consequently impair the permeability, thereby limiting the entry and passage of drug molecules through the cellular barrier. Cells are likely to possess feedback regulatory mechanisms that, upon the excessive entry of drugs, prompt the cell to modulate transport proteins on the cell membrane or other relevant molecules to mitigate drug uptake. This adaptive response serves to safeguard the cell from excessive stimulation or damage.

The current findings suggest that chebulinic acid is more likely to act as a bacteriostatic agent instead of a bactericide since the MBC/MIC ratio is greater than 8. Generally, when the MBC to MIC ratio is more than 8, it implies that the antibacterial agent mainly functions by inhibiting bacterial growth instead of effectively killing the bacteria. This means that while chebulinic acid can suppress bacterial proliferation at lower concentrations, higher concentrations are required to eliminate the bacteria. While chebulinic acid may not function as a bactericidal agent, it exhibits promise as an antibacterial agent, particularly in impeding bacterial adhesion. Crucially, chebulinic acid has exhibited a safe profile by not inducing toxic side effects on normal cells at the concentration necessary for its antibacterial efficacy. In this study, we conducted both MIC and MBC testing, MIC is a crucial measure in microbiology and antimicrobial testing as it indicates the potency of an antimicrobial agent against a specific microorganism, with lower values denoting stronger activity. In contrast, MBC assesses whether the agent not only inhibits but also kills the microorganism. For our purposes, we focus on reporting bacterial inhibition rather than necessitating bacterial death. Therefore, MIC holds greater importance than MBC, prompting us to conduct MIC testing on all five strains of HP and select two representative strains—a standard and a clinical strain—for MBC tests. MIC testing demonstrates drug inhibition, a critical aspect, while MBC testing explores potential mechanisms behind this inhibition. Particularly when we observe that MIC alterations likely stem from bacteriostatic effects rather than bacterial death (both strains tested had MBC values exceeding 256 μg/mL, over eight times their MIC concentrations), further MBC testing may be unwarranted.

What’s more, chebulinic acid exhibited notably suppressed HP adhesion to the cells. Chebulinic acid boasts a distinctive chemical structure and biological activity that enables it to specifically bind to particular receptors on the surface of host cells. This characteristic endows it with the ability to modulate bacterial adhesion across multiple levels. Previous research has demonstrated that chebulinic acid inhibits the binding of HIV-1 gp120 to CD4, thereby exhibiting anti-HIV activity (Weaver et al., 1992). More recently, chebulinic acid has been identified as a novel potential antifungal agent against *Candida glabrata*, acting by binding to the CgMed15a KIX domain (Waseem et al., 2023). Additionally, chebulinic acid has shown significant efficacy in treating gastric ulcers due to its anti-secretory, anti-oxidative, and cytoprotective properties, as well as its ability to inhibit H(+) K(+)-ATPase activity (Mishra et al., 2013). We hypothesize that chebulinic acid may effectively inhibit bacterial adhesion to host cells through a combination of mechanisms, including competitive binding to bacterial receptors, alteration of the bacterial cell surface structure, disruption of bacterial signaling pathways, enhancement of the host’s immune response, physical exclusion, and modulation of bacterial metabolism.

Compared to the standard drug clarithromycin, chebulinic acid has a higher MIC level. The extensive and widespread use of clarithromycin has led to a concerning rise in its resistance rates. In light of this escalating issue, the introduction of novel therapeutic agents holds particular value, offering a much-needed alternative for patients who have developed resistance to clarithromycin, thereby presenting a critical opportunity to combat antibiotic resistance and preserve the efficacy of our treatment options. Although the MIC value for chebulinic acid is relatively high, this does not diminish its remarkable effectiveness in significantly inhibiting pathogen adherence. This characteristic positions it as a crucial candidate for combating infections, demonstrating a noteworthy therapeutic potential even at higher MIC levels.

Interestingly, experimental findings have demonstrated that chebulinic acid suppresses the expression of Cag A. Molecular docking analysis was employed to explore the interaction between chebulinic acid and Cag A, revealing a predicted binding affinity of −9.7 kcal/mol, suggesting that chebulinic acid may target Cag A. We speculate that it might combine with the virulence factors and thereby reduce their expression. Cag A’s role in HP’s adhesion to gastric epithelial cells is multifaceted. On one hand, Cag A may modulate the expression or activity of bacterial adhesins, directly impacting the adherence process between the bacteria and the cells. On the other hand, upon entering gastric epithelial cells, Cag A triggers a cascade of intracellular signaling pathways, leading to alterations in cellular morphology and function. These changes might indirectly affect the stability of HP’s adherence to the cell surface. Moreover, Cag A-induced inflammatory responses and cytoskeletal rearrangements within the cells could create a more favorable environment for the bacteria’s adhesion.

These findings not only underscore the potential of chebulinic acid in addressing HP infections but also open avenues for investigating its effectiveness against various bacterial strains. Furthermore, its favorable safety profile adds to its allure as a potential therapeutic agent. Crucially, chebulinic acid’s potential repressing of Cag A could play a pivotal role in combating HP infection and inhibiting HP adhesion to host cells, warranting continued research into its anti-adhesive and anti-virulence properties *in vivo*. These findings offer insights into the mechanism of action of chebulinic acid on bacteria, offering valuable clues for further exploration of its potential applications in the treatment of infectious diseases.

The main limitation of this study lies in the fact that we selected a single strain for the initial investigation of the preliminary mechanism exploration. We strive to develop the most effective model to illustrate drug effects, yet the culture environment differs from HP growth within the body. This experiment is constrained by variations in strain growth. In our preliminary research, HP strain ATCC 700392 exhibited notably superior growth compared to others, significantly impacting drug treatment outcomes. Consequently, this strain is ideal for exploring mechanisms. While acknowledging its limitation as a single strain, insufficient to ensure experiment reproducibility across others, it suits our goal of mechanism exploration. We address this potential limitation in our discussion. As the research progresses and resources accumulate, future studies will gradually expand the scope by employing multiple strains to conduct more comprehensive and in-depth investigations. In the subsequent steps, we will be able to further verify the binding of the drug to the Cag A target, providing a scientific basis for the development and application of the drug. In addition, the direct correlation between Cag A and adhesion needs to be further substantiated through additional research.

It is noteworthy that the discrepancy between *in vitro* culture conditions and the *in vivo* environment can affect HP’s growth characteristics and responsiveness to drugs or treatments. Laboratory cultures typically utilize artificially formulated media, which may not fully replicate the complex and physiological conditions of the human stomach. Factors such as pH levels, oxygen tension, nutrient availability, and the microbial community within the stomach differ from those *in vivo* conditions. To enhance the credibility of our research findings, we plan to conduct *in vivo* experiments in the future. These will help to validate the consistency between *in vitro* culture results and the actual *in vivo* scenarios. Further experimentation and research will improve the reliability and applicability of findings, contributing valuable information to the study and treatment of HP.

## 5 Conclusion

In summary, chebulinic acid has exhibited potent anti-HP activity by effectively inhibiting the growth of the HP strain ATCC 700392, suppressing the Cag A protein, and disrupting HP adhesion to host cells, a critical step in HP infection. Additionally, it has shown no impact on GES-1 cells. These findings underscore the potential of chebulinic acid as a therapeutic option against HP infections. Future research endeavors should prioritize understanding its anti-adhesive and anti-virulence properties, alongside evaluating its efficacy in relevant *in vivo* models. Clinical studies are imperative for assessing its safety and efficacy in humans, thereby advancing its potential application in addressing HP-related conditions.

## Data Availability

The original contributions presented in the study are included in the article/Supplementary material, further inquiries can be directed to the corresponding authors.
