# Supplementary material for: Chebulinic acid isolated from aqueous extracts of Terminalia chebula Retz inhibits Helicobacter pylori infection by potential binding to Cag A protein and regulating adhesion
Source: Front Microbiol. 2024 Oct 2;15:1416794. doi: 10.3389/fmicb.2024.1416794 (PMC11483367; doi:10.3389/fmicb.2024.1416794)
Supplement: Supplementary file 1 [file Data_Sheet_1.PDF]

HP001

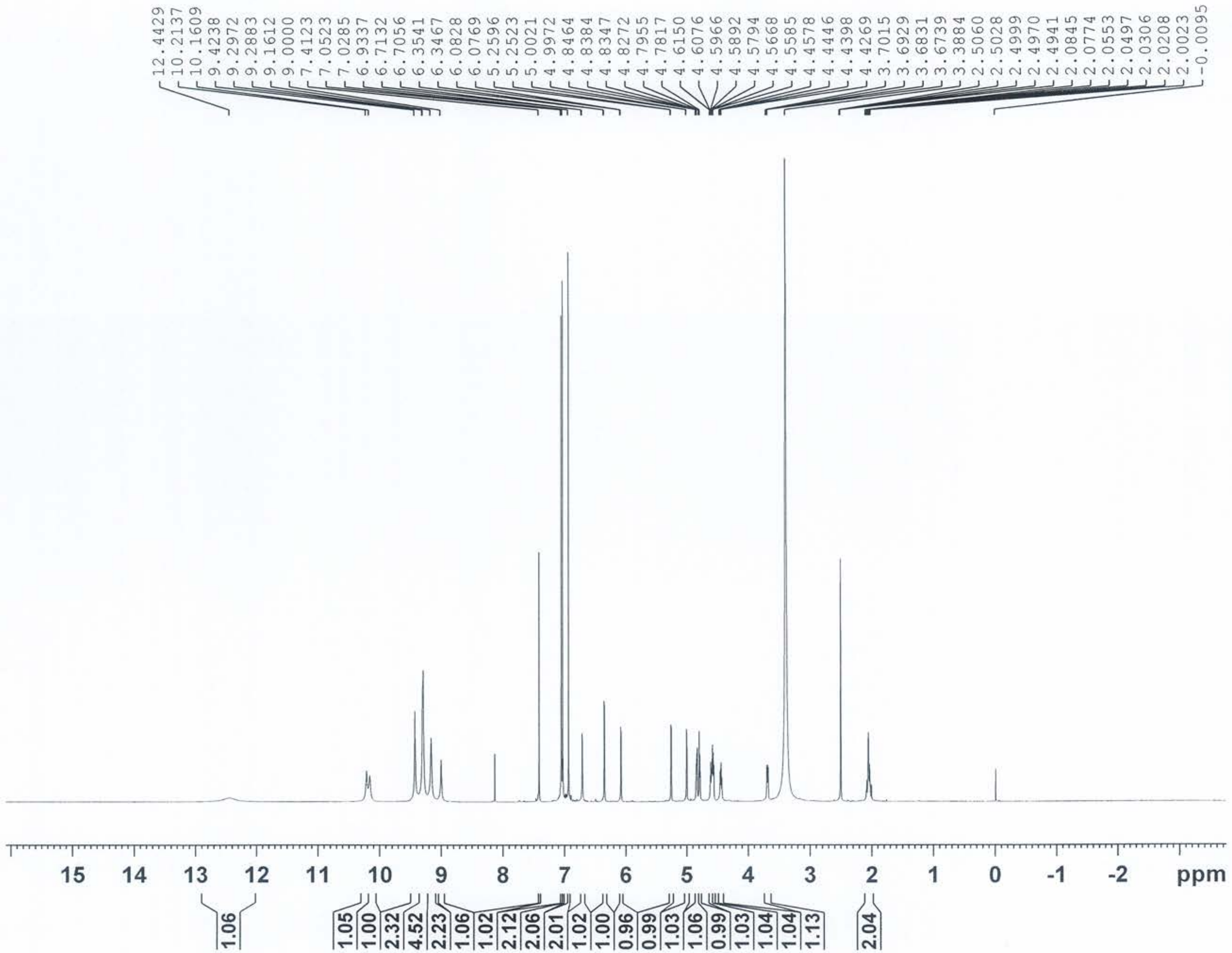

BRUKER

Current Data Parameters
NAME Component 5 of Chebulae Fructus_230401
EXPNO 1
PROCNO 1

F2 - Acquisition Parameters

Date_	20230930
Time_	16.33 h
INSTRUM	Avance
PROBHD	Z172446_0005 (
PULPROG	zg30
TD	65536
SOLVENT	DMSO
NS	16
DS	2
SWH	11904.762 Hz
FIDRES	0.363304 Hz
AQ	2.7525120 sec
RG	77.3036
DW	42.000 usec
DE	8.47 usec
TE	298.1 K
D1	1.00000000 sec
TD0	1
SFO1	600.1337058 MHz
NUC1	1H
P0	3.97 usec
P1	11.92 usec
PLW1	17.17900085 W

F2 - Processing parameters

SI	65536
SF	600.1300062 MHz
WDW	EM
SSB	0
LB	0.30 Hz
GB	0
PC	1.00

HP001

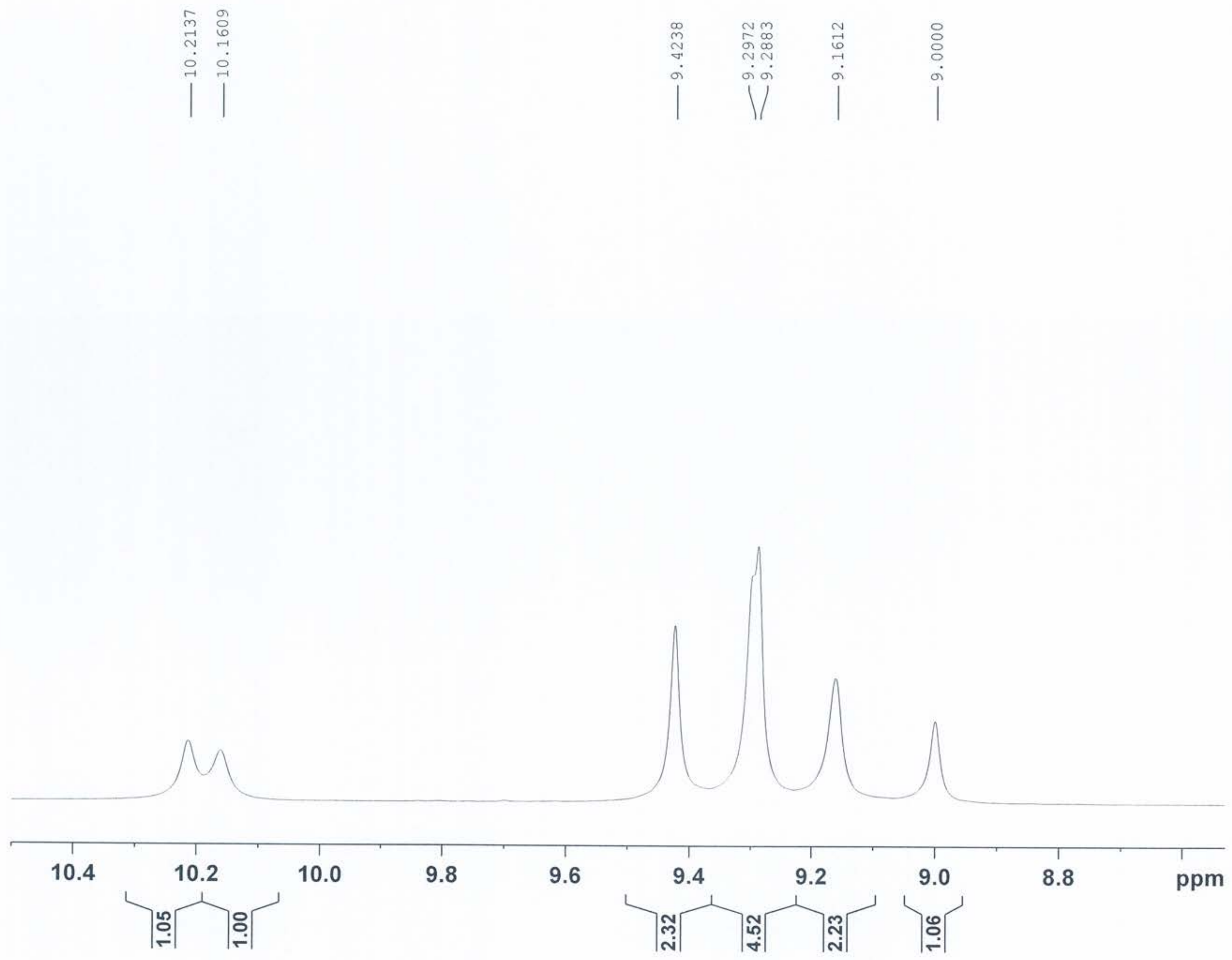

BRUKER

Current Data Parameters
NAME Component 5 of Chebulae Fructus_230401
EXPNO 1
PROCNO 1

F2 - Acquisition Parameters
Date_ 20230930
Time_ 16.33 h
INSTRUM Avance
PROBHD Z172446_0005 (
PULPROG zg30
TD 65536
SOLVENT DMSO
NS 16
DS 2
SWH 11904.762 Hz
FIDRES 0.363304 Hz
AQ 2.7525120 sec
RG 77.3036
DW 42.000 usec
DE 8.47 usec
TE 298.1 K
D1 1.00000000 sec
TD0 1
SFO1 600.1337058 MHz
NUC1 1H
P0 3.97 usec
P1 11.92 usec
PLW1 17.17900085 W

F2 - Processing parameters
SI 65536
SF 600.1300062 MHz
WDW EM
SSB 0
LB 0.30 Hz
GB 0
PC 1.00

[illegible] 23. 12.27
[illegible]

HP001

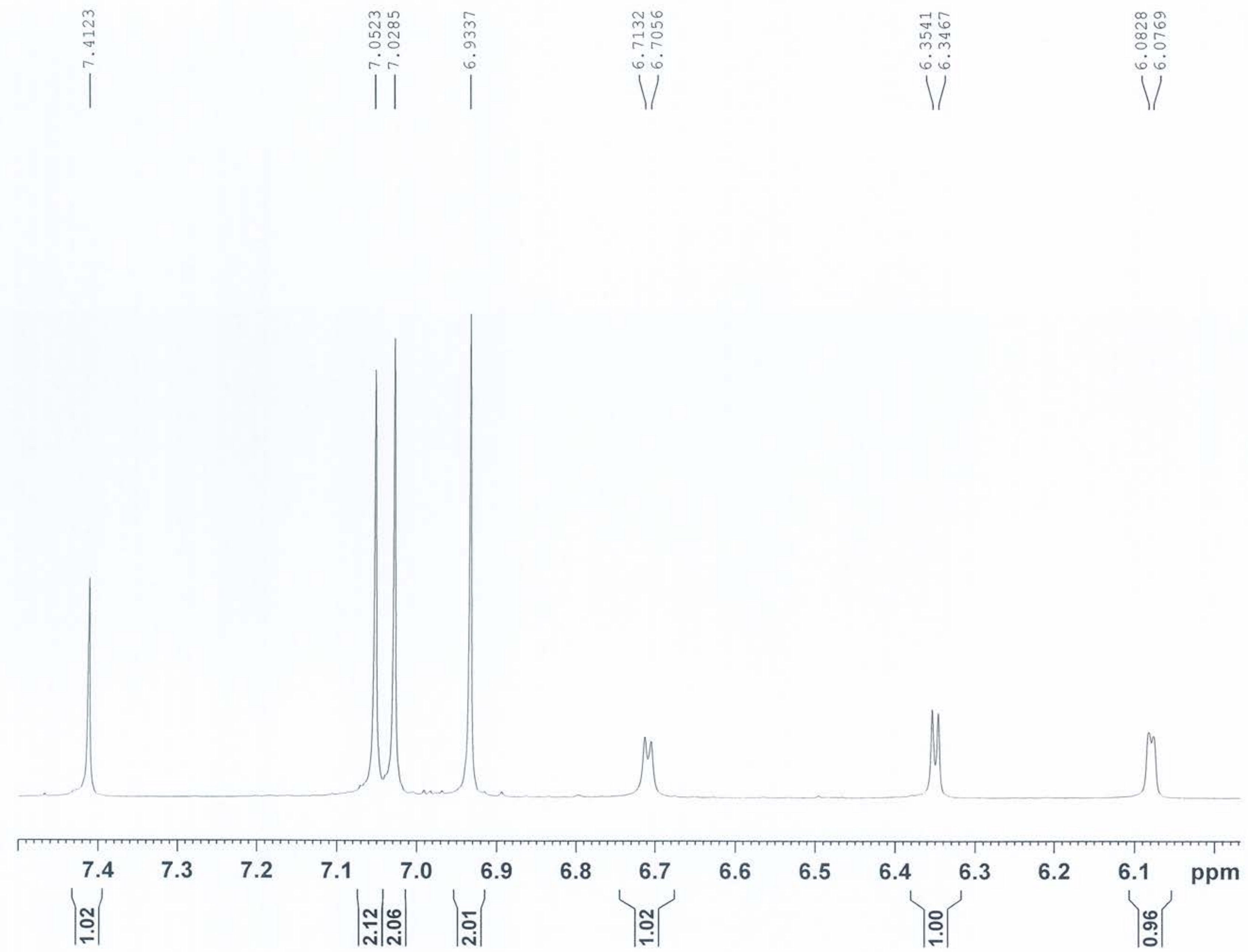

BRUKER

Current Data Parameters

NAME	Component 5 of Chebulae Fructus_230401
EXPNO	1
PROCNO	1

F2 - Acquisition Parameters

Date_	20230930
Time	16.33 h
INSTRUM	Avance
PROBHD	Z172446_0005 (
PULPROG	zg30
TD	65536
SOLVENT	DMSO
NS	16
DS	2
SWH	11904.762 Hz
FIDRES	0.363304 Hz
AQ	2.7525120 sec
RG	77.3036
DW	42.000 usec
DE	8.47 usec
TE	298.1 K
D1	1.00000000 sec
TD0	1
SFO1	600.1337058 MHz
NUC1	1H
P0	3.97 usec
P1	11.92 usec
PLW1	17.17900085 W

F2 - Processing parameters

SI	65536
SF	600.1300062 MHz
WDW	EM
SSB	0
LB	0.30 Hz
GB	0
PC	1.00

检测人：[illegible] 23.12.07
复核人：[illegible] 23.12.07

HP001

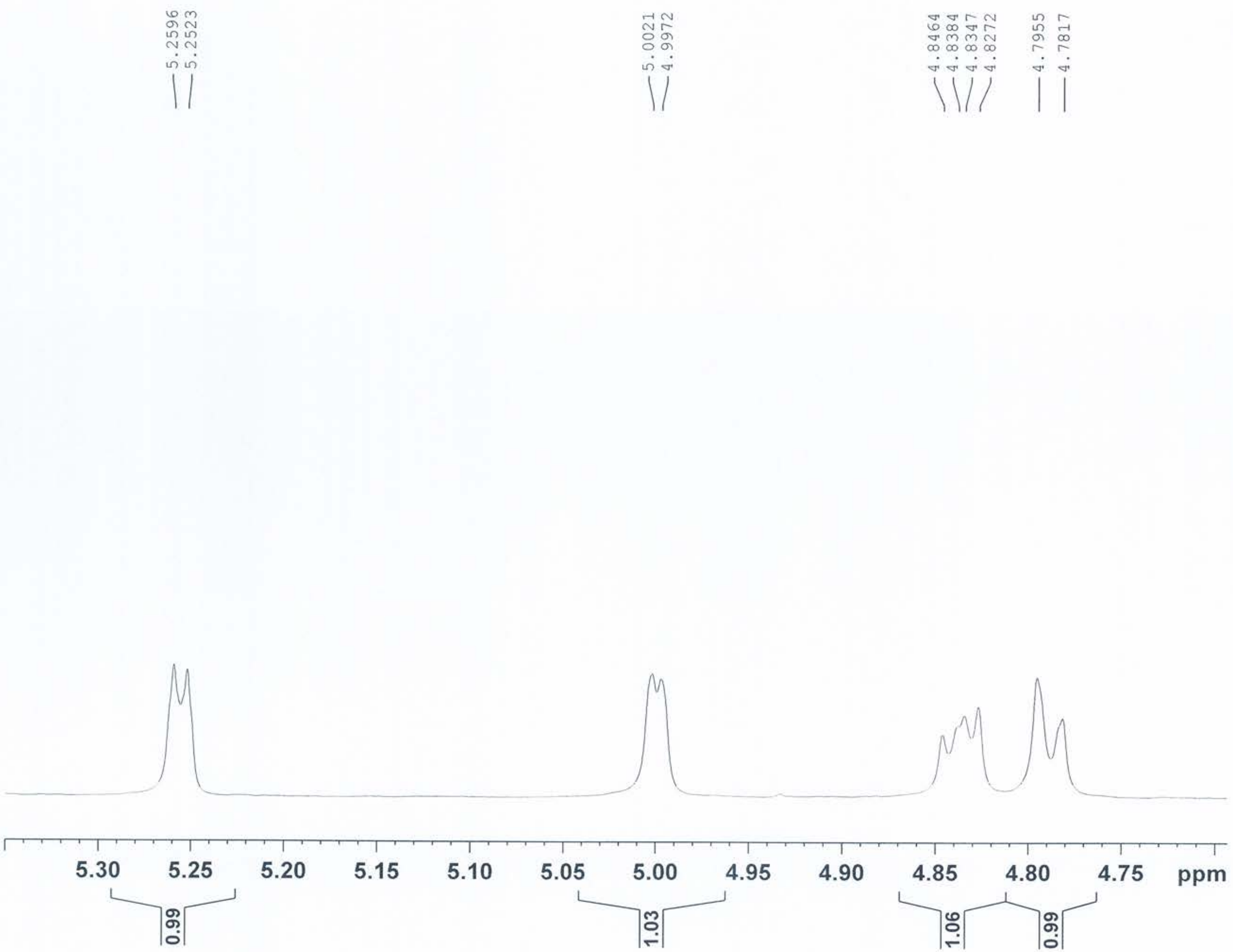

Current Data Parameters
NAME Component 5 of Chebulae Fructus_230401
EXPNO 1
PROCNO 1

F2 - Acquisition Parameters
Date_ 20230930
Time_ 16.33 h
INSTRUM Avance
PROBHD Z172446_0005 (
PULPROG zg30
TD 65536
SOLVENT DMSO
NS 16
DS 2
SWH 11904.762 Hz
FIDRES 0.363304 Hz
AQ 2.7525120 sec
RG 77.3036
DW 42.000 usec
DE 8.47 usec
TE 298.1 K
D1 1.00000000 sec
TD0 1
SFO1 600.1337058 MHz
NUC1 1H
P0 3.97 usec
P1 11.92 usec
PLW1 17.17900085 W

F2 - Processing parameters
SI 65536
SF 600.1300062 MHz
WDW EM
SSB 0
LB 0.30 Hz
GB 0
PC 1.00

检验人: [illegible] 23.12.07
复核人: [illegible] 23.12.07

HP001

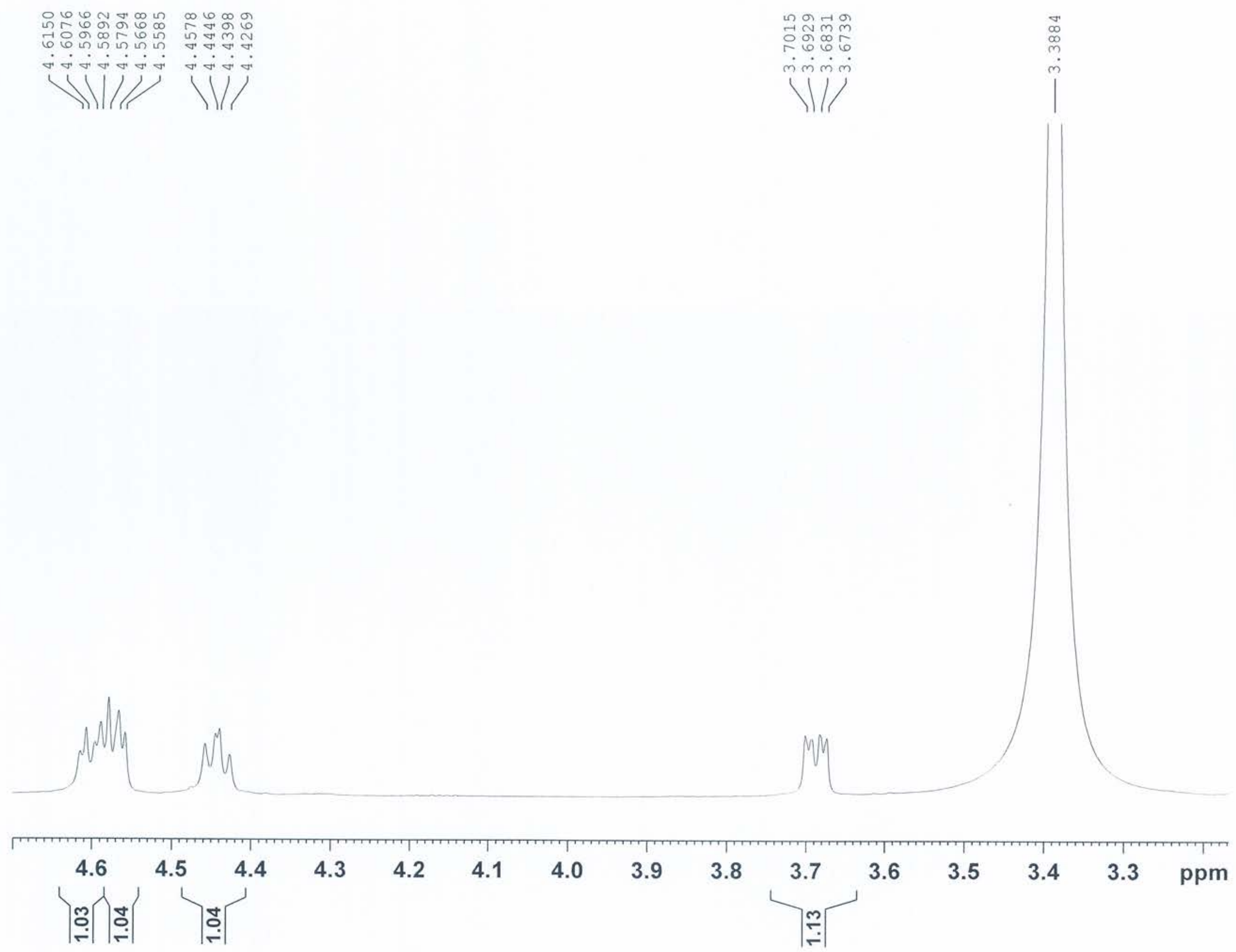

BRUKER

```
Current Data Parameters
NAME      Component 5 of
          Chebulae Fructus_230401
EXPNO                    1
PROCNO                   1

F2 - Acquisition Parameters
Date_             20230930
Time_              16.33 h
INSTRUM             Avance
PROBHD     Z172446_0005 (
PULPROG               zg30
TD                   65536
SOLVENT               DMSO
NS                      16
DS                       2
SWH            11904.762 Hz
FIDRES          0.363304 Hz
AQ             2.7525120 sec
RG               77.3036
DW                42.000 usec
DE                  8.47 usec
TE                 298.1 K
D1            1.00000000 sec
TD0                      1
SFO1         600.1337058 MHz
NUC1                    1H
P0                  3.97 usec
P1                 11.92 usec
PLW1         17.17900085 W

F2 - Processing parameters
SI                   65536
SF           600.1300062 MHz
WDW                     EM
SSB      0
LB                    0.30 Hz
GB       0
PC                    1.00
```

检测人: [illegible] 23.12.07
复核人: [illegible] 23.12.07

HP001

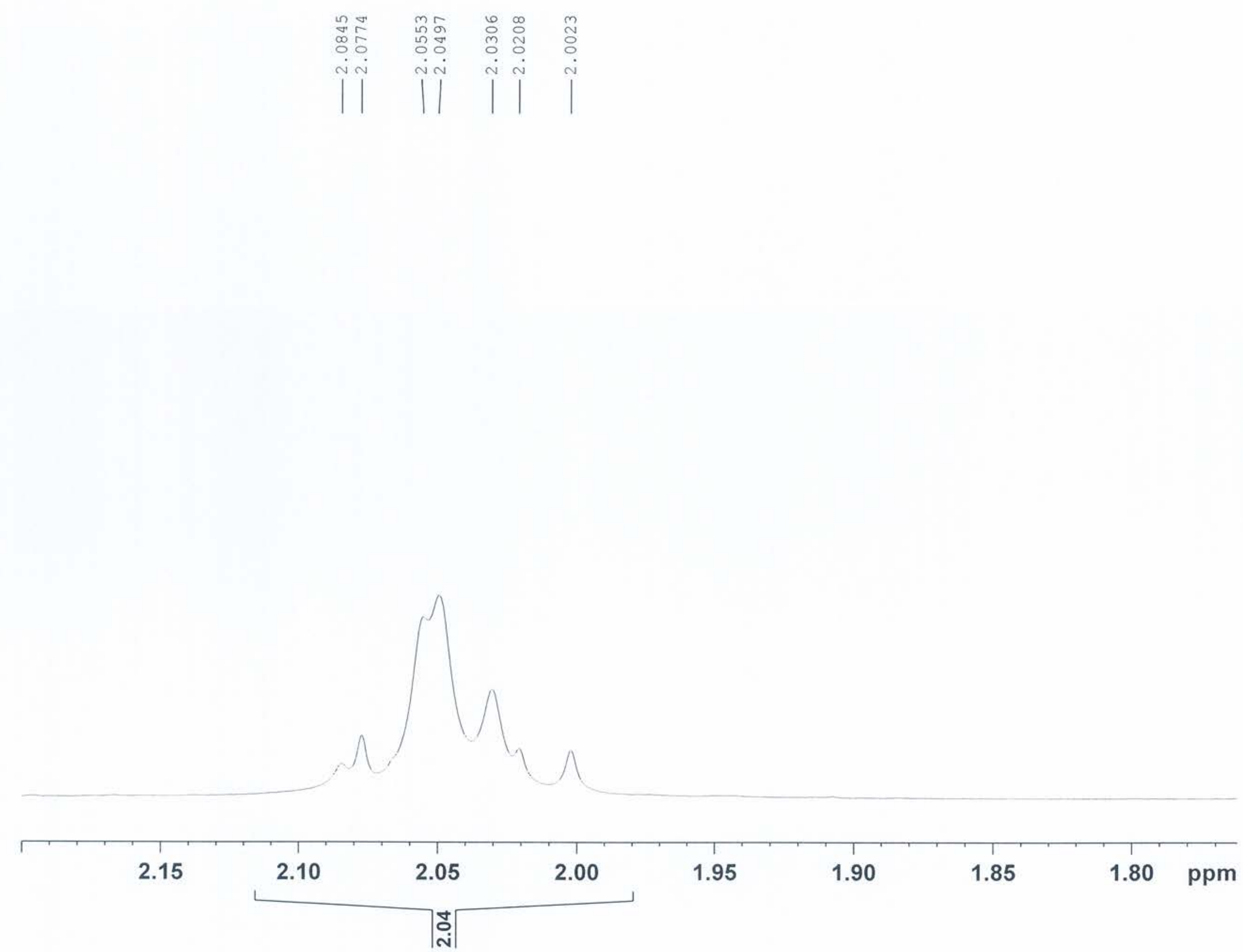

BRUKER

Current Data Parameters	
NAME	Component 5 of Chebulae Fructus_230401
EXPNO	1
PROCNO	1
**F2 - Acquisition Parameters**	
Date_	20230930
Time_	16.33 h
INSTRUM	Avance
PROBHD	Z172446_0005 (
PULPROG	zg30
TD	65536
SOLVENT	DMSO
NS	16
DS	2
SWH	11904.762 Hz
FIDRES	0.363304 Hz
AQ	2.7525120 sec
RG	77.3036
DW	42.000 usec
DE	8.47 usec
TE	298.1 K
D1	1.00000000 sec
TD0	1
SFO1	600.1337058 MHz
NUC1	1H
P0	3.97 usec
P1	11.92 usec
PLW1	17.17900085 W
**F2 - Processing parameters**	
SI	65536
SF	600.1300062 MHz
WDW	EM
SSB	0
LB	0.30 Hz
GB	0
PC	1.00

[illegible]
[illegible]

HP001

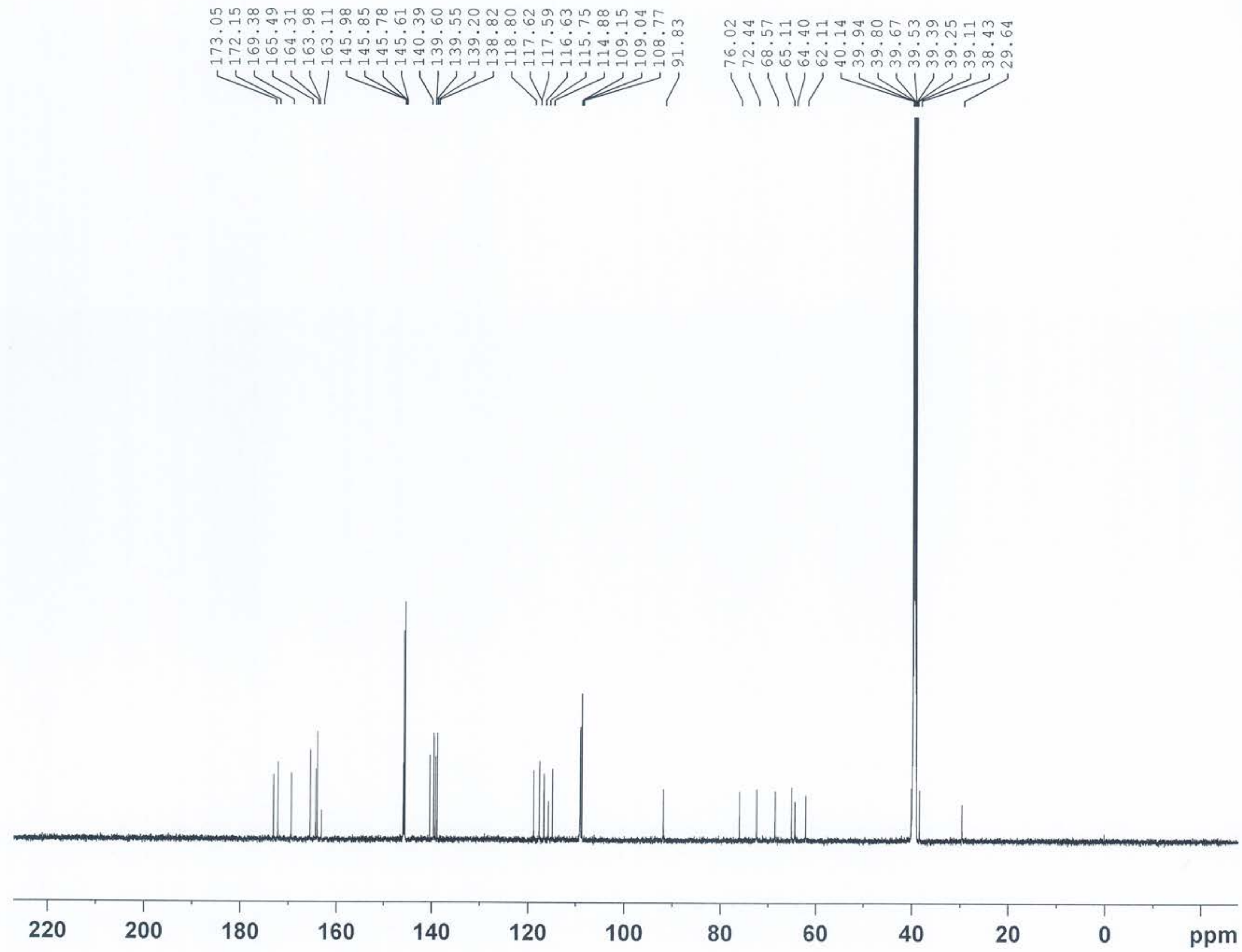

BRUKER

Current Data Parameters
NAME Component 5 of Chebulae Fructus_230401
EXPNO 2
PROCNO 1

F2 - Acquisition Parameters
Date_ 20230930
Time 23.36 h
INSTRUM Avance
PROBHD Z172446_0005 (
PULPROG zgpg30
TD 65536
SOLVENT DMSO
NS 8500
DS 4
SWH 38461.539 Hz
FIDRES 1.173753 Hz
AQ 0.8519680 sec
RG 101
DW 13.000 usec
DE 6.50 usec
TE 298.1 K
D1 2.00000000 sec
D11 0.03000000 sec
TD0 1
SFO1 150.9178988 MHz
NUC1 13C
P0 3.93 usec
P1 11.80 usec
PLW1 86.66300201 W
SFO2 600.1324005 MHz
NUC2 1H
CPDPRG[2 waltz65
PCPD2 70.00 usec
PLW2 17.17900085 W
PLW12 0.49814001 W
PLW13 0.25055999 W

F2 - Processing parameters
SI 32768
SF 150.9028698 MHz
WDW EM
SSB 0
LB 1.00 Hz
GB 0
PC 1.40

检验人: [illegible] 23.12.07
复核人: [illegible] 23.12.07

HP001

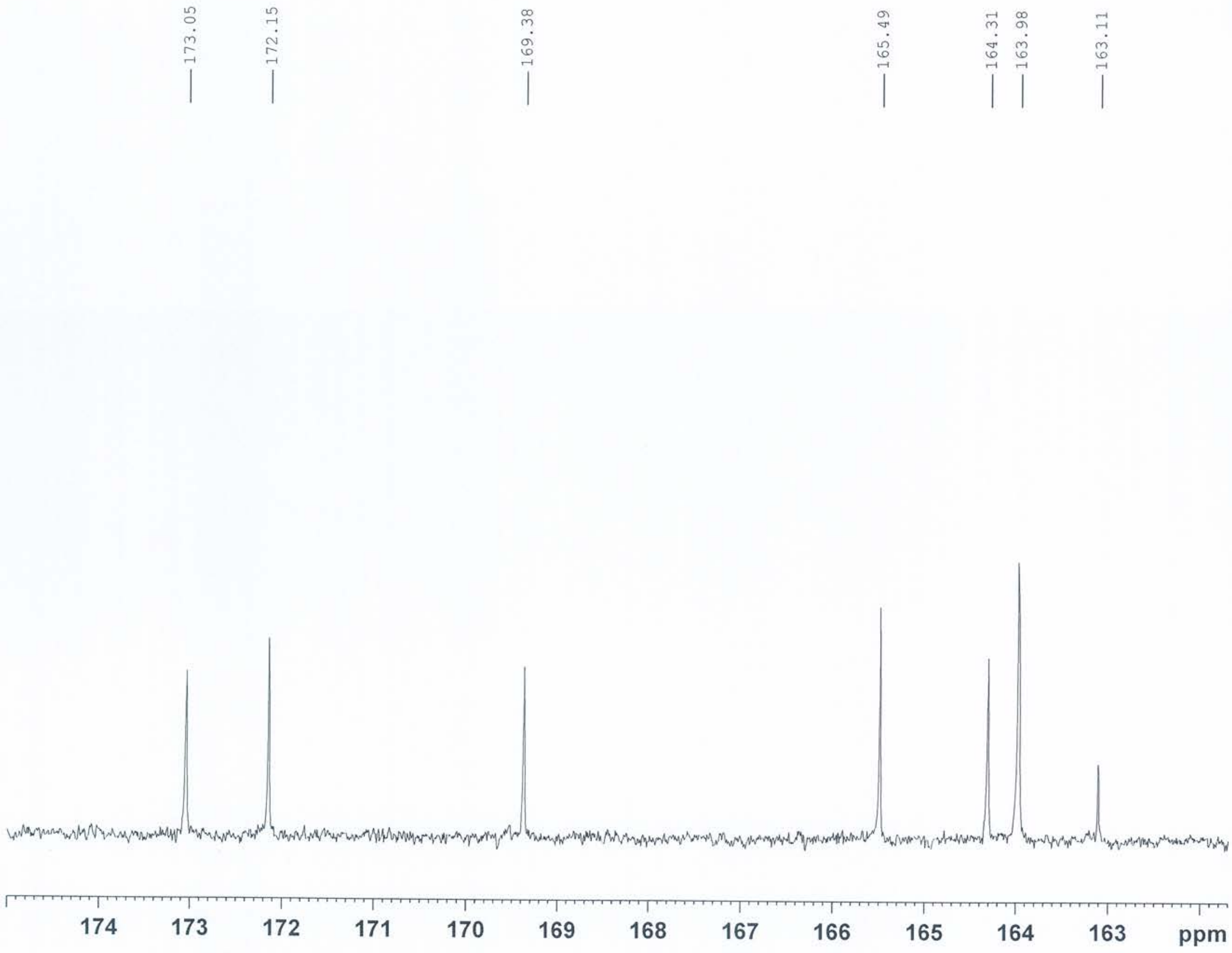

BRUKER

```
Current Data Parameters
NAME      Component 5 of
          Chebulae Fructus_230401
EXPNO                2
PROCNO               1

F2 - Acquisition Parameters
Date_          20230930
Time              23.36 h
INSTRUM          Avance
PROBHD   Z172446_0005 (
PULPROG          zgpg30
TD                65536
SOLVENT            DMSO
NS                 8500
DS                    4
SWH           38461.539 Hz
FIDRES         1.173753 Hz
AQ            0.8519680 sec
RG                  101
DW               13.000 usec
DE                 6.50 usec
TE                298.1 K
D1           2.00000000 sec
D11          0.03000000 sec
TD0                   1
SFO1        150.9178988 MHz
NUC1                13C
P0                 3.93 usec
P1                11.80 usec
PLW1        86.66300201 W
SFO2        600.1324005 MHz
NUC2                 1H
CPDPRG[2         waltz65
PCPD2             70.00 usec
PLW2        17.17900085 W
PLW12        0.49814001 W
PLW13        0.25055999 W

F2 - Processing parameters
SI                32768
SF          150.9028698 MHz
WDW                  EM
SSB      0
LB                 1.00 Hz
GB       0
PC                 1.40
```

检测人: [illegible] 23.12.07
复核人: [illegible] 23.12.07

HP001

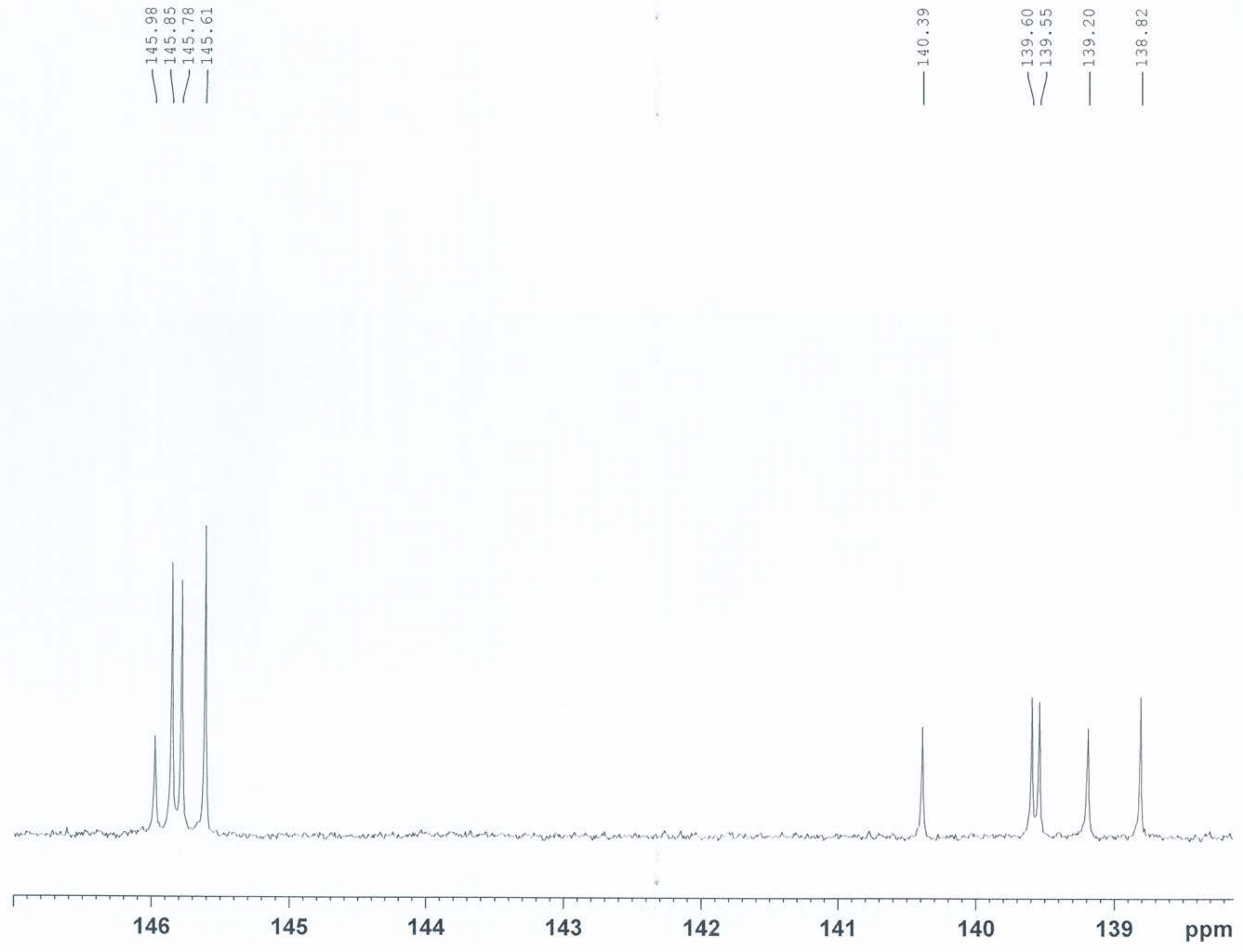

BRUKER

Current Data Parameters
NAME Component 5 of Chebulae Fructus_230401
EXPNO 2
PROCNO 1

F2 - Acquisition Parameters
Date_ 20230930
Time_ 23.36 h
INSTRUM Avance
PROBHD Z172446_0005 (
PULPROG zgpg30
TD 65536
SOLVENT DMSO
NS 8500
DS 4
SWH 38461.539 Hz
FIDRES 1.173753 Hz
AQ 0.8519680 sec
RG 101
DW 13.000 usec
DE 6.50 usec
TE 298.1 K
D1 2.00000000 sec
D11 0.03000000 sec
TD0 1
SFO1 150.9178988 MHz
NUC1 13C
P0 3.93 usec
P1 11.80 usec
PLW1 86.66300201 W
SFO2 600.1324005 MHz
NUC2 1H
CPDPRG[2 waltz65
PCPD2 70.00 usec
PLW2 17.17900085 W
PLW12 0.49814001 W
PLW13 0.25055999 W

F2 - Processing parameters
SI 32768
SF 150.9028698 MHz
WDW EM
SSB 0
LB 1.00 Hz
GB 0
PC 1.40

[illegible] 23.12.07
[illegible] 23.12.07

HP001

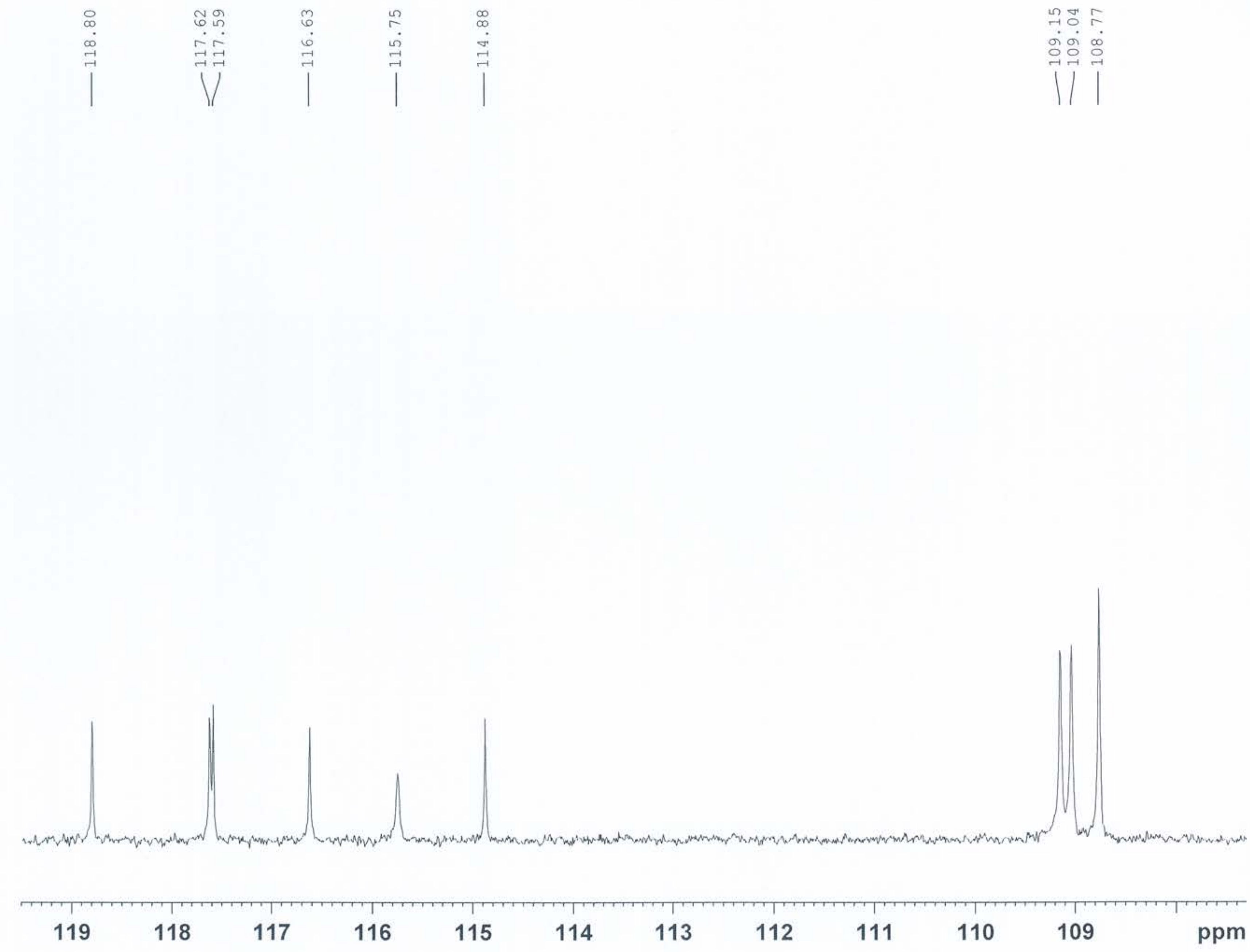

BRUKER

Current Data Parameters

NAME	Component 5 of Chebulae Fructus_230401
EXPNO	2
PROCNO	1

F2 - Acquisition Parameters

Date_	20230930
Time	23.36 h
INSTRUM	Avance
PROBHD	Z172446_0005 (
PULPROG	zgpg30
TD	65536
SOLVENT	DMSO
NS	8500
DS	4
SWH	38461.539 Hz
FIDRES	1.173753 Hz
AQ	0.8519680 sec
RG	101
DW	13.000 usec
DE	6.50 usec
TE	298.1 K
D1	2.00000000 sec
D11	0.03000000 sec
TD0	1
SFO1	150.9178988 MHz
NUC1	13C
P0	3.93 usec
P1	11.80 usec
PLW1	86.66300201 W
SFO2	600.1324005 MHz
NUC2	1H
CPDPRG[2	waltz65
PCPD2	70.00 usec
PLW2	17.17900085 W
PLW12	0.49814001 W
PLW13	0.25055999 W

F2 - Processing parameters

SI	32768
SF	150.9028698 MHz
WDW	EM
SSB	0
LB	1.00 Hz
GB	0
PC	1.40

[illegible] 23.12.07
[illegible] 23.12.07

HP001

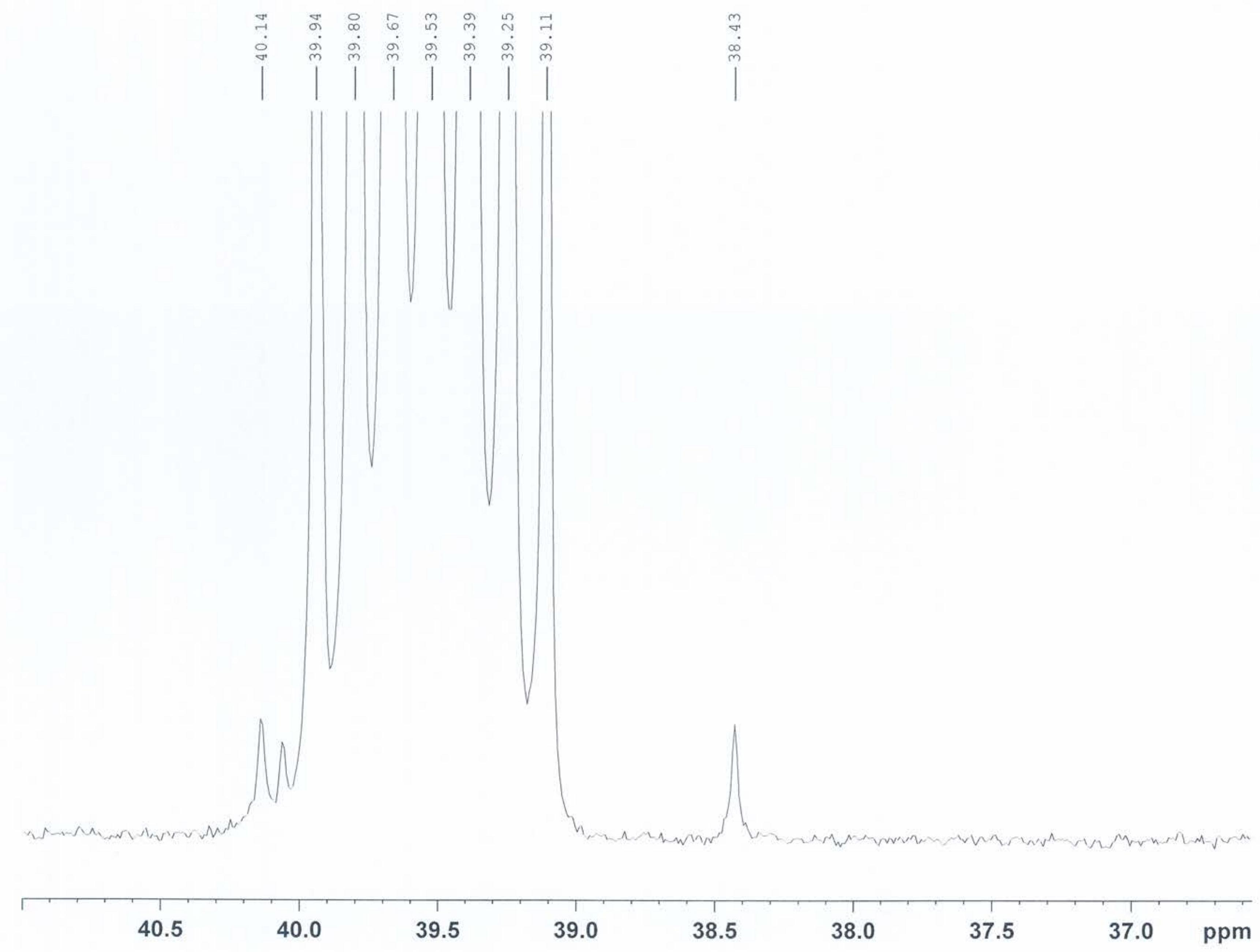

BRUKER

Current Data Parameters

NAME	Component 5 of Chebulae Fructus_230401
EXPNO	2
PROCNO	1

F2 - Acquisition Parameters

Date_	20230930
Time	23.36 h
INSTRUM	Avance
PROBHD	Z172446_0005 (
PULPROG	zgpg30
TD	65536
SOLVENT	DMSO
NS	8500
DS	4
SWH	38461.539 Hz
FIDRES	1.173753 Hz
AQ	0.8519680 sec
RG	101
DW	13.000 usec
DE	6.50 usec
TE	298.1 K
D1	2.00000000 sec
D11	0.03000000 sec
TD0	1
SFO1	150.9178988 MHz
NUC1	13C
P0	3.93 usec
P1	11.80 usec
PLW1	86.66300201 W
SFO2	600.1324005 MHz
NUC2	1H
CPDPRG[2	waltz65
PCPD2	70.00 usec
PLW2	17.17900085 W
PLW12	0.49814001 W
PLW13	0.25055999 W

F2 - Processing parameters

SI	32768
SF	150.9028698 MHz
WDW	EM
SSB	0
LB	1.00 Hz
GB	0
PC	1.40

检测人：[illegible] 23.12.07
复核人：[illegible] 23.12.07

HP001

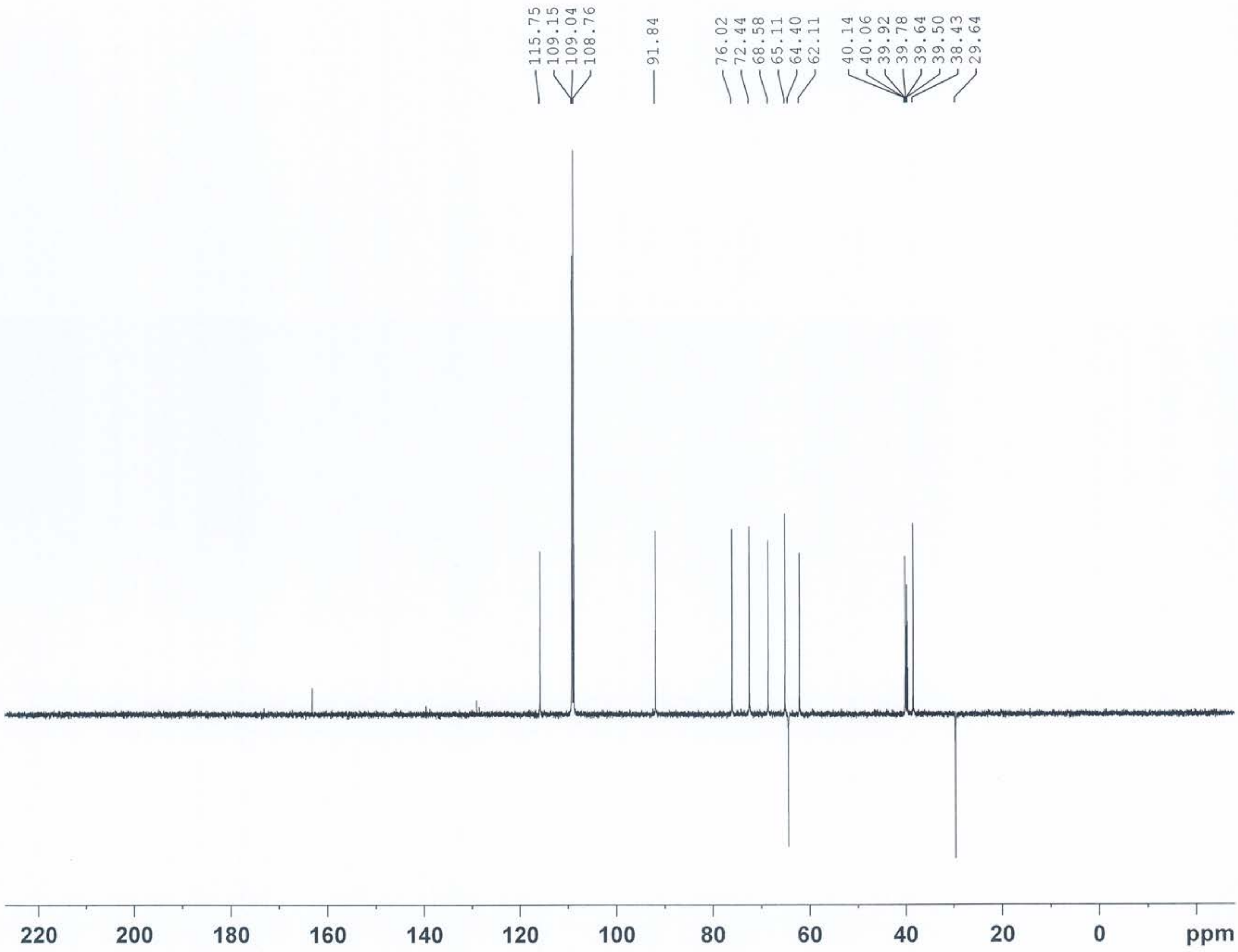

BRUKER

```
Current Data Parameters
NAME     Component 5 of
         Chebulae Fructus_220401
EXPNO                 3
PROCNO                1

F2 - Acquisition Parameters
Date_          20231001
Time               3.15 h
INSTRUM          Avance
PROBHD   Z172446_0005 (
PULPROG       deptsp135
TD                65536
SOLVENT            DMSO
NS                 4500
DS                    8
SWH           38461.539 Hz
FIDRES         1.173753 Hz
AQ            0.8519680 sec
RG                  101
DW               13.000 usec
DE                 6.50 usec
TE                298.1 K
CNST2       145.0000000
D1           2.00000000 sec
D2           0.00344828 sec
D12          0.00002000 sec
TD0                   1
SFO1        150.9178988 MHz
NUC1                13C
P1                11.80 usec
P13             2000.00 usec
PLW0     0 W
PLW1        86.66300201 W
SPNAM[5]    Crp60comp.4
SPOAL5            0.500
SPOFFS5  0 Hz
SPW5        18.43700027 W
SFO2        600.1324005 MHz
NUC2                 1H
CPDPRG[2        waltz65
P3                11.92 usec
P4                23.84 usec
PCPD2             70.00 usec
PLW2        17.17900085 W
PLW12        0.49814001 W

F2 - Processing parameters
SI                32768
SF          150.9028698 MHz
WDW                  EM
SSB      0
LB                 1.00 Hz
GB       0
PC                 1.40
```

校对人：[illegible] 23.12.27
复核人：[illegible] 23.12.27

HP001

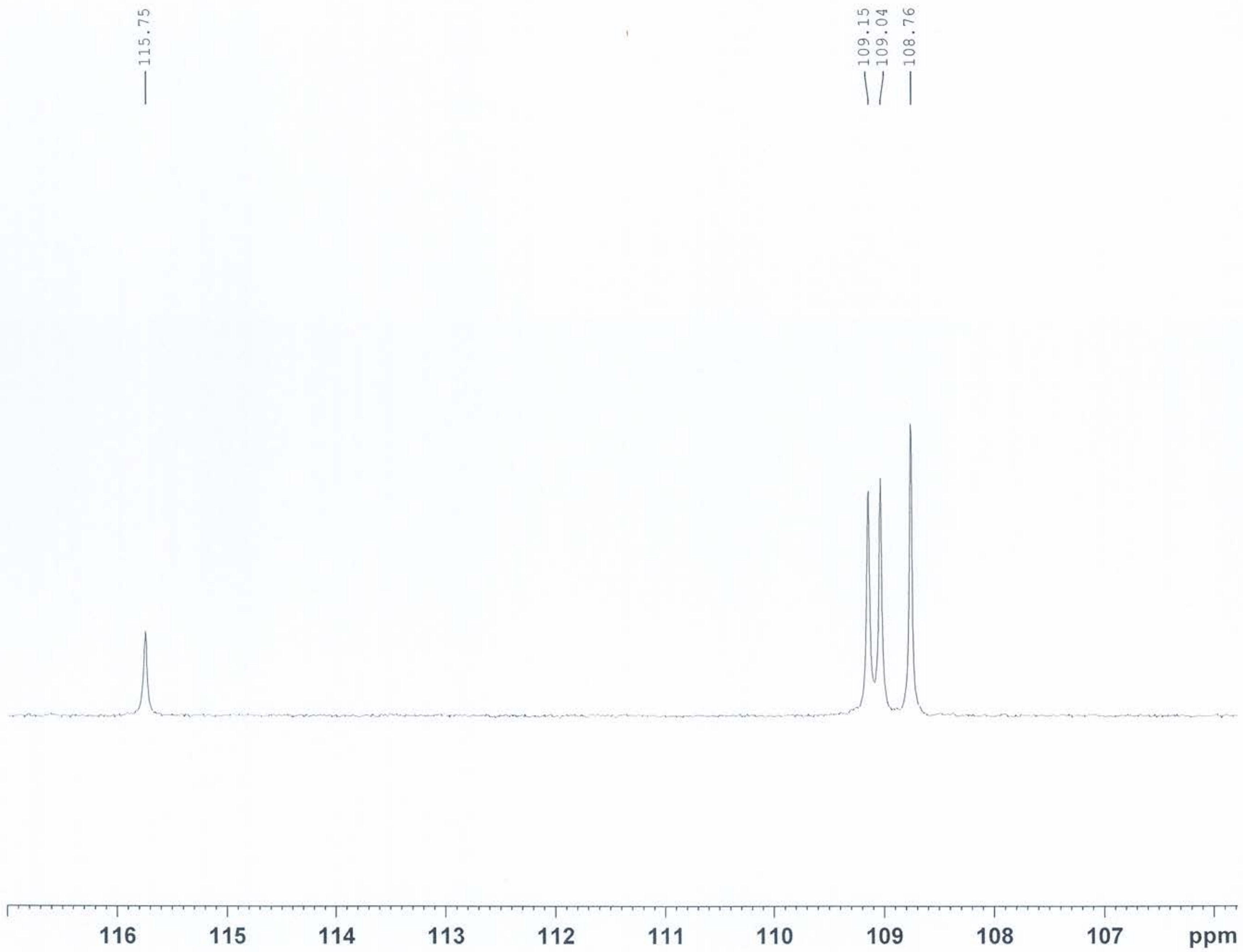

BRUKER

Current Data Parameters
NAME Component b of Chebulae Fructus_230401
EXPNO 3
PROCNO 1

F2 - Acquisition Parameters
Date_ 20231001
Time 3.15 h
INSTRUM Avance
PROBHD Z172446_0005 (
PULPROG deptsp135
TD 65536
SOLVENT DMSO
NS 4500
DS 8
SWH 38461.539 Hz
FIDRES 1.173753 Hz
AQ 0.8519680 sec
RG 101
DW 13.000 usec
DE 6.50 usec
TE 298.1 K
CNST2 145.0000000
D1 2.00000000 sec
D2 0.00344828 sec
D12 0.00002000 sec
TD0 1
SFO1 150.9178988 MHz
NUC1 13C
P1 11.80 usec
P13 2000.00 usec
PLW0 0 W
PLW1 86.66300201 W
SPNAM[5] Crp60comp.4
SPOAL5 0.500
SPOFFS5 0 Hz
SPW5 18.43700027 W
SFO2 600.1324005 MHz
NUC2 1H
CPDPRG[2 waltz65
P3 11.92 usec
P4 23.84 usec
PCPD2 70.00 usec
PLW2 17.17900085 W
PLW12 0.49814001 W

F2 - Processing parameters
SI 32768
SF 150.9028499 MHz
WDW EM
SSB 0
LB 1.00 Hz
GB 0
PC 1.40

校对人：[illegible] 23.12.07
复核人：[illegible] 23.12.07

HP001

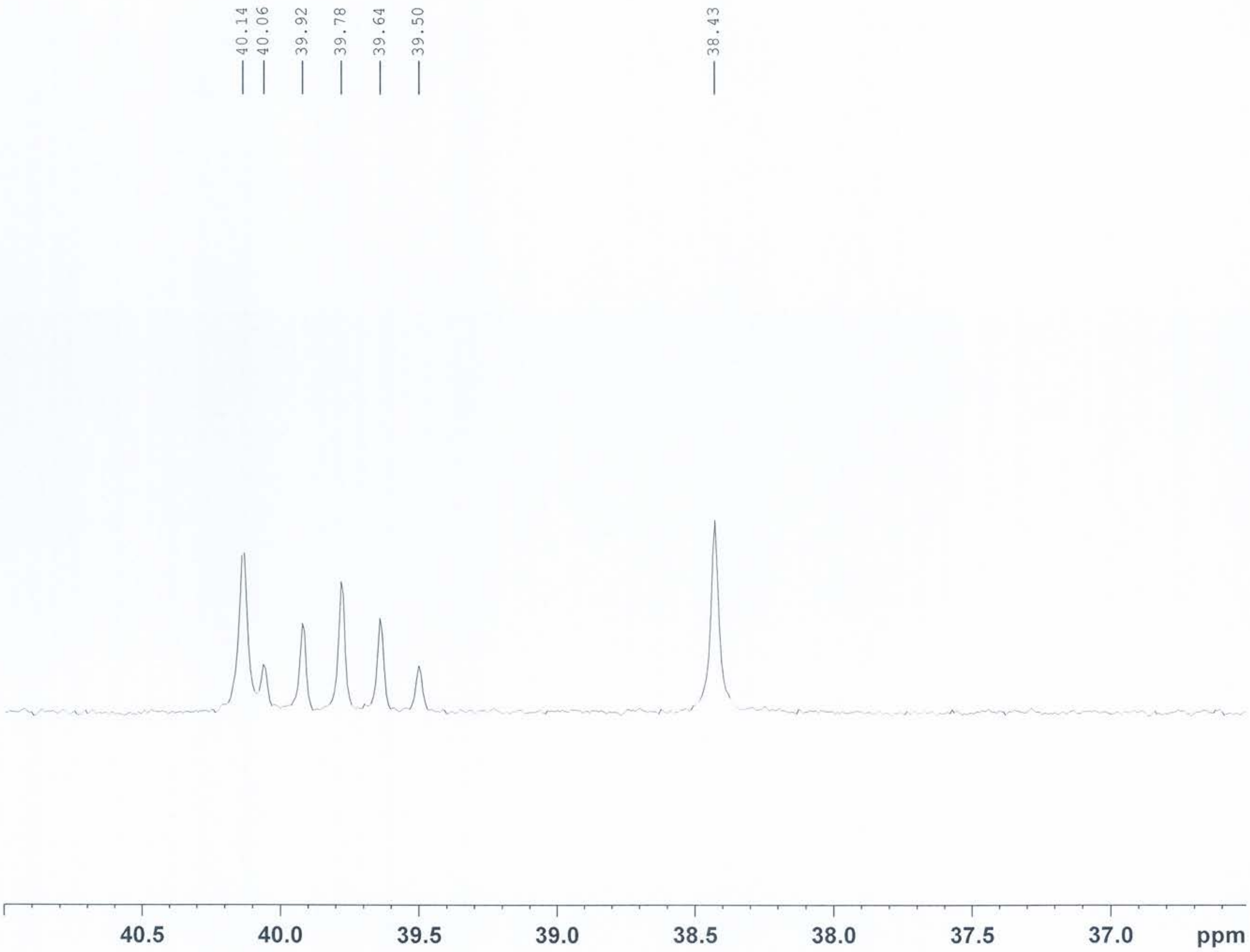

BRUKER

Current Data Parameters	
NAME	Component 5 of Chebulae Fructus_230401
EXPNO	3
PROCNO	1
F2 - Acquisition Parameters	
Date_	20231001
Time	3.15 h
INSTRUM	Avance
PROBHD	Z172446_0005 (
PULPROG	deptsp135
TD	65536
SOLVENT	DMSO
NS	4500
DS	8
SWH	38461.539 Hz
FIDRES	1.173753 Hz
AQ	0.8519680 sec
RG	101
DW	13.000 usec
DE	6.50 usec
TE	298.1 K
CNST2	145.0000000
D1	2.00000000 sec
D2	0.00344828 sec
D12	0.00002000 sec
TD0	1
SFO1	150.9178928 MHz
NUC1	13C
P1	11.80 usec
P13	2000.00 usec
PLW0	0 W
PLW1	86.66300201 W
SPNAM[5]	Crp60comp.4
SPOAL5	0.500
SPOFFS5	0 Hz
SPW5	18.43700027 W
SFO2	600.1324005 MHz
NUC2	1H
CPDPRG[2]	waltz65
P3	11.92 usec
P4	23.84 usec
PCPD2	70.00 usec
PLW2	17.17900085 W
PLW12	0.49814001 W
F2 - Processing parameters	
SI	32768
SF	150.9028698 MHz
WDW	EM
SSB	0
LB	1.00 Hz
GB	0
PC	1.40

检验人：[illegible] 23.12.07
复核人：[illegible] 23.12.07

HP001

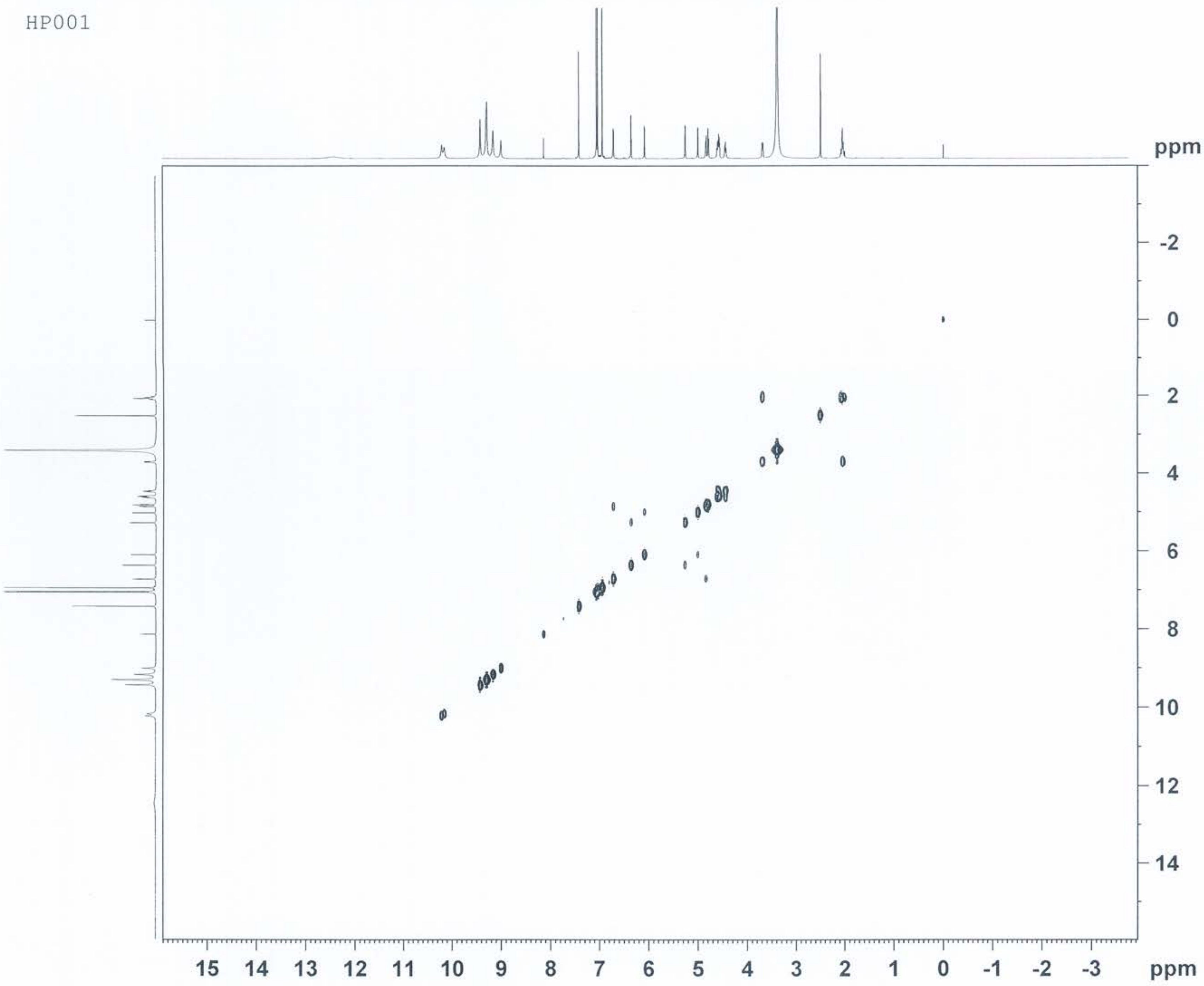

BRUKER

Current Data Parameters	
NAME	Component 5 of Chebulae Fructus_230401
EXPNO	4
PROCNO	1
F2 - Acquisition Parameters	
Date_	20231001
Time	4.10 h
INSTRUM	Avance
PROBHD	Z172446_0005 (
PULPROG	cosygpppqf
TD	2048
SOLVENT	DMSO
NS	12
DS	16
SWH	11904.762 Hz
FIDRES	11.625744 Hz
AQ	0.0860160 sec
RG	101
DW	42.000 usec
DE	6.50 usec
TE	298.1 K
D0	0.00000300 sec
D1	2.00000000 sec
D11	0.03000000 sec
D12	0.00002000 sec
D13	0.00000400 sec
D16	0.00020000 sec
IN0	0.00008331 sec
TDav	1
SFO1	600.1336008 MHz
NUC1	1H
P0	11.92 usec
P1	11.92 usec
P17	2500.00 usec
PLW1	17.17900085 W
PLW10	3.90540004 W
GPNAM[1]	SMSQ10.100
GPZ1	10.00 %
P16	1000.00 usec
F1 - Acquisition parameters	
TD	128
SFO1	600.1336 MHz
FIDRES	187.546890 Hz
SW	20.001 ppm
FnMODE	QF
F2 - Processing parameters	
SI	1024
SF	600.1300062 MHz
WDW	QSINE
SSB	0
LB	0 Hz
GB	0
PC	1.40
F1 - Processing parameters	
SI	1024
MC2	QF
SF	600.1300062 MHz
WDW	QSINE
SSB	0
LB	0 Hz
GB	0

检测人：[illegible] 23.12.27
复核人：[illegible] 23.12.27

HP001

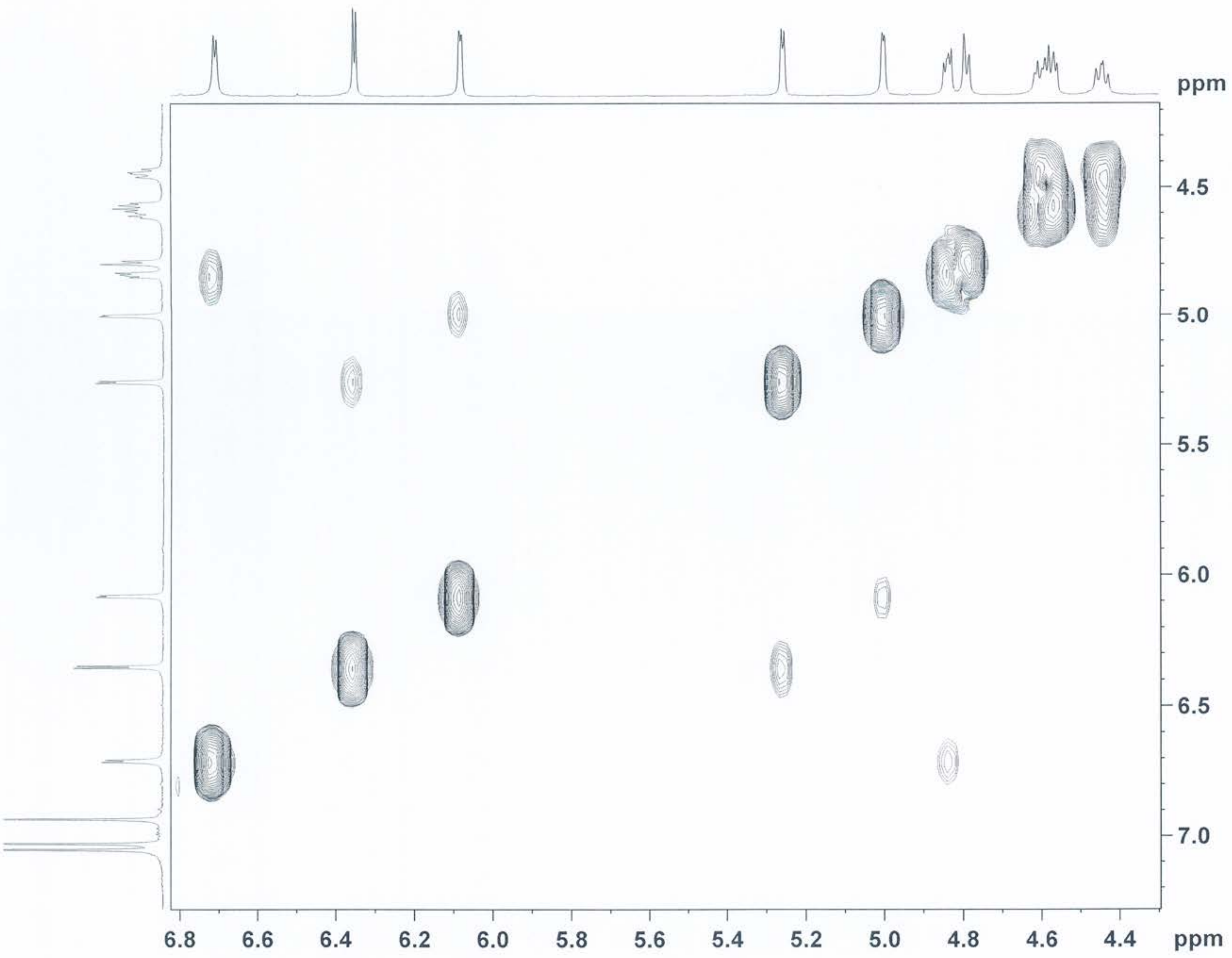

BRUKER

Current Data Parameters	
NAME	Component 5 of Chebulae Fructus_230401
EXPNO	4
PROCNO	1
F2 - Acquisition Parameters	
Date_	20231001
Time_	4.10 h
INSTRUM	Avance
PROBHD	Z172446_0005 (
PULPROG	cosygpppqf
TD	2048
SOLVENT	DMSO
NS	12
DS	16
SWH	11904.762 Hz
FIDRES	11.625744 Hz
AQ	0.0860160 sec
RG	101
DW	42.000 usec
DE	6.50 usec
TE	298.1 K
D0	0.00000300 sec
D1	2.00000000 sec
D11	0.03000000 sec
D12	0.00002000 sec
D13	0.00000400 sec
D16	0.00020000 sec
IN0	0.00008331 sec
TDav	1
SFO1	600.1336008 MHz
NUC1	1H
P0	11.92 usec
P1	11.92 usec
P17	2500.00 usec
PLW1	17.17900085 W
PLW10	3.90540004 W
GPNAM[1]	SMSQ10.100
GPZ1	10.00 %
P16	1000.00 usec
F1 - Acquisition parameters	
TD	128
SFO1	600.1336 MHz
FIDRES	187.546890 Hz
SW	20.001 ppm
FnMODE	QF
F2 - Processing parameters	
SI	1024
SF	600.1300062 MHz
WDW	QSINE
SSB	0
LB	0 Hz
GB	0
PC	1.40
F1 - Processing parameters	
SI	1024
MC2	QF
SF	600.1300062 MHz
WDW	QSINE
SSB	0
LB	0 Hz
GB	0

检验人：[illegible] 23.12.07
复核人：[illegible] 23.12.07

HP001

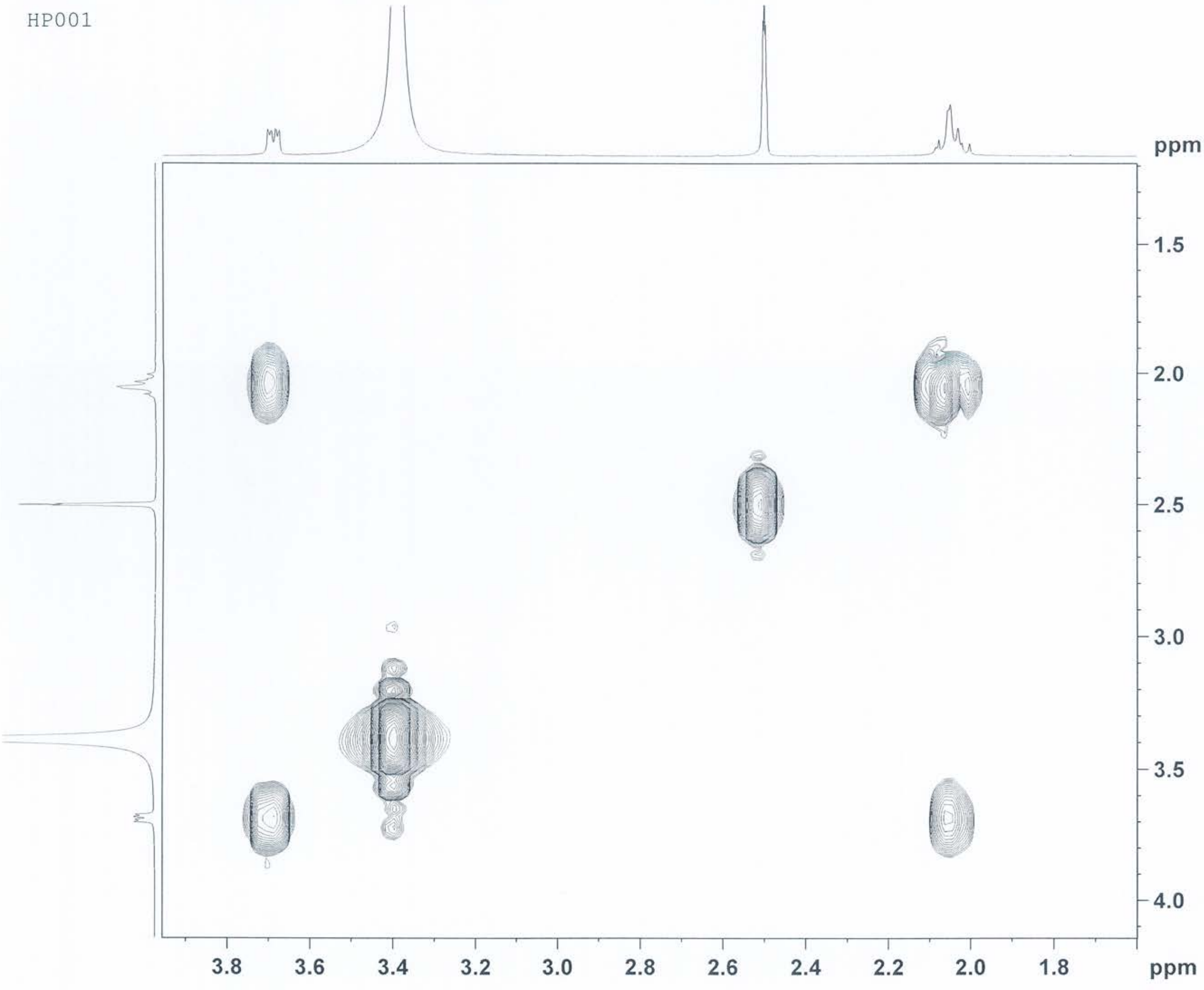


```
Current Data Parameters
NAME        Component 5 of
            Chebulae Fructus_230401
EXPNO                    4
PROCNO                   1

F2 - Acquisition Parameters
Date_            20231001
Time                 4.10 h
INSTRUM            Avance
PROBHD   Z172446_0005 (
PULPROG        cosygpppqf
TD                   2048
SOLVENT              DMSO
NS                     12
DS                     16
SWH             11904.762 Hz
FIDRES          11.625744 Hz
AQ              0.0860160 sec
RG                    101
DW                 42.000 usec
DE                   6.50 usec
TE                  298.1 K
D0             0.00000300 sec
D1             2.00000000 sec
D11            0.03000000 sec
D12            0.00002000 sec
D13            0.00000400 sec
D16            0.00020000 sec
IN0            0.00008331 sec
TDav                    1
SFO1          600.1336008 MHz
NUC1                   1H
P0                  11.92 usec
P1                  11.92 usec
P17               2500.00 usec
PLW1          17.17900085 W
PLW10          3.90540004 W
GPNAM[1]       SMSQ10.100
GPZ1                10.00 %
P16               1000.00 usec

F1 - Acquisition parameters
TD                    128
SFO1             600.1336 MHz
FIDRES         187.546890 Hz
SW                 20.001 ppm
FnMODE                 QF

F2 - Processing parameters
SI                   1024
SF            600.1300062 MHz
WDW                 QSINE
SSB       0
LB        0 Hz
GB        0
PC                   1.40

F1 - Processing parameters
SI                   1024
MC2                    QF
SF            600.1300062 MHz
WDW                 QSINE
SSB       0
LB        0 Hz
GB        0
```

检测人：[illegible] 23.12.07
复核人：[illegible] 23.12.07

HP001

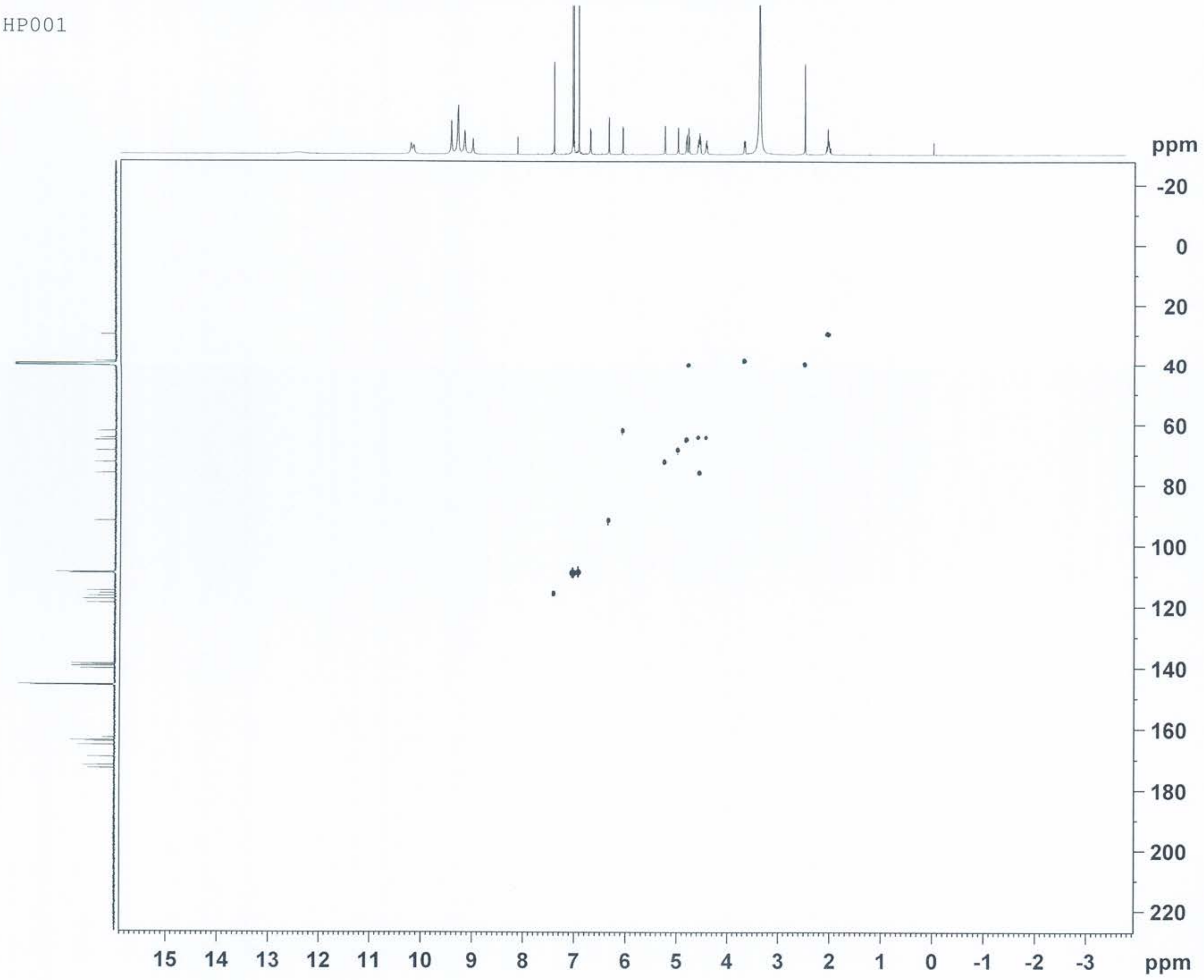

BRUKER

```
Current Data Parameters
NAME         Component 5 of
             Chebulae Fructus_230401
EXPNO                      5
PROCNO                     1

F2 - Acquisition Parameters
Date_               20231001
Time                  6.01 h
INSTRUM               Avance
PROBHD       Z172446_0005 (
PULPROG      hsqcedetgpsisp2.3
TD                      2048
SOLVENT                 DMSO
NS                        12
DS                        32
SWH               11904.762 Hz
FIDRES            11.625744 Hz
AQ                0.0860160 sec
RG                       101
DW                   42.000 usec
DE                     6.50 usec
TE                    298.2 K
CNST2           145.0000000
CNST17           -0.5000000
D0               0.00000300 sec
D1               2.00000000 sec
D4               0.00172414 sec
D11              0.03000000 sec
D16              0.00020000 sec
D21              0.00360000 sec
D24              0.00089000 sec
IN0              0.00001300 sec
TDav                       1
SFO1           600.1336008 MHz
NUC1                      1H
P1                     11.92 usec
P2                     23.84 usec
P28                  1000.00 usec
PLW1             17.17900085 W
SFO2           150.9178988 MHz
NUC2                     13C
CPDPRG[2 bi_p5m4sp_4sp.2
P3                     11.80 usec
P14                   500.00 usec
P24                  2000.00 usec
P31                  1730.00 usec
P63                  1500.00 usec
PLW0         0 W
PLW2             86.66300201 W
PLW12             3.35190010 W
P16                  1000.00 usec
P19                   600.00 usec

F1 - Acquisition parameters
TD                       256
SFO1              150.9179 MHz
FIDRES          300.625305 Hz
SW                 254.973 ppm
FnMODE      Echo-Antiecho

F2 - Processing parameters
SI                      1024
SF             600.1300062 MHz
WDW                    QSINE
SSB                        2
LB           0 Hz
GB           0
PC                      1.40

F1 - Processing parameters
SI                      1024
MC2           echo-antiecho
SF             150.9028698 MHz
WDW                    QSINE
SSB                        2
LB           0 Hz
GB           0
```

核对人：[illegible] 23.12.07

复核人：[illegible] 23.12.07

HP001

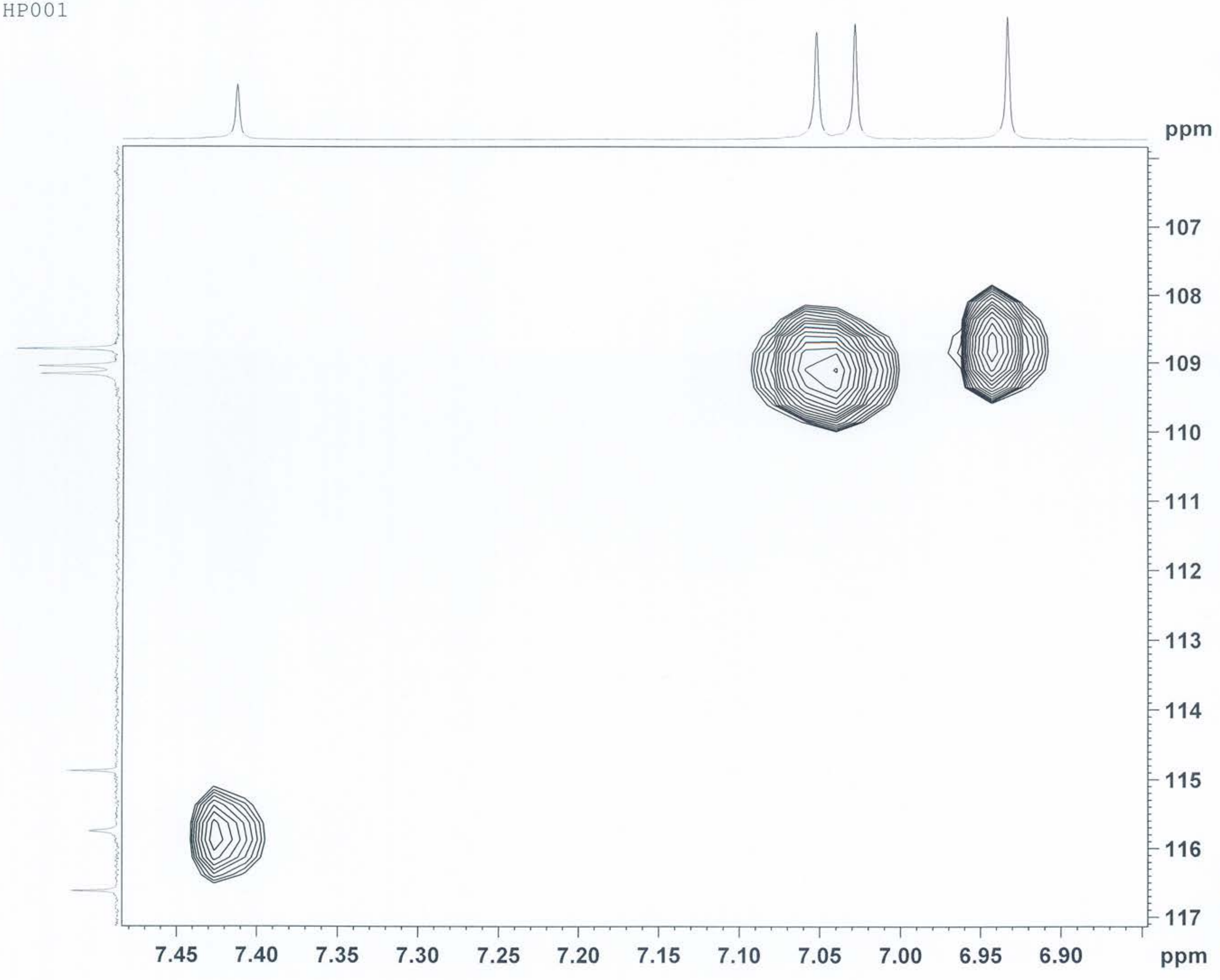

BRUKER

```
Current Data Parameters
NAME      Component 5 of
          Chebulae Fructus_230401
EXPNO                5
PROCNO               1

F2 - Acquisition Parameters
Date_           20231001
Time                6.01 h
INSTRUM           Avance
PROBHD   Z172444_0005 (
PULPROG  hsqcedetgpsisp2.3
TD                  2048
SOLVENT             DMSO
NS                    12
DS                    32
SWH           11904.762 Hz
FIDRES        11.625744 Hz
AQ            0.0860160 sec
RG                   101
DW                42.000 usec
DE                  6.50 usec
TE                 298.2 K
CNST2        145.0000000
CNST17        -0.5000000
D0            0.00000300 sec
D1            2.00000000 sec
D4            0.00172414 sec
D11           0.03000000 sec
D16           0.00020000 sec
D21           0.00360000 sec
D24           0.00089000 sec
IN0           0.00001200 sec
TDav                   1
SFO1         600.1236008 MHz
NUC1                  1H
P1                 11.92 usec
P2                 23.84 usec
P28              1000.00 usec
PLW1         17.17900085 W
SFO2         150.9178988 MHz
NUC2                 13C
CPDPRG[2  bi_p5m4sp_4sp.2
P3                 11.80 usec
P14               500.00 usec
P24              2000.00 usec
P31              1730.00 usec
P63              1500.00 usec
PLW0      0 W
PLW2         86.66100201 W
PLW12         3.35190010 W
P16              1000.00 usec
P19               600.00 usec

F1 - Acquisition parameters
TD                   256
SFO1            150.9179 MHz
FIDRES        400.625205 Hz
SW               254.973 ppm
FnMODE      Echo-Antiecho

F2 - Processing parameters
SI                  1024
SF           600.1300062 MHz
WDW                QSINE
SSB                    2
LB          0 Hz
GB          0
PC                  1.40

F1 - Processing parameters
SI                  1024
MC2         echo-antiecho
SF           150.9028699 MHz
WDW                QSINE
SSB                    2
LB          0 Hz
GB          0
```

检测人：[illegible] 23.12.07
复核人：[illegible] 23.12.07

HP001

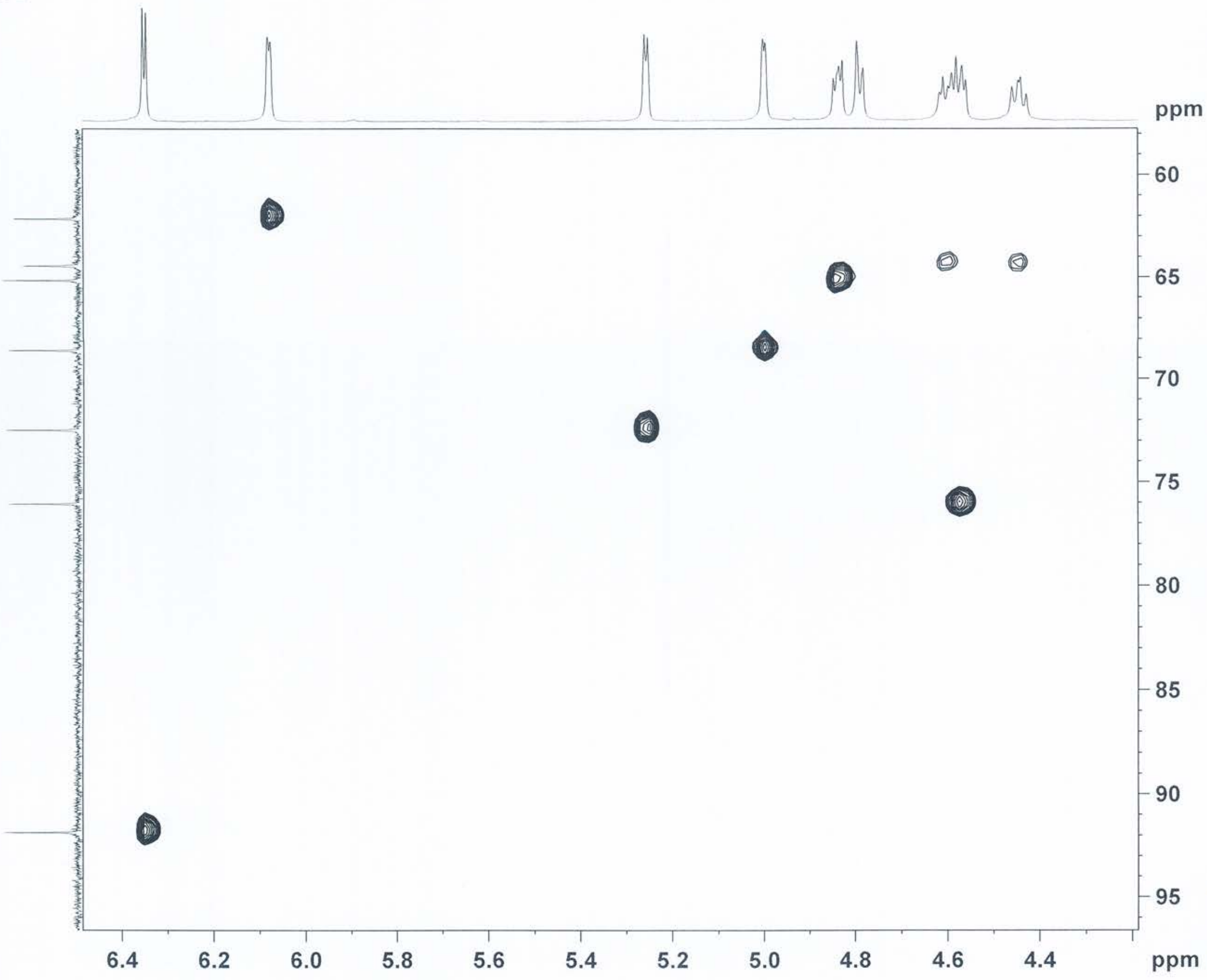

BRUKER

```
Current Data Parameters
NAME      Component 5 of
          Chebulae Fructus_230401
EXPNO                 5
PROCNO                1

F2 - Acquisition Parameters
Date_          20231001
Time               6.01 h
INSTRUM          Avance
PROBHD   Z172446_0005 (
PULPROG  hsqcedetgpsisp2.3
TD                 2048
SOLVENT            DMSO
NS                   12
DS                   32
SWH           11904.762 Hz
FIDRES        11.625744 Hz
AQ            0.0860160 sec
RG                  101
DW               42.000 usec
DE                 6.50 usec
TE                298.2 K
CNST2       145.0000000
CNST17       -0.5000000
D0           0.00000300 sec
D1           2.00000000 sec
D4           0.00172414 sec
D11          0.03000000 sec
D16          0.00020000 sec
D21          0.00360000 sec
D24          0.00089000 sec
IN0          0.00001300 sec
TDav                  1
SFO1        600.1336008 MHz
NUC1                 1H
P1                11.92 usec
P2                23.84 usec
P28             1000.00 usec
PLW1        17.17900085 W
SFO2        150.9178988 MHz
NUC2                13C
CPDPRG[2 bi_p5m4sp_4sp.2
P3                11.80 usec
P14              500.00 usec
P24             2000.00 usec
P31             1730.00 usec
P63             1500.00 usec
PLW0      0 W
PLW2        86.66300201 W
PLW12        3.35190010 W
P16             1000.00 usec
P19              600.00 usec

F1 - Acquisition parameters
TD                  256
SFO1           150.9179 MHz
FIDRES       300.625305 Hz
SW              254.973 ppm
FnMODE     Echo-Antiecho

F2 - Processing parameters
SI                 1024
SF          600.1300062 MHz
WDW               QSINE
SSB                   2
LB        0 Hz
GB        0
PC                 1.40

F1 - Processing parameters
SI                 1024
MC2         echo-antiecho
SF          150.9028698 MHz
WDW               QSINE
SSB                   2
LB        0 Hz
GB        0
```

[illegible]：[illegible] 23.12.07

复核人：[illegible] 23.12.07

HP001

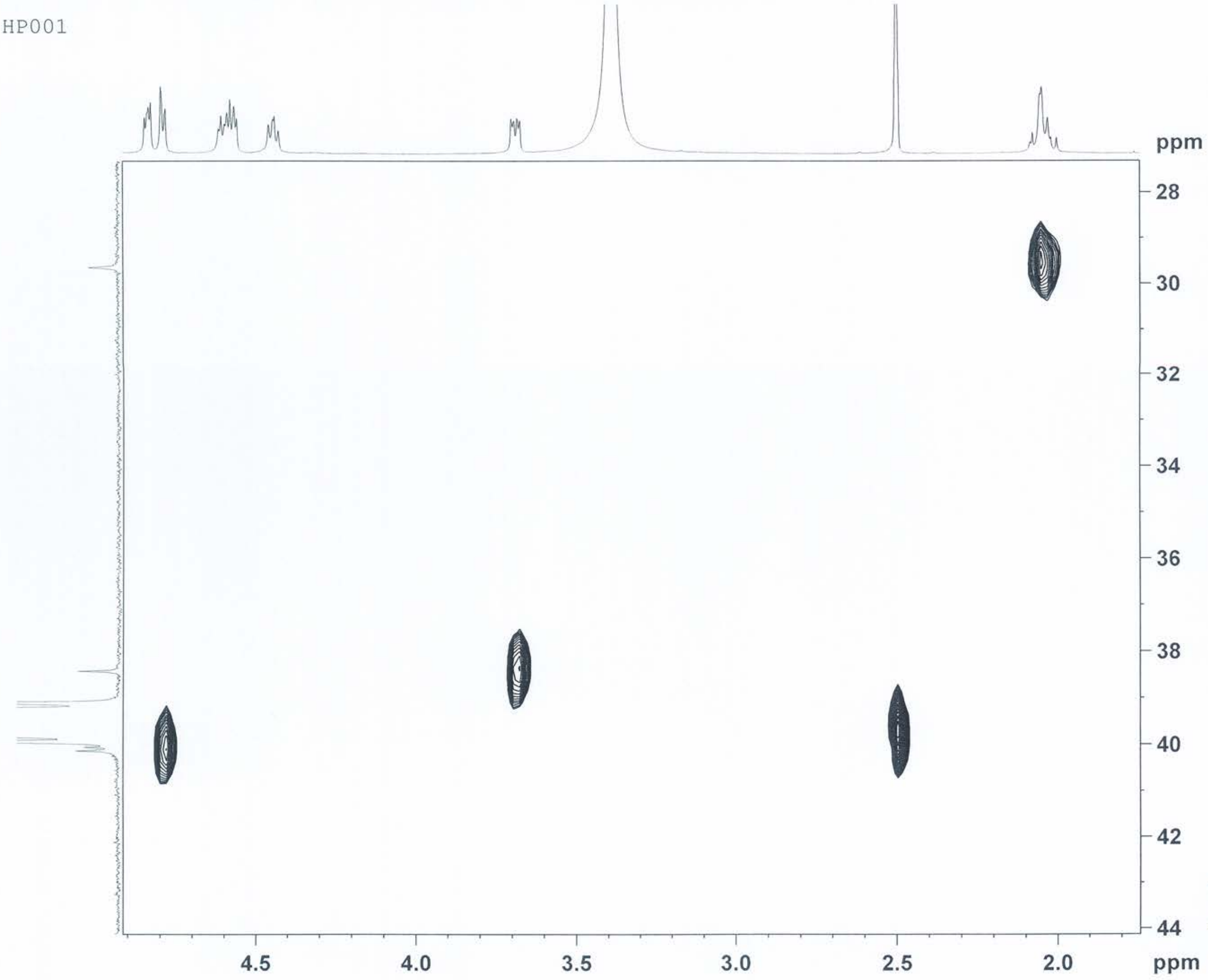


```
Current Data Parameters
NAME      Component 5 of
          Chebulae Fructus_230401
EXPNO                   5
PROCNO                  1

F2 - Acquisition Parameters
Date_            20231001
Time                 6.21 h
INSTRUM            Avance
PROBHD   Z172446_0005 (
PULPROG  hsqcedetgpsisp2.3
TD                   2048
SOLVENT              DMSO
NS                     12
DS                     32
SWH             11904.762 Hz
FIDRES          11.625744 Hz
AQ              0.0860160 sec
RG                    101
DW                 42.000 usec
DE                   6.50 usec
TE                  298.2 K
CNST2         145.0000000
CNST17         -0.5000000
D0             0.00000300 sec
D1             2.00000000 sec
D4             0.00172414 sec
D11            0.03000000 sec
D16            0.00020000 sec
D21            0.00360000 sec
D24            0.00089000 sec
IN0            0.00001300 sec
TDav                    1
SFO1          600.1336028 MHz
NUC1                   1H
P1                  11.92 usec
P2                  23.84 usec
P28               1000.00 usec
PLW1          17.17900085 W
SFO2          150.9178988 MHz
NUC2                  13C
CPDPRG[2  p1_p5m4sp_4sp.2
P3                  11.80 usec
P14                500.00 usec
P24               2000.00 usec
P31               1730.00 usec
P63               1500.00 usec
PLW0      0 W
PLW2          86.66300201 W
PLW12          3.35190010 W
P16               1000.00 usec
P19                600.00 usec

F1 - Acquisition parameters
TD                    256
SFO1             150.9179 MHz
FIDRES         300.625301 Hz
SW                254.973 ppm
FnMODE       Echo-Antiecho

F2 - Processing parameters
SI                   1024
SF            600.1300062 MHz
WDW                 QSINE
SSB                     2
LB        0 Hz
GB        0
PC                   1.40

F1 - Processing parameters
SI                   1024
MC2          echo-antiecho
SF            150.9028699 MHz
WDW                 QSINE
SSB                     2
LB        0 Hz
GB        0
```

检测人：[illegible] 23.12.07
复核人：[illegible] 23-12-07

HP001

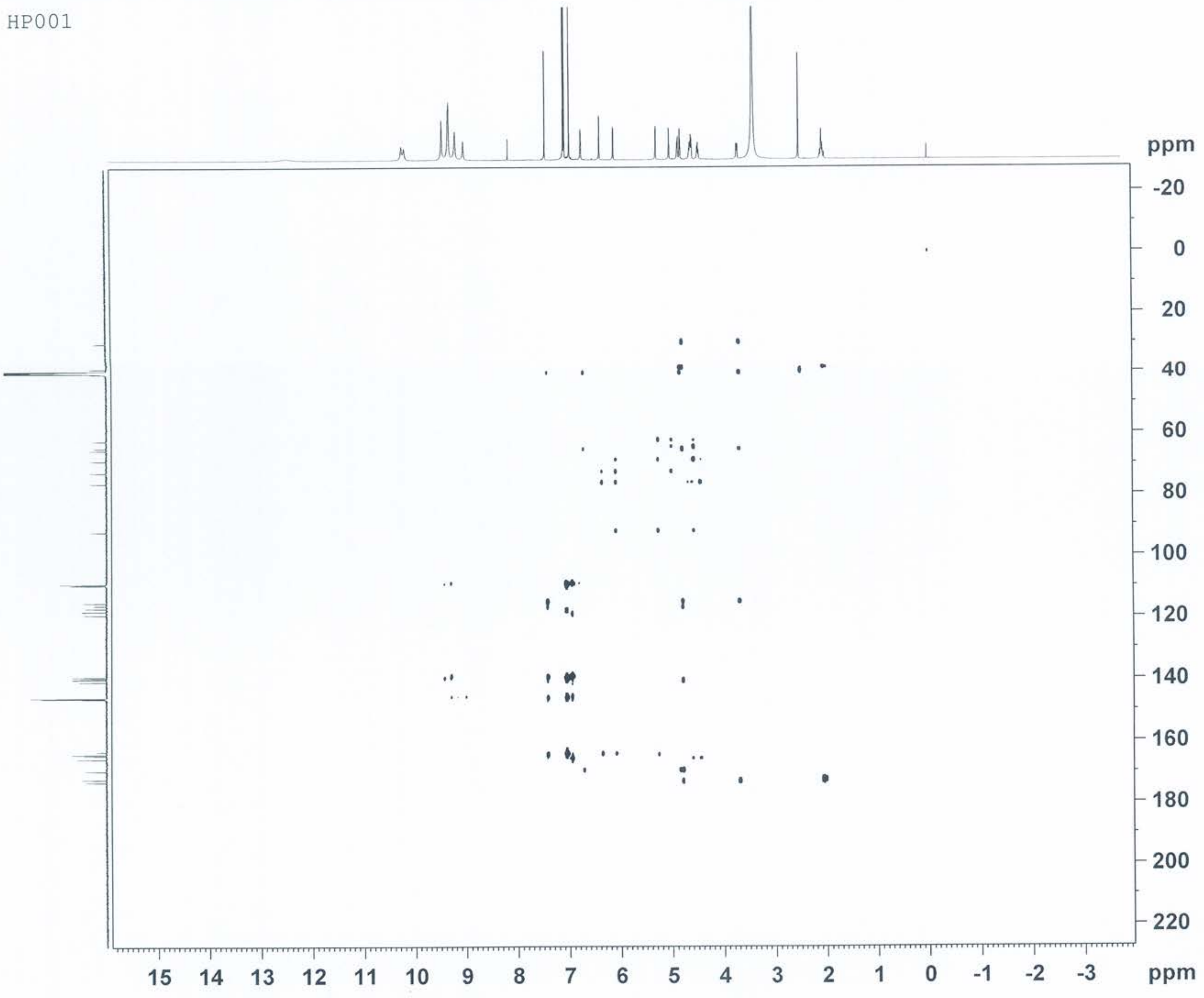

BRUKER

Current Data Parameters
NAME Component 5 of Chebulae Fructus_230401
EXPNO 6
PROCNO 1

F2 - Acquisition Parameters
Date_ 20231001
Time 16.17 h
INSTRUM Avance
PROBHD Z172446_0005 (
PULPROG hmbcetgpl3nd
TD 4096
SOLVENT DMSO
NS 64
DS 16
SWH 11904.762 Hz
FIDRES 5.812872 Hz
AQ 0.1720320 sec
RG 101
DW 42.000 usec
DE 6.50 usec
TE 298.2 K
CNST6 120.0000000
CNST7 170.0000000
CNST13 8.0000000
D0 0.00000300 sec
D1 2.00000000 sec
D6 0.06250000 sec
D16 0.00020000 sec
IN0 0.00001300 sec
TDav 1
SFO1 600.1336008 MHz
NUC1 1H
P1 11.92 usec
P2 23.84 usec
PLW1 17.17900085 W
SFO2 150.9178988 MHz
NUC2 13C
P3 11.80 usec
P24 2000.00 usec
PLW2 86.66300201 W
P16 1000.00 usec
CNST30 0.598116

F1 - Acquisition parameters
TD 256
SFO1 150.9179 MHz
FIDRES 300.625305 Hz
SW 254.973 ppm
FnMODE Echo-Antiecho

F2 - Processing parameters
SI 2048
SF 600.1300140 MHz
WDW SINE
SSB 4
LB 0 Hz
GB 0
PC 1.40

F1 - Processing parameters
SI 1024
MC2 echo-antiecho
SF 150.9028720 MHz
WDW QSINE
SSB 2
LB 0 Hz
GB 0

核验人: [illegible] 23.12.07
复核人: [illegible] 23.12.07

HP001

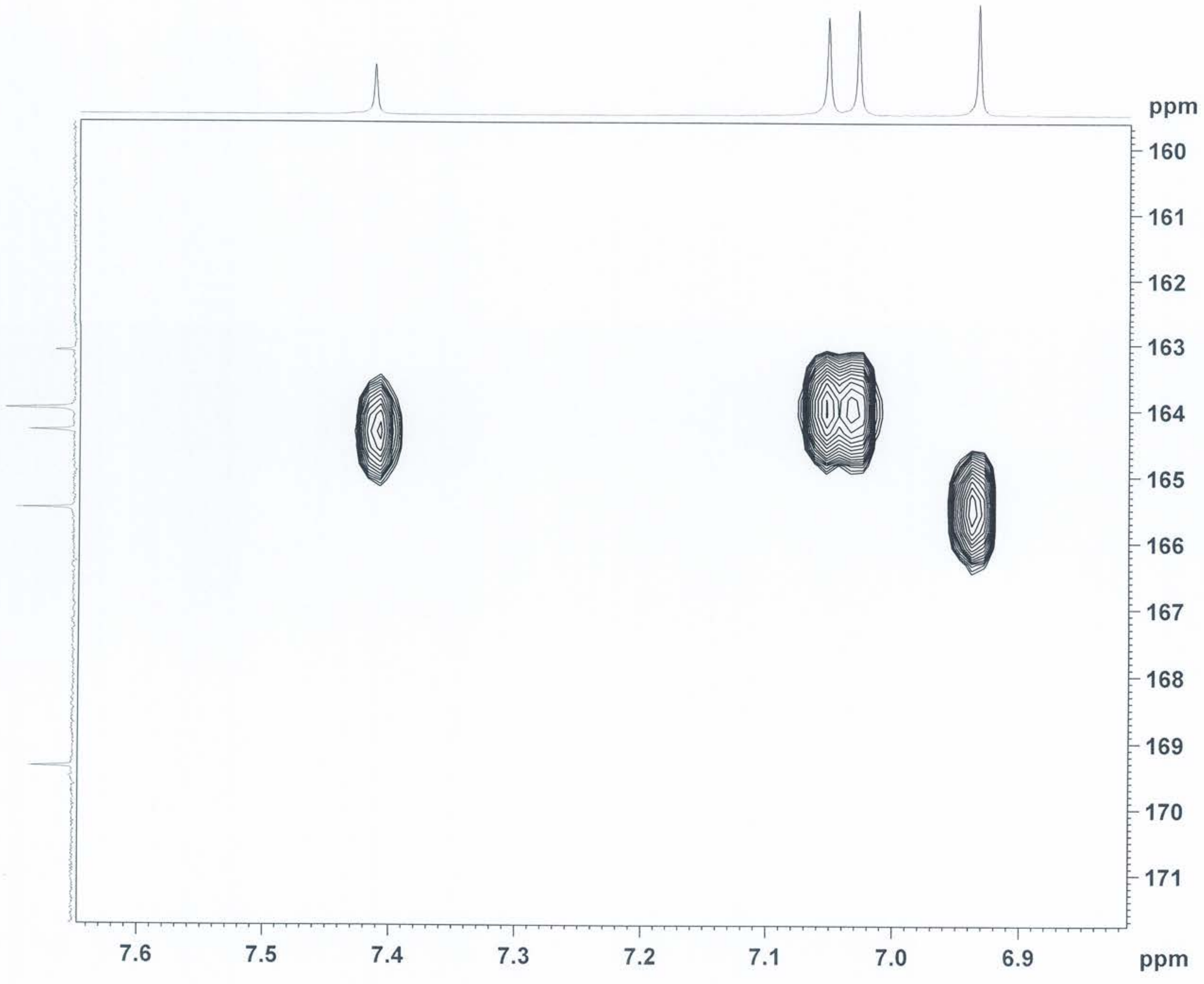

BRUKER

Current Data Parameters
NAME Component 5 of Chebulae Fructus_230401
EXPNO 6
PROCNO 1

F2 - Acquisition Parameters
Date_ 20231001
Time 16.17 h
INSTRUM Avance
PROBHD Z172446_0005 (
PULPROG hmbcetgpl3nd
TD 4096
SOLVENT DMSO
NS 64
DS 16
SWH 11904.762 Hz
FIDRES 5.812872 Hz
AQ 0.1720320 sec
RG 101
DW 42.000 usec
DE 6.50 usec
TE 298.2 K
CNST6 120.0000000
CNST7 170.0000000
CNST13 8.0000000
D0 0.00000300 sec
D1 2.00000000 sec
D6 0.06250000 sec
D16 0.00020000 sec
IN0 0.00001300 sec
TDav 1
SFO1 600.1336008 MHz
NUC1 1H
P1 11.92 usec
P2 23.84 usec
PLW1 17.17900085 W
SFO2 150.9178988 MHz
NUC2 13C
P3 11.80 usec
P24 2000.00 usec
PLW2 86.66300201 W
P16 1000.00 usec
CNST30 0.598116

F1 - Acquisition parameters
TD 256
SFO1 150.9179 MHz
FIDRES 300.625305 Hz
SW 254.973 ppm
FnMODE Echo-Antiecho

F2 - Processing parameters
SI 2048
SF 600.1300140 MHz
WDW SINE
SSB 4
LB 0 Hz
GB 0
PC 1.40

F1 - Processing parameters
SI 1024
MC2 echo-antiecho
SF 150.9028720 MHz
WDW QSINE
SSB 2
LB 0 Hz
GB 0

检测人: [illegible] 23.12.07
复核人: [illegible] 23.12.07

HP001

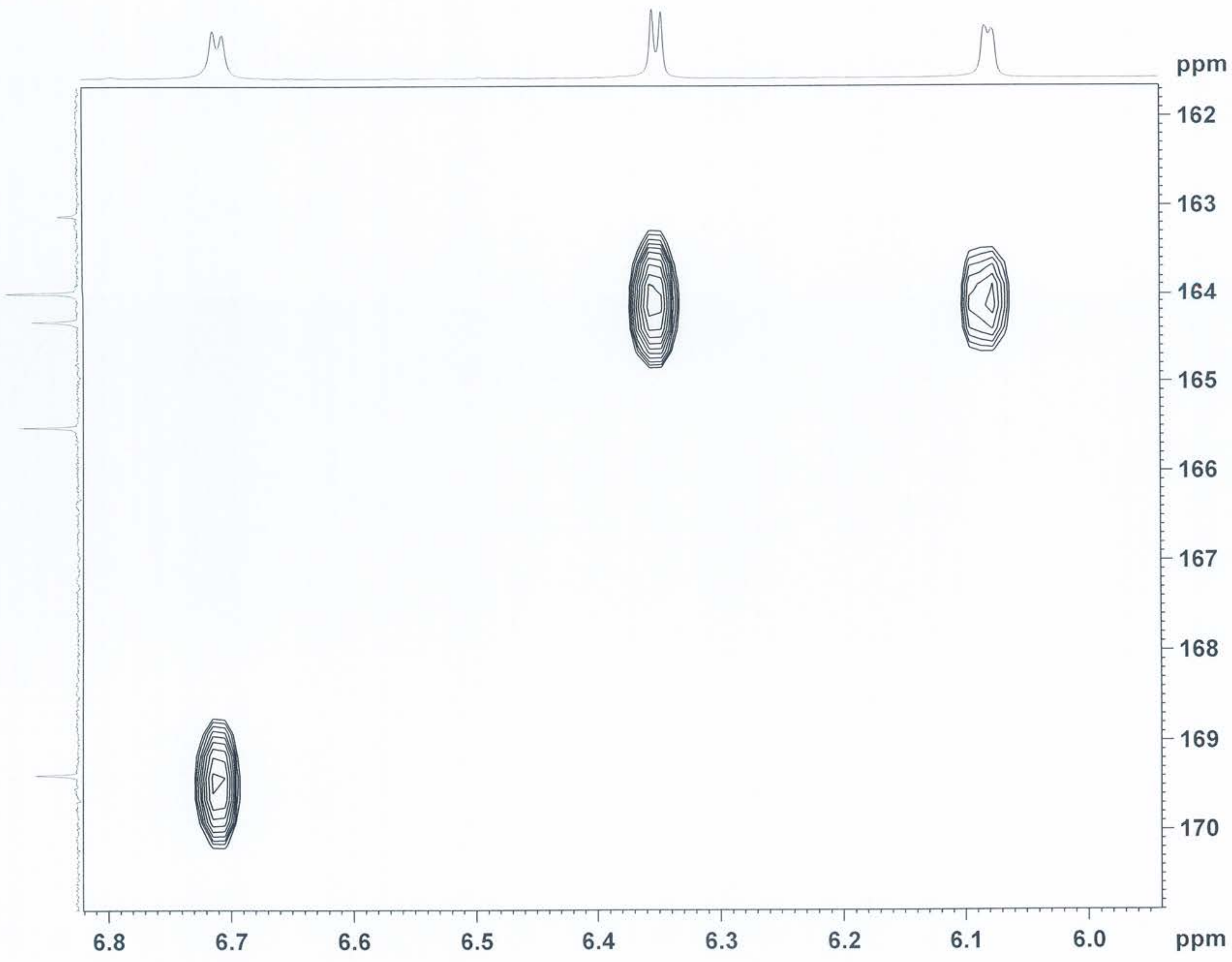

BRUKER

```
Current Data Parameters
NAME     Component 5 of
         Chebulae Fructus_230401
EXPNO              6
PROCNO             1

F2 - Acquisition Parameters
Date_          20231001
Time              16.17 h
INSTRUM          Avance
PROBHD    Z172446_0005 (
PULPROG     hmbcetgpl3nd
TD                4096
SOLVENT           DMSO
NS                  64
DS                  16
SWH          11904.762 Hz
FIDRES        5.812872 Hz
AQ           0.1720320 sec
RG                 101
DW              42.000 usec
DE                6.50 usec
TE               298.2 K
CNST6      120.0000000
CNST7      170.0000000
CNST13       8.0000000
D0          0.00000300 sec
D1          2.00000000 sec
D6          0.06250000 sec
D16         0.00020000 sec
IN0         0.00001300 sec
TDav                 1
SFO1       600.1336008 MHz
NUC1                1H
P1               11.92 usec
P2               23.84 usec
PLW1       17.17900085 W
SFO2       150.9178988 MHz
NUC2               13C
P3               11.80 usec
P24            2000.00 usec
PLW2       86.66300201 W
P16            1000.00 usec
CNST30        0.598116

F1 - Acquisition parameters
TD                 256
SFO1          150.9179 MHz
FIDRES      300.625305 Hz
SW             254.973 ppm
FnMODE    Echo-Antiecho

F2 - Processing parameters
SI                2048
SF         600.1300140 MHz
WDW               SINE
SSB                  4
LB               0 Hz
GB               0
PC                1.40

F1 - Processing parameters
SI                1024
MC2       echo-antiecho
SF         150.9028720 MHz
WDW              QSINE
SSB                  2
LB               0 Hz
GB               0
```

检测人：[illegible] 23.12.07

复核人：[illegible] 23.12.07

HP001

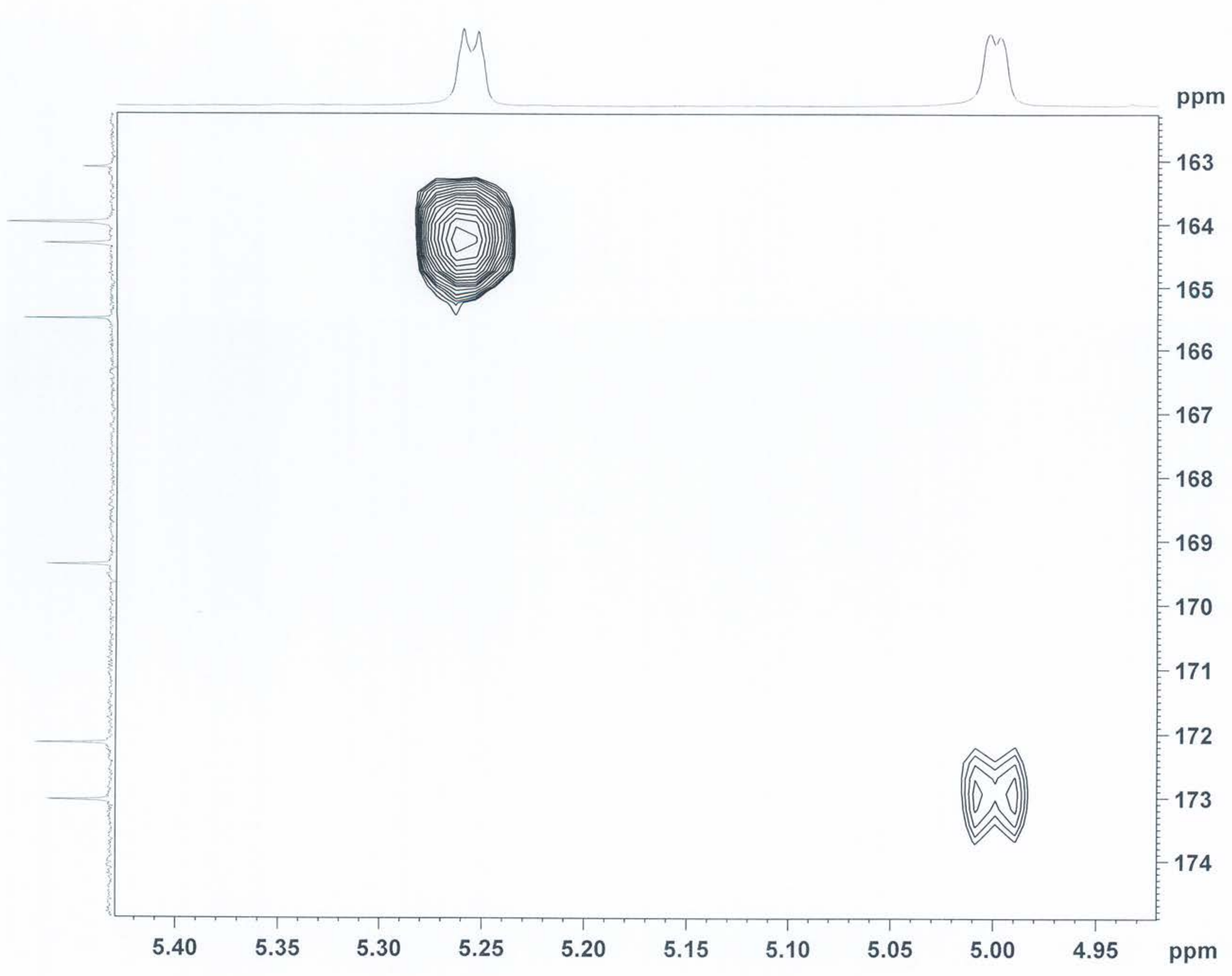

BRUKER

```
Current Data Parameters
NAME       Component 5 of
           Chebulae Fructus_230401
EXPNO                     6
PROCNO                    1

F2 - Acquisition Parameters
Date_              20231001
Time                  16.17 h
INSTRUM              Avance
PROBHD     Z172446_0005 (
PULPROG      hmbcetgpl3nd
TD                     4096
SOLVENT                DMSO
NS                       64
DS                       16
SWH               11904.762 Hz
FIDRES             5.812872 Hz
AQ                0.1720320 sec
RG                      101
DW                   42.000 usec
DE                     6.50 usec
TE                    298.2 K
CNST6           120.0000000
CNST7           170.0000000
CNST13            8.0000000
D0               0.00000300 sec
D1               2.00000000 sec
D6               0.06250000 sec
D16              0.00020000 sec
IN0              0.00001300 sec
TDav                      1
SFO1         600.1336008 MHz
NUC1                     1H
P1                    11.92 usec
P2                    23.84 usec
PLW1            17.17900085 W
SFO2         150.9178988 MHz
NUC2                    13C
P3                    11.80 usec
P24                 2000.00 usec
PLW2            86.66300201 W
P16                 1000.00 usec
CNST30             0.598116

F1 - Acquisition parameters
TD                      256
SFO1            150.9179 MHz
FIDRES        300.625305 Hz
SW               254.973 ppm
FnMODE        Echo-Antiecho

F2 - Processing parameters
SI                     2048
SF           600.1300140 MHz
WDW                    SINE
SSB                       4
LB             0 Hz
GB             0
PC                     1.40

F1 - Processing parameters
SI                     1024
MC2            echo-antiecho
SF           150.9028720 MHz
WDW                   QSINE
SSB                       2
LB             0 Hz
GB             0
```

检测人：[illegible] 23.12.27
复核人：[illegible] 23-12-27

HP001

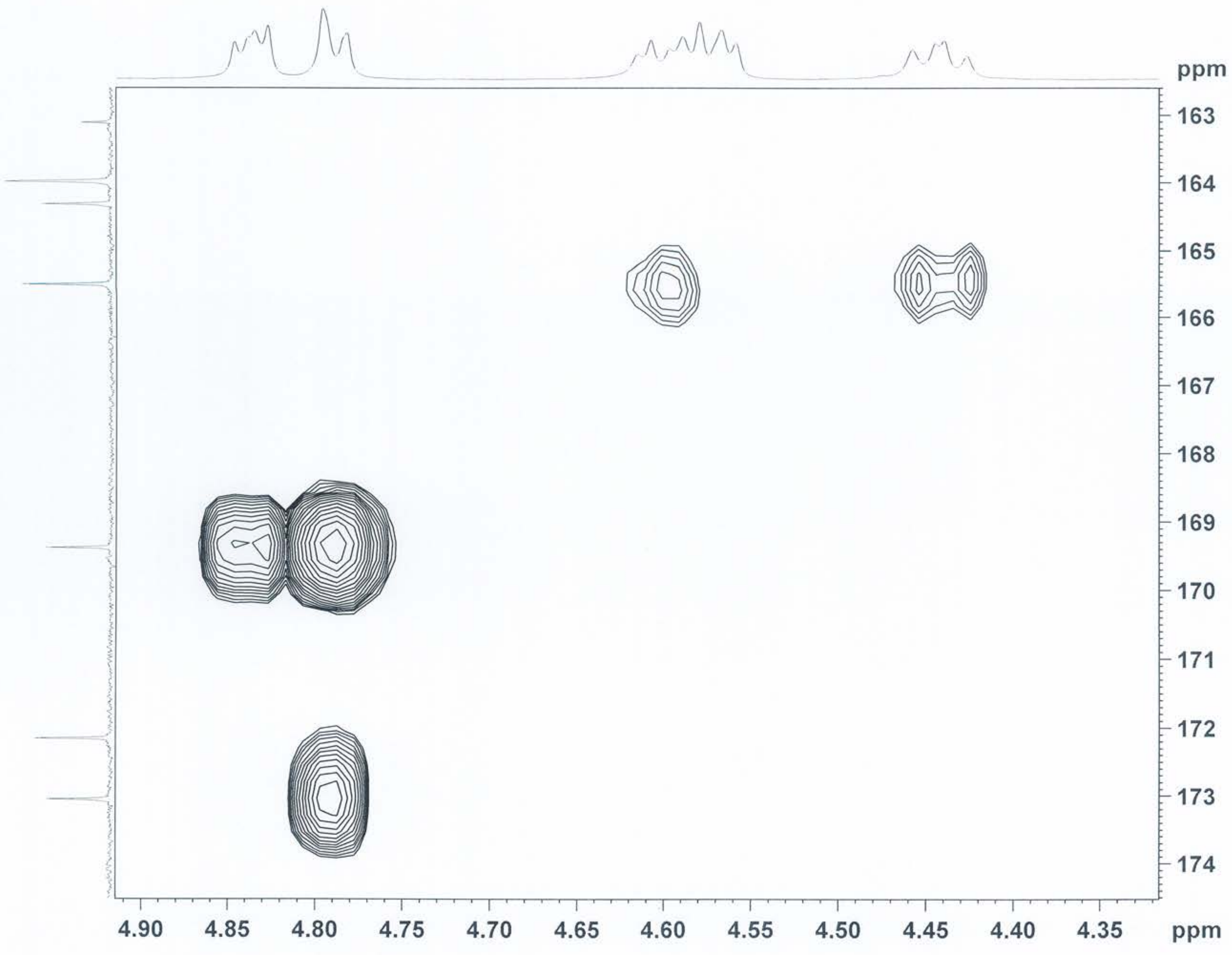

BRUKER

Current Data Parameters

NAME	Component 5 of Chebulae Fructus_230401
EXPNO	6
PROCNO	1

F2 - Acquisition Parameters

Date_	20231001
Time_	16.17 h
INSTRUM	Avance
PROBHD	Z172446_0005 (
PULPROG	hmbcetgpl3nd
TD	4096
SOLVENT	DMSO
NS	64
DS	16
SWH	11904.762 Hz
FIDRES	5.812872 Hz
AQ	0.1720320 sec
RG	101
DW	42.000 usec
DE	6.50 usec
TE	298.2 K
CNST6	120.0000000
CNST7	170.0000000
CNST13	8.0000000
D0	0.00000300 sec
D1	2.00000000 sec
D6	0.06250000 sec
D16	0.00020000 sec
IN0	0.00001300 sec
TDav	1
SFO1	600.1336008 MHz
NUC1	1H
P1	11.92 usec
P2	23.84 usec
PLW1	17.17900085 W
SFO2	150.9178988 MHz
NUC2	13C
P3	11.80 usec
P24	2000.00 usec
PLW2	86.66300201 W
P16	1000.00 usec
CNST30	0.598116

F1 - Acquisition parameters

TD	256
SFO1	150.9179 MHz
FIDRES	300.625305 Hz
SW	254.973 ppm
FnMODE	Echo-Antiecho

F2 - Processing parameters

SI	2048
SF	600.1300140 MHz
WDW	SINE
SSB	4
LB	0 Hz
GB	0
PC	1.40

F1 - Processing parameters

SI	1024
MC2	echo-antiecho
SF	150.9028720 MHz
WDW	QSINE
SSB	2
LB	0 Hz
GB	0

[illegible] 23.12.27
[illegible] 23.12.27

HP001

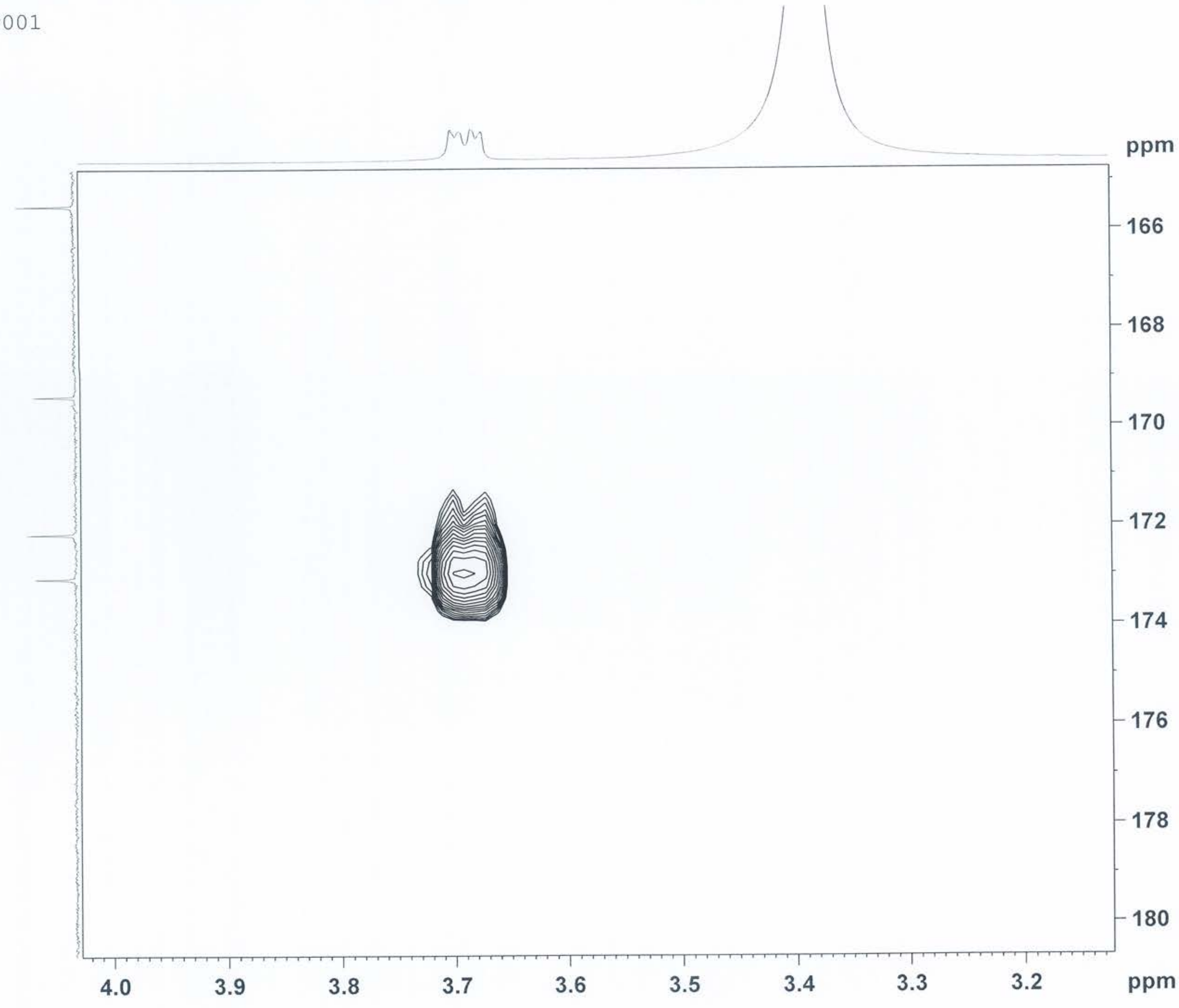

BRUKER

Current Data Parameters
NAME Component 5 of Chebulae Fructus_230401
EXPNO 6
PROCNO 1

F2 - Acquisition Parameters
Date_ 20231001
Time_ 16.17 h
INSTRUM Avance
PROBHD Z172446_0005 (
PULPROG hmbcetgpl3nd
TD 4096
SOLVENT DMSO
NS 64
DS 16
SWH 11904.762 Hz
FIDRES 5.812872 Hz
AQ 0.1720320 sec
RG 101
DW 42.000 usec
DE 6.50 usec
TE 298.2 K
CNST6 120.0000000
CNST7 170.0000000
CNST13 8.0000000
D0 0.00000300 sec
D1 2.00000000 sec
D6 0.06250000 sec
D16 0.00020000 sec
IN0 0.00001300 sec
TDav 1
SFO1 600.1336008 MHz
NUC1 1H
P1 11.92 usec
P2 23.84 usec
PLW1 17.17900085 W
SFO2 150.9178988 MHz
NUC2 13C
P3 11.80 usec
P24 2000.00 usec
PLW2 86.66300201 W
P16 1000.00 usec
CNST30 0.598116

F1 - Acquisition parameters
TD 256
SFO1 150.9179 MHz
FIDRES 300.625305 Hz
SW 254.973 ppm
FnMODE Echo-Antiecho

F2 - Processing parameters
SI 2048
SF 600.1300140 MHz
WDW SINE
SSB 4
LB 0 Hz
GB 0
PC 1.40

F1 - Processing parameters
SI 1024
MC2 echo-antiecho
SF 150.9028720 MHz
WDW QSINE
SSB 2
LB 0 Hz
GB 0

检测人: [illegible] 23.12.07
复核人: [illegible] 23.12.07

HP001

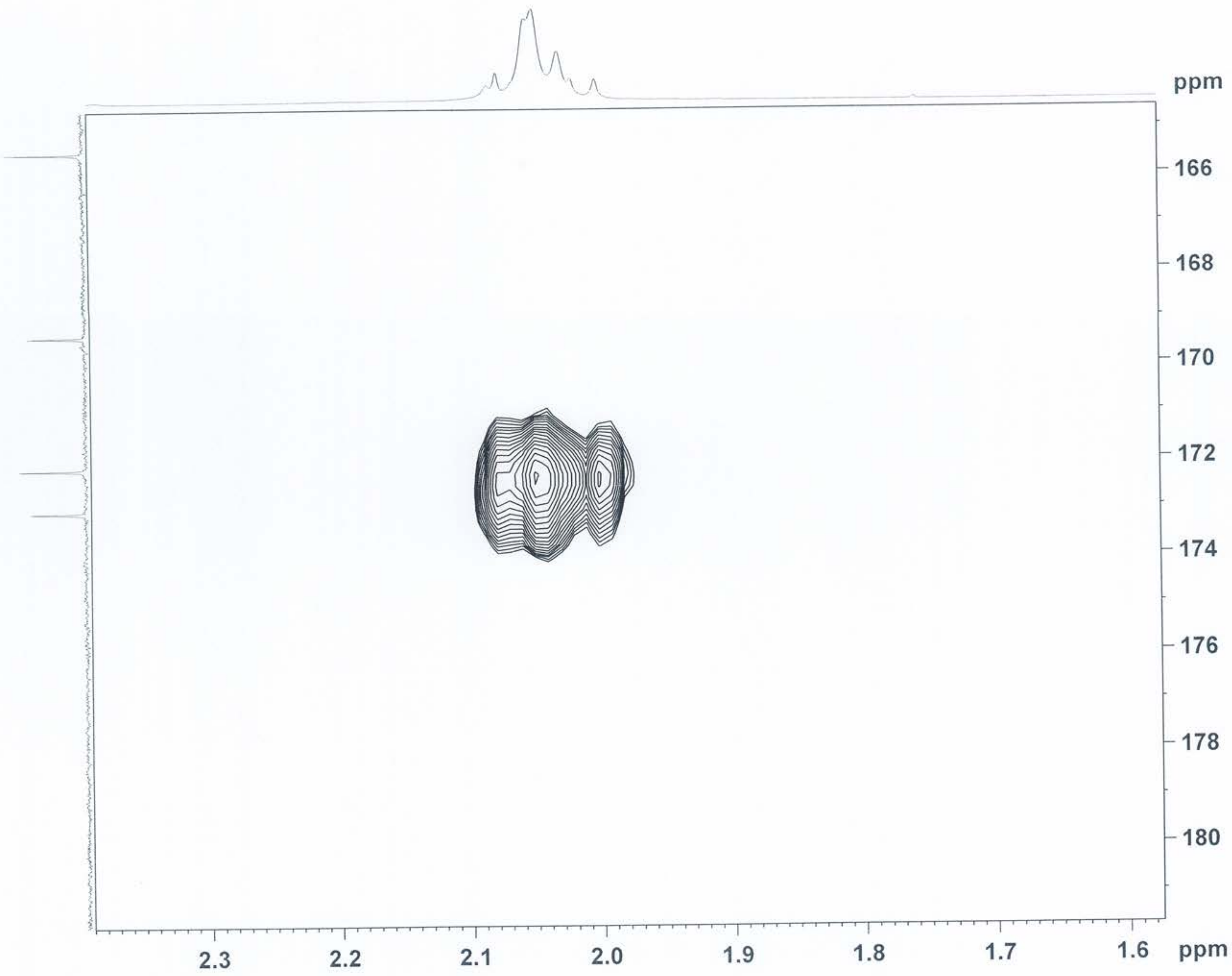


```
Current Data Parameters
NAME        Component 5 of
            Chebulae Fructus_230401
EXPNO                    6
PROCNO                   1

F2 - Acquisition Parameters
Date_             20231001
Time                 16.17 h
INSTRUM             Avance
PROBHD     Z172446_0005 (
PULPROG      hmbcetgpl3nd
TD                    4096
SOLVENT               DMSO
NS                      64
DS                      16
SWH              11904.762 Hz
FIDRES            5.812872 Hz
AQ               0.1720320 sec
RG                     101
DW                  42.000 usec
DE                    6.50 usec
TE                   298.2 K
CNST6          120.0000000
CNST7          170.0000000
CNST13           8.0000000
D0              0.00000300 sec
D1              2.00000000 sec
D6              0.06250000 sec
D16             0.00020000 sec
IN0             0.00001300 sec
TDav                     1
SFO1          600.1336008 MHz
NUC1                    1H
P1                   11.92 usec
P2                   23.84 usec
PLW1           17.17900085 W
SFO2          150.9178988 MHz
NUC2                   13C
P3                   11.80 usec
P24                2000.00 usec
PLW2           86.66300201 W
P16                1000.00 usec
CNST30            0.598116

F1 - Acquisition parameters
TD                     256
SFO1              150.9179 MHz
FIDRES          300.625305 Hz
SW                 254.973 ppm
FnMODE        Echo-Antiecho

F2 - Processing parameters
SI                    2048
SF             600.1300140 MHz
WDW                   SINE
SSB                      4
LB                0 Hz
GB                0
PC                    1.40

F1 - Processing parameters
SI                    1024
MC2          echo-antiecho
SF             150.9028720 MHz
WDW                  QSINE
SSB                      2
LB                0 Hz
GB                0
```

核对人：[illegible] 23.12.07
复校人：[illegible] 23.12.07

HP001

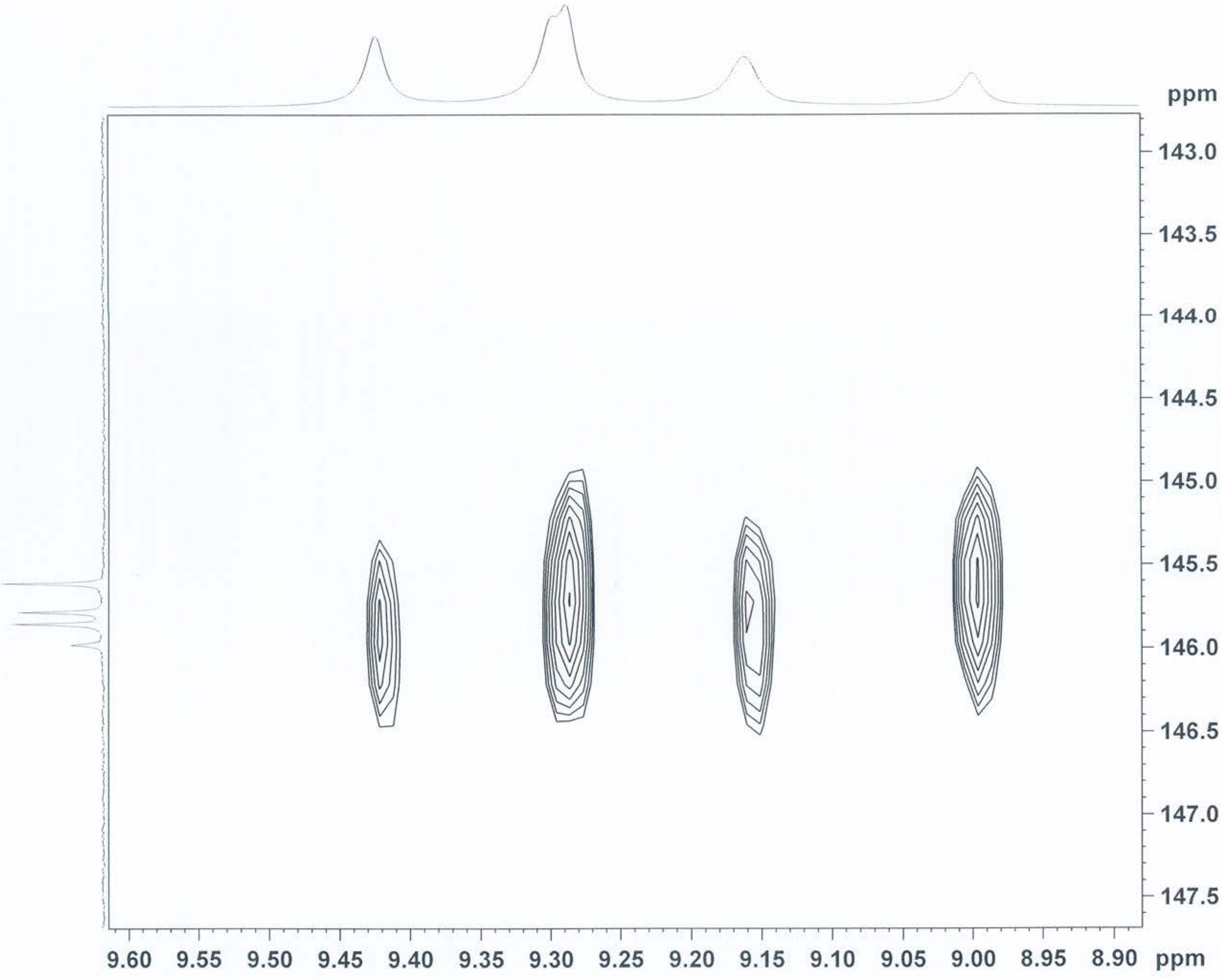

BRUKER

```
Current Data Parameters
NAME     Component 5 of
         Chebulae Fructus_230401
EXPNO                    6
PROCNO                   1

F2 - Acquisition Parameters
Date_          20231001
Time              16.17 h
INSTRUM          Avance
PROBHD   Z172446_0005 (
PULPROG    hmbcetgpl3nd
TD                 4096
SOLVENT            DMSO
NS                   64
DS                   16
SWH           11904.762 Hz
FIDRES         5.812872 Hz
AQ            0.1720320 sec
RG                  101
DW               42.000 usec
DE                 6.50 usec
TE                298.2 K
CNST6       120.0000000
CNST7       170.0000000
CNST13        8.0000000
D0          0.00000300 sec
D1          2.00000000 sec
D6          0.06250000 sec
D16         0.00020000 sec
IN0         0.00001300 sec
TDav                  1
SFO1        600.1336008 MHz
NUC1                 1H
P1                11.92 usec
P2                23.84 usec
PLW1        17.17900085 W
SFO2        150.9178988 MHz
NUC2                13C
P3                11.80 usec
P24             2000.00 usec
PLW2        86.66300201 W
P16             1000.00 usec
CNST30         0.598116

F1 - Acquisition parameters
TD                  256
SFO1           150.9179 MHz
FIDRES       300.625305 Hz
SW              254.973 ppm
FnMODE     Echo-Antiecho

F2 - Processing parameters
SI                 2048
SF          600.1300140 MHz
WDW                SINE
SSB                   4
LB                 0 Hz
GB                 0
PC                 1.40

F1 - Processing parameters
SI                 1024
MC2        echo-antiecho
SF          150.9028720 MHz
WDW               QSINE
SSB                   2
LB                 0 Hz
GB                 0
```

检测人：[illegible] 23. 12.07
审核人：[illegible] 23.12.07

HP001

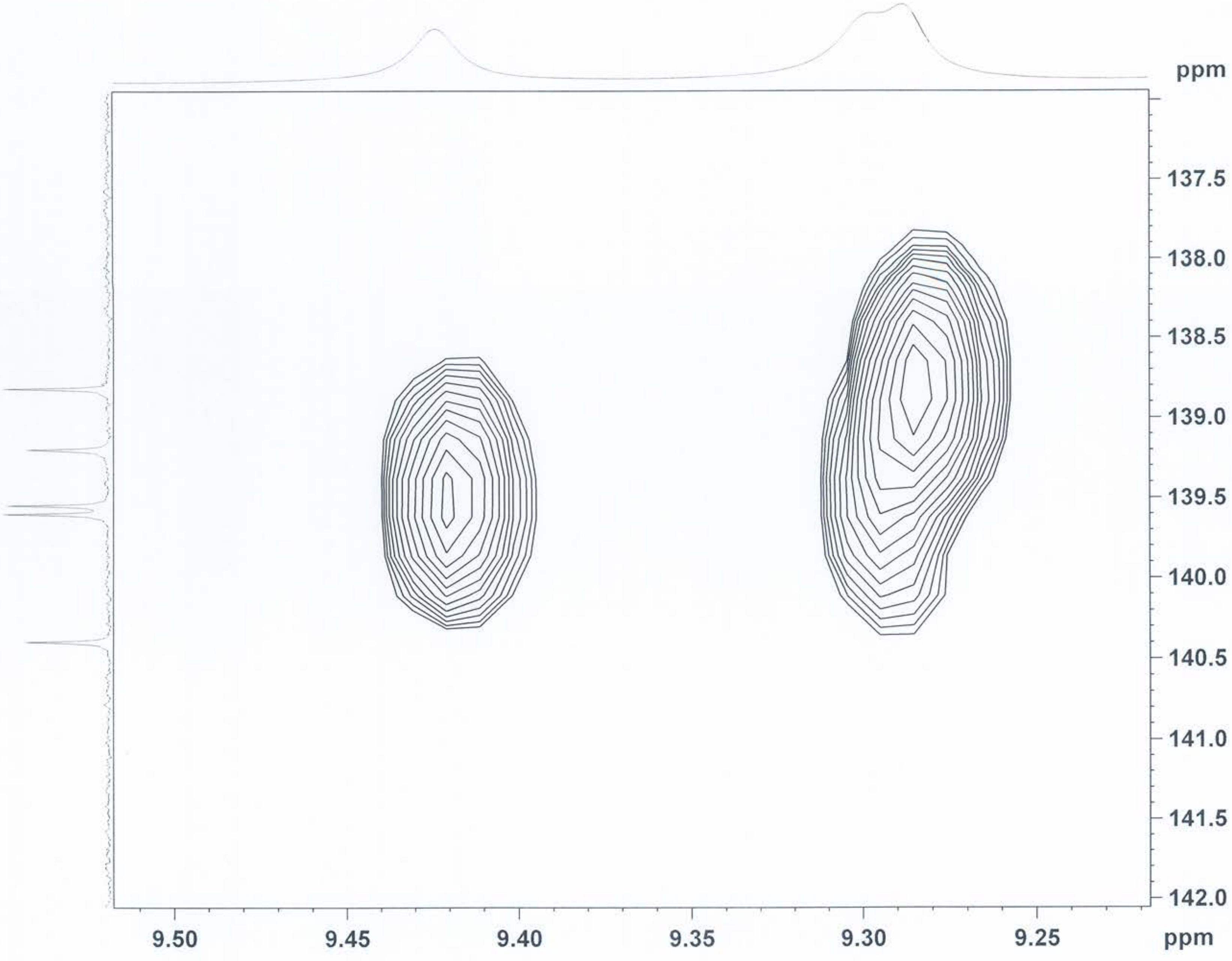

BRUKER

```
Current Data Parameters
NAME      Component 5 of
          Chebulae Fructus_230401
EXPNO                    6
PROCNO                   1

F2 - Acquisition Parameters
Date_             20231001
Time                 16.17 h
INSTRUM             Avance
PROBHD   Z172446_0005 (
PULPROG     hmbcetgpl3nd
TD                    4096
SOLVENT               DMSO
NS                      64
DS                      16
SWH              11904.762 Hz
FIDRES            5.812872 Hz
AQ               0.1720320 sec
RG                     101
DW                  42.000 usec
DE                    6.50 usec
TE                   298.2 K
CNST6          120.0000000
CNST7          170.0000000
CNST13           8.0000000
D0              0.00000300 sec
D1              2.00000000 sec
D6              0.06250000 sec
D16             0.00020000 sec
IN0             0.00001300 sec
TDav                     1
SFO1           600.1336008 MHz
NUC1                    1H
P1                   11.92 usec
P2                   23.84 usec
PLW1           17.17900085 W
SFO2           150.9178988 MHz
NUC2                   13C
P3                   11.80 usec
P24                2000.00 usec
PLW2           86.66300201 W
P16                1000.00 usec
CNST30            0.598116

F1 - Acquisition parameters
TD                     256
SFO1              150.9179 MHz
FIDRES          300.625305 Hz
SW                 254.973 ppm
FnMODE        Echo-Antiecho

F2 - Processing parameters
SI                    2048
SF             600.1300140 MHz
WDW                   SINE
SSB                      4
LB                    0 Hz
GB                    0
PC                    1.40

F1 - Processing parameters
SI                    1024
MC2          echo-antiecho
SF             150.9028720 MHz
WDW                  QSINE
SSB                      2
LB                    0 Hz
GB                    0
```

[illegible] 23.12.07

复核人：[illegible] 23.12.07

HP001

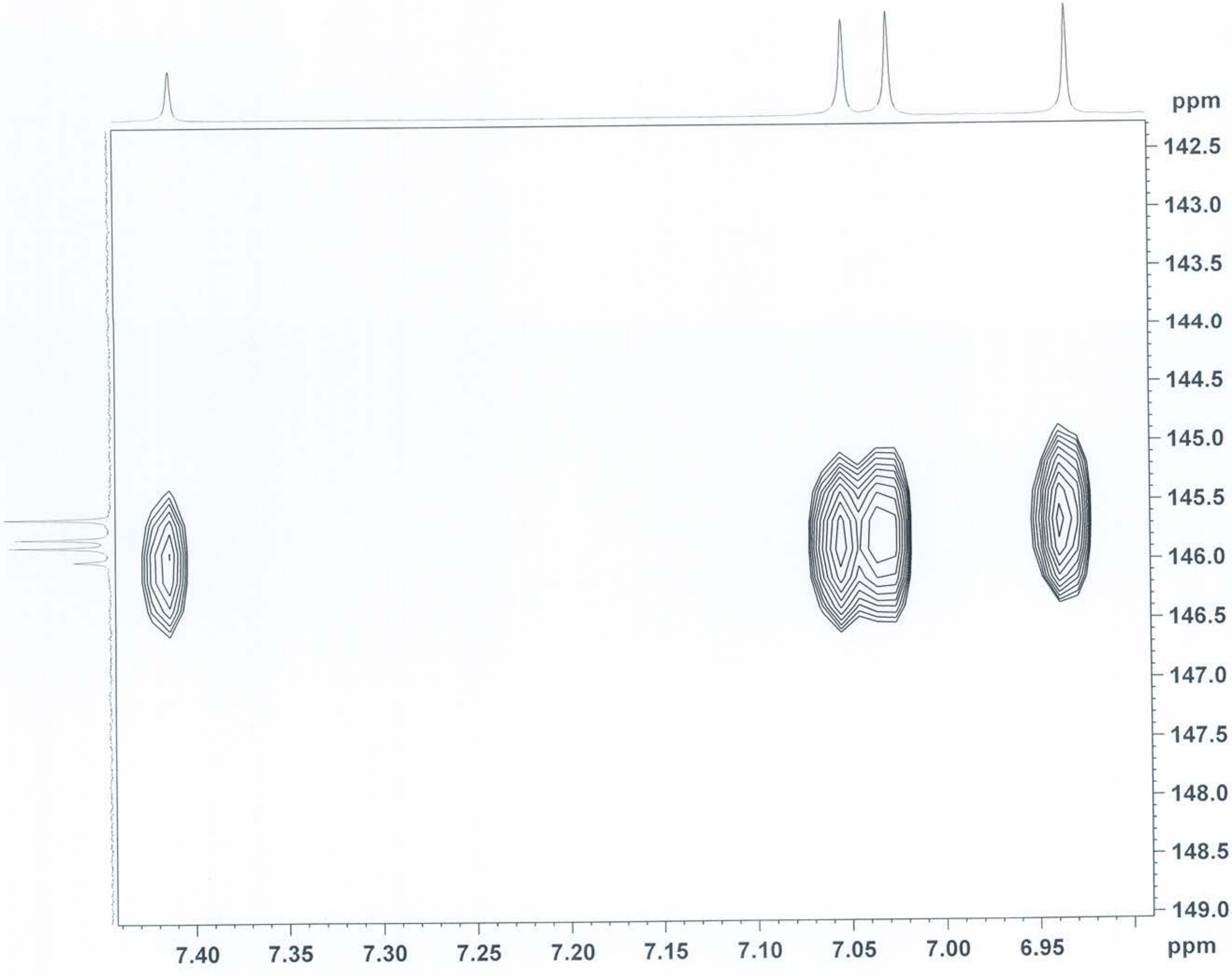

BRUKER

Current Data Parameters

NAME	Component 5 of Chebulae Fructus_230401
EXPNO	6
PROCNO	1

F2 - Acquisition Parameters

Date_	20231001
Time	16.17 h
INSTRUM	Avance
PROBHD	Z172446_0005 (
PULPROG	hmbcetgpl3nd
TD	4096
SOLVENT	DMSO
NS	64
DS	16
SWH	11904.762 Hz
FIDRES	5.812872 Hz
AQ	0.1720320 sec
RG	101
DW	42.000 usec
DE	6.50 usec
TE	298.2 K
CNST6	120.0000000
CNST7	170.0000000
CNST13	8.0000000
D0	0.00000300 sec
D1	2.00000000 sec
D6	0.06250000 sec
D16	0.00020000 sec
IN0	0.00001300 sec
TDav	1
SFO1	600.1336008 MHz
NUC1	1H
P1	11.92 usec
P2	23.84 usec
PLW1	17.17900085 W
SFO2	150.9178988 MHz
NUC2	13C
P3	11.80 usec
P24	2000.00 usec
PLW2	86.66300201 W
P16	1000.00 usec
CNST30	0.598116

F1 - Acquisition parameters

TD	256
SFO1	150.9179 MHz
FIDRES	300.625305 Hz
SW	254.973 ppm
FnMODE	Echo-Antiecho

F2 - Processing parameters

SI	2048
SF	600.1300140 MHz
WDW	SINE
SSB	4
LB	0 Hz
GB	0
PC	1.40

F1 - Processing parameters

SI	1024
MC2	echo-antiecho
SF	150.9028720 MHz
WDW	QSINE
SSB	2
LB	0 Hz
GB	0

[illegible] 23.12.27
[illegible] 23.12.27

HP001

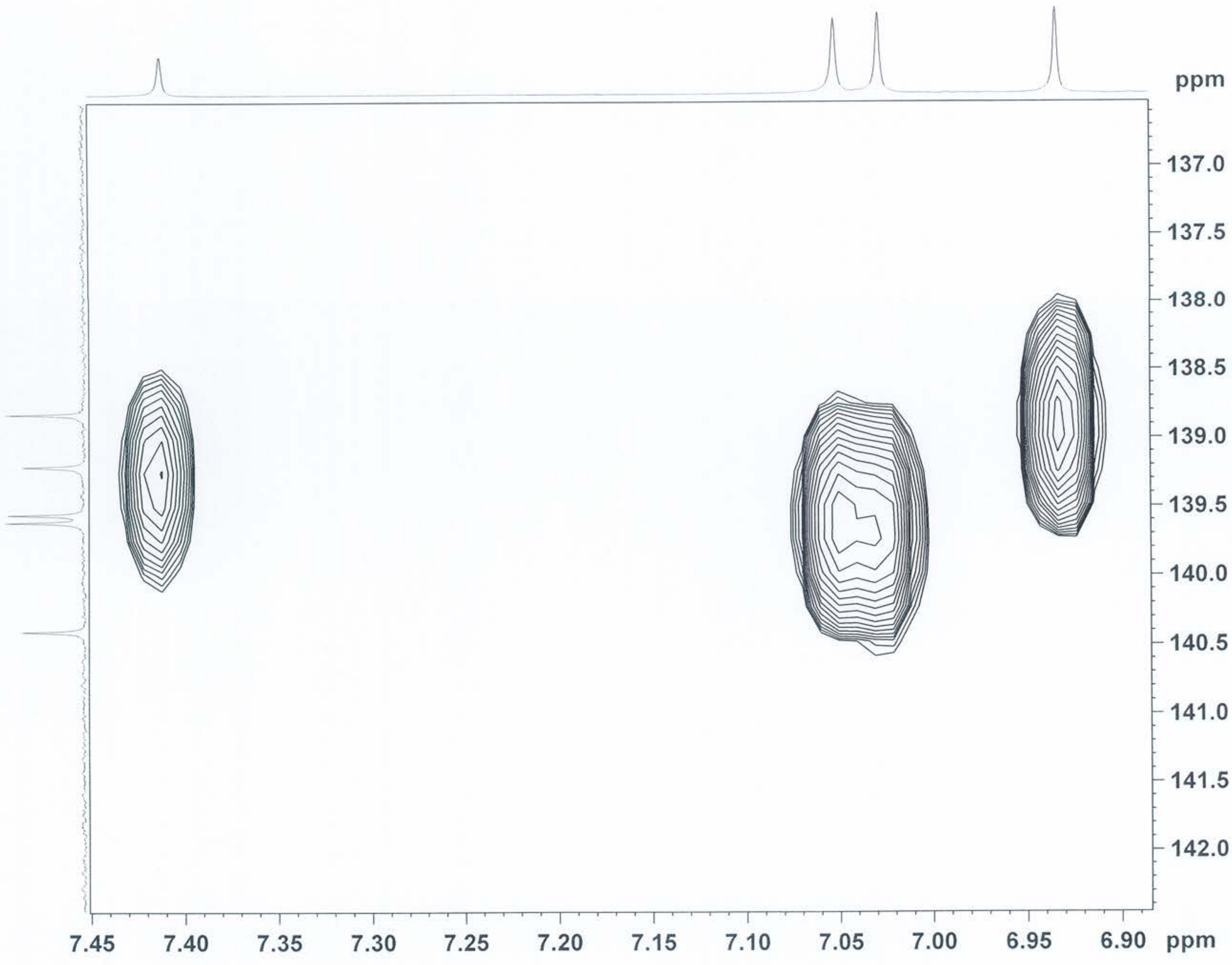

Current Data Parameters
NAME Component 5 of Chebulae Fructus_230401
EXPNO 6
PROCNO 1

F2 - Acquisition Parameters

Date_	20231001
Time	16.17 h
INSTRUM	Avance
PROBHD	Z172446_0005 (
PULPROG	hmbcetgpl3nd
TD	4096
SOLVENT	DMSO
NS	64
DS	16
SWH	11904.762 Hz
FIDRES	5.812872 Hz
AQ	0.1720320 sec
RG	101
DW	42.000 usec
DE	6.50 usec
TE	298.2 K
CNST6	120.0000000
CNST7	170.0000000
CNST13	8.0000000
D0	0.00000300 sec
D1	2.00000000 sec
D6	0.06250000 sec
D16	0.00020000 sec
IN0	0.00001300 sec
TDav	1
SFO1	600.1336008 MHz
NUC1	1H
P1	11.92 usec
P2	23.84 usec
PLW1	17.17900085 W
SFO2	150.9178988 MHz
NUC2	13C
P3	11.80 usec
P24	2000.00 usec
PLW2	86.66300201 W
P16	1000.00 usec
CNST30	0.598116

F1 - Acquisition parameters

TD	256
SFO1	150.9179 MHz
FIDRES	300.625305 Hz
SW	254.973 ppm
FnMODE	Echo-Antiecho

F2 - Processing parameters

SI	2048
SF	600.1300140 MHz
WDW	SINE
SSB	4
LB	0 Hz
GB	0
PC	1.40

F1 - Processing parameters

SI	1024
MC2	echo-antiecho
SF	150.9028720 MHz
WDW	QSINE
SSB	2
LB	0 Hz
GB	0

检测人: [illegible] 23.12.07
复核人: [illegible] 23.12.07

HP001

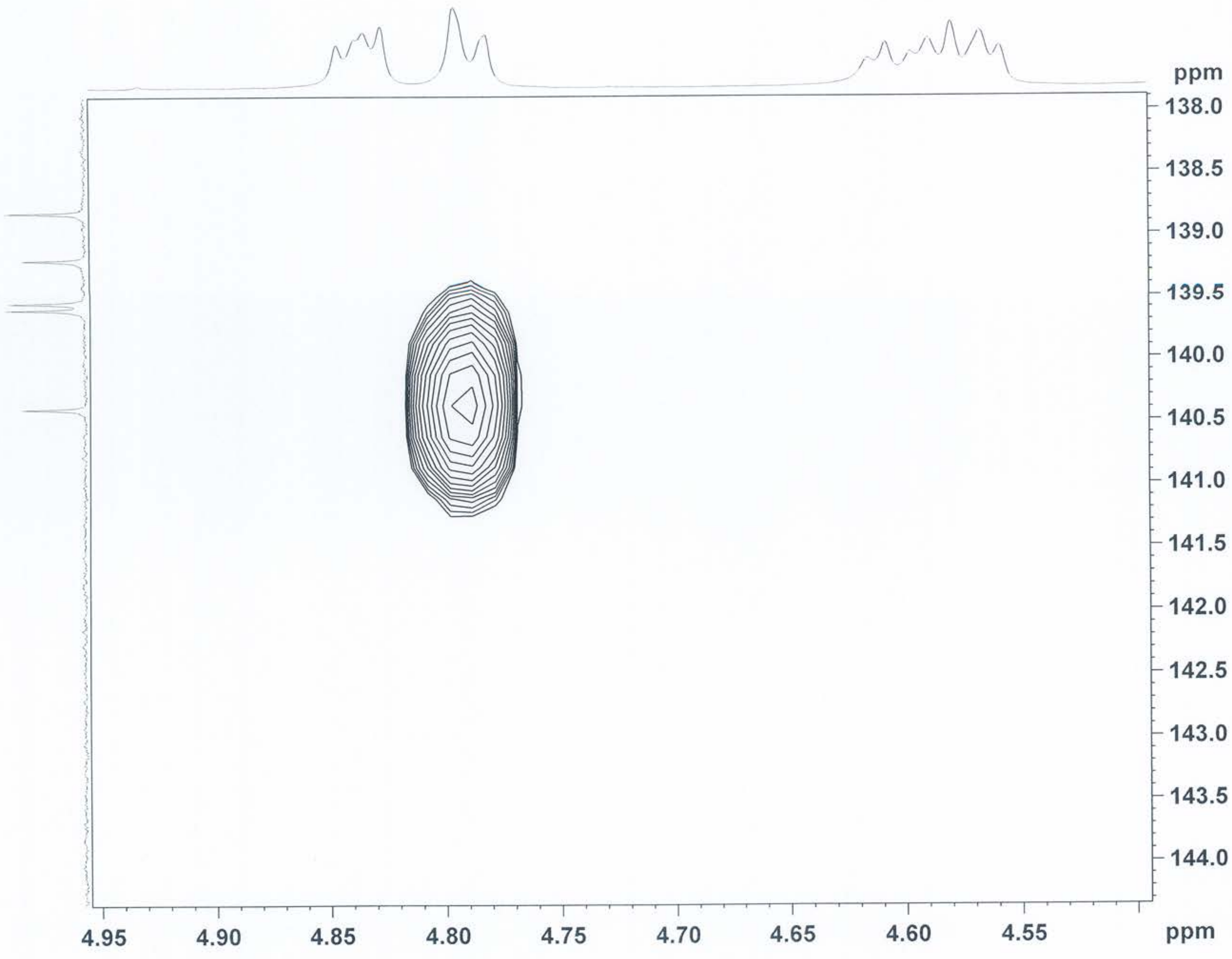

BRUKER

```
Current Data Parameters
NAME      Component 5 of
          Chebulae Fructus_230401
EXPNO                   6
PROCNO                  1

F2 - Acquisition Parameters
Date_            20231001
Time              16.17 h
INSTRUM            Avance
PROBHD     Z172446_0005 (
PULPROG      hmbcetgpl3nd
TD                   4096
SOLVENT              DMSO
NS                     64
DS                     16
SWH             11904.762 Hz
FIDRES           5.812872 Hz
AQ              0.1720320 sec
RG                    101
DW                 42.000 usec
DE                   6.50 usec
TE                  298.2 K
CNST6         120.0000000
CNST7         170.0000000
CNST13          8.0000000
D0             0.00000300 sec
D1             2.00000000 sec
D6             0.06250000 sec
D16            0.00020000 sec
IN0            0.00001300 sec
TDav                    1
SFO1          600.1336008 MHz
NUC1                   1H
P1                  11.92 usec
P2                  23.84 usec
PLW1          17.17900085 W
SFO2          150.9178988 MHz
NUC2                  13C
P3                  11.80 usec
P24               2000.00 usec
PLW2          86.66300201 W
P16               1000.00 usec
CNST30           0.598116

F1 - Acquisition parameters
TD                    256
SFO1             150.9179 MHz
FIDRES         300.625305 Hz
SW                254.973 ppm
FnMODE       Echo-Antiecho

F2 - Processing parameters
SI                   2048
SF            600.1300140 MHz
WDW                  SINE
SSB                     4
LB             0 Hz
GB             0
PC                   1.40

F1 - Processing parameters
SI                   1024
MC2          echo-antiecho
SF            150.9028720 MHz
WDW                 QSINE
SSB                     2
LB             0 Hz
GB             0
```

检测人：[illegible] 23.12.27
复核人：[illegible] 23.12.27

HP001

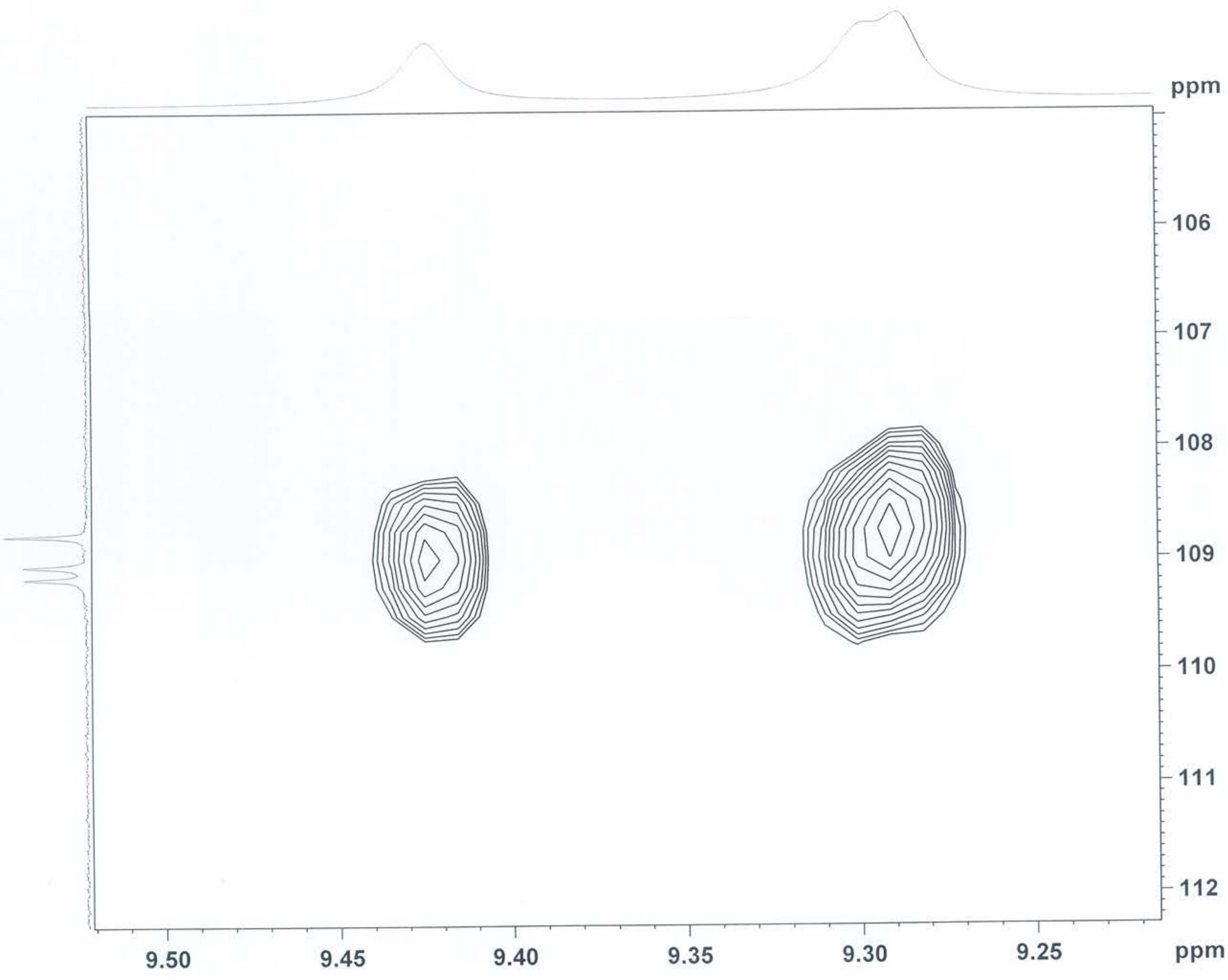

BRUKER

```
Current Data Parameters
NAME      Component 5 of
          Chebulae Fructus_230401
EXPNO                  6
PROCNO                 1

F2 - Acquisition Parameters
Date_           20231001
Time               16.17 h
INSTRUM           Avance
PROBHD    Z172446_0005 (
PULPROG     hmbcetgpl3nd
TD                  4096
SOLVENT             DMSO
NS                    64
DS                    16
SWH            11904.762 Hz
FIDRES          5.812872 Hz
AQ             0.1720320 sec
RG                   101
DW                42.000 usec
DE                  6.50 usec
TE                 298.2 K
CNST6        120.0000000
CNST7        170.0000000
CNST13         8.0000000
D0            0.00000300 sec
D1            2.00000000 sec
D6            0.06250000 sec
D16           0.00020000 sec
IN0           0.00001300 sec
TDav                   1
SFO1        600.1336008 MHz
NUC1                  1H
P1                 11.92 usec
P2                 23.84 usec
PLW1         17.17900085 W
SFO2         150.9178988 MHz
NUC2                 13C
P3                 11.80 usec
P24              2000.00 usec
PLW2         86.66300201 W
P16              1000.00 usec
CNST30          0.598116

F1 - Acquisition parameters
TD                   256
SFO1            150.9179 MHz
FIDRES        300.625305 Hz
SW               254.973 ppm
FnMODE      Echo-Antiecho

F2 - Processing parameters
SI                  2048
SF           600.1300140 MHz
WDW                 SINE
SSB                    4
LB            0 Hz
GB            0
PC                  1.40

F1 - Processing parameters
SI                  1024
MC2          echo-antiecho
SF           150.9028720 MHz
WDW                QSINE
SSB                    2
LB            0 Hz
GB            0
```

检测人：[illegible] 23.12.07

复核人：[illegible] 23.12.07

HP001

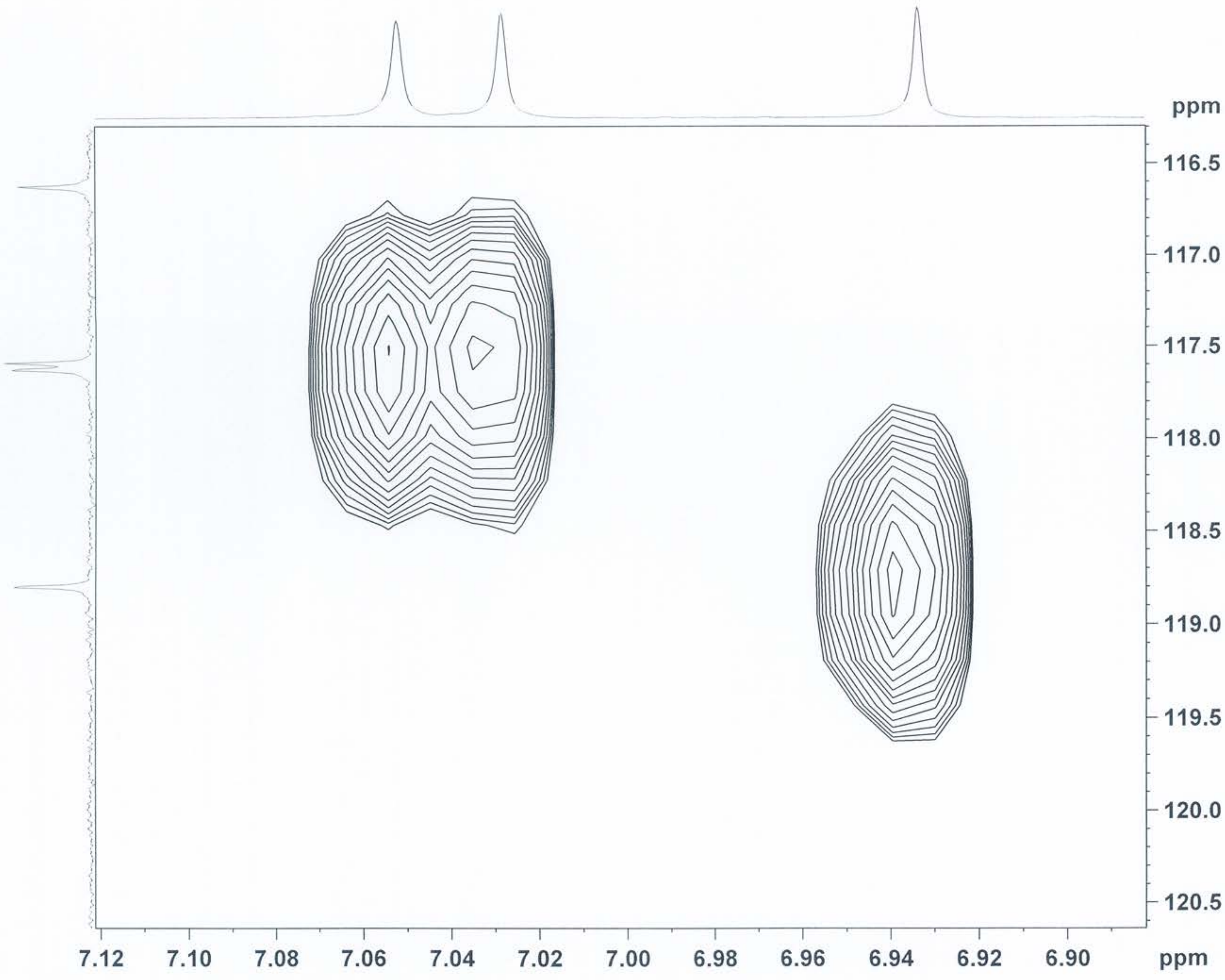

BRUKER

```
Current Data Parameters
NAME      Component 5 of
          Chebulae Fructus_230401
EXPNO                  6
PROCNO                 1

F2 - Acquisition Parameters
Date_           20231001
Time               16.17 h
INSTRUM           Avance
PROBHD   Z172446_0005 (
PULPROG    hmbcetgpl3nd
TD                  4096
SOLVENT             DMSO
NS                    64
DS                    16
SWH            11904.762 Hz
FIDRES          5.812872 Hz
AQ             0.1720320 sec
RG                   101
DW                42.000 usec
DE                  6.50 usec
TE                 298.2 K
CNST6        120.0000000
CNST7        170.0000000
CNST13         8.0000000
D0            0.00000300 sec
D1            2.00000000 sec
D6            0.06250000 sec
D16           0.00020000 sec
IN0           0.00001300 sec
TDav                   1
SFO1         600.1336008 MHz
NUC1                  1H
P1                 11.92 usec
P2                 23.84 usec
PLW1         17.17900085 W
SFO2         150.9178988 MHz
NUC2                 13C
P3                 11.80 usec
P24              2000.00 usec
PLW2         86.66300201 W
P16              1000.00 usec
CNST30          0.598116

F1 - Acquisition parameters
TD                   256
SFO1            150.9179 MHz
FIDRES        300.625305 Hz
SW               254.973 ppm
FnMODE      Echo-Antiecho

F2 - Processing parameters
SI                  2048
SF           600.1300140 MHz
WDW                 SINE
SSB                    4
LB             0 Hz
GB             0
PC                  1.40

F1 - Processing parameters
SI                  1024
MC2          echo-antiecho
SF           150.9028720 MHz
WDW                QSINE
SSB                    2
LB             0 Hz
GB             0
```

检测人: [illegible] 23.12.07
复核人: [illegible]

HP001

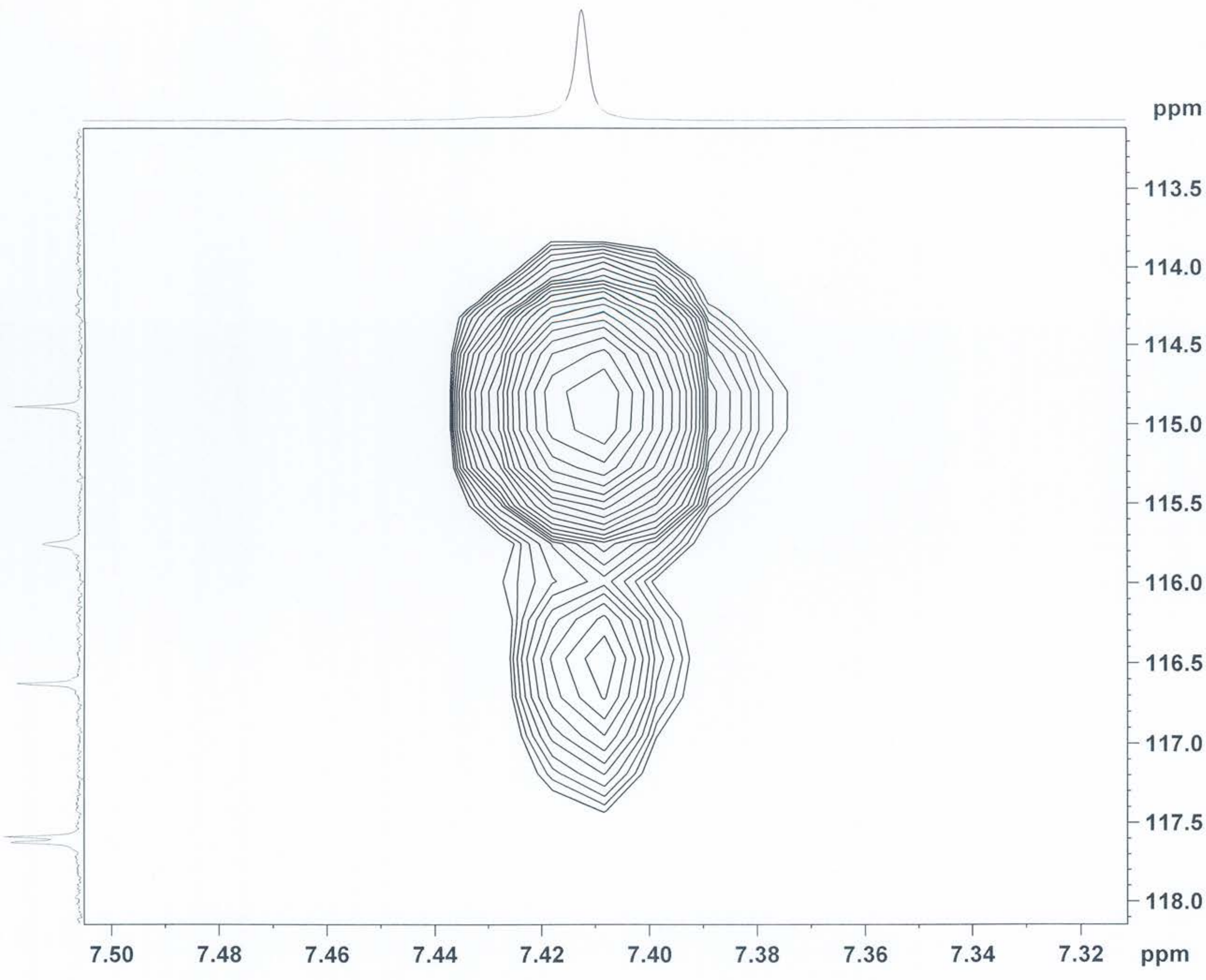

BRUKER

```
Current Data Parameters
NAME     Component 5 of
         Chebulae Fructus_230401
EXPNO                6
PROCNO               1

F2 - Acquisition Parameters
Date_          20231001
Time              16.17 h
INSTRUM          Avance
PROBHD   Z172446_0005 (
PULPROG   hmbcetgpl3nd
TD                4096
SOLVENT           DMSO
NS                  64
DS                  16
SWH          11904.762 Hz
FIDRES        5.812872 Hz
AQ           0.1720320 sec
RG                 101
DW              42.000 usec
DE                6.50 usec
TE               298.2 K
CNST6      120.0000000
CNST7      170.0000000
CNST13       8.0000000
D0          0.00000300 sec
D1          2.00000000 sec
D6          0.06250000 sec
D16         0.00020000 sec
IN0         0.00001300 sec
TDav                 1
SFO1       600.1336008 MHz
NUC1                1H
P1               11.92 usec
P2               23.84 usec
PLW1       17.17900085 W
SFO2       150.9178988 MHz
NUC2               13C
P3               11.80 usec
P24            2000.00 usec
PLW2       86.66300201 W
P16            1000.00 usec
CNST30        0.598116

F1 - Acquisition parameters
TD                 256
SFO1          150.9179 MHz
FIDRES      300.625305 Hz
SW             254.973 ppm
FnMODE    Echo-Antiecho

F2 - Processing parameters
SI                2048
SF         600.1300140 MHz
WDW               SINE
SSB                  4
LB        0 Hz
GB        0
PC                1.40

F1 - Processing parameters
SI                1024
MC2       echo-antiecho
SF         150.9028720 MHz
WDW              QSINE
SSB                  2
LB        0 Hz
GB        0
```

校对人：[illegible] 23.12.07
复核人：[illegible] 23.12.07

HP001

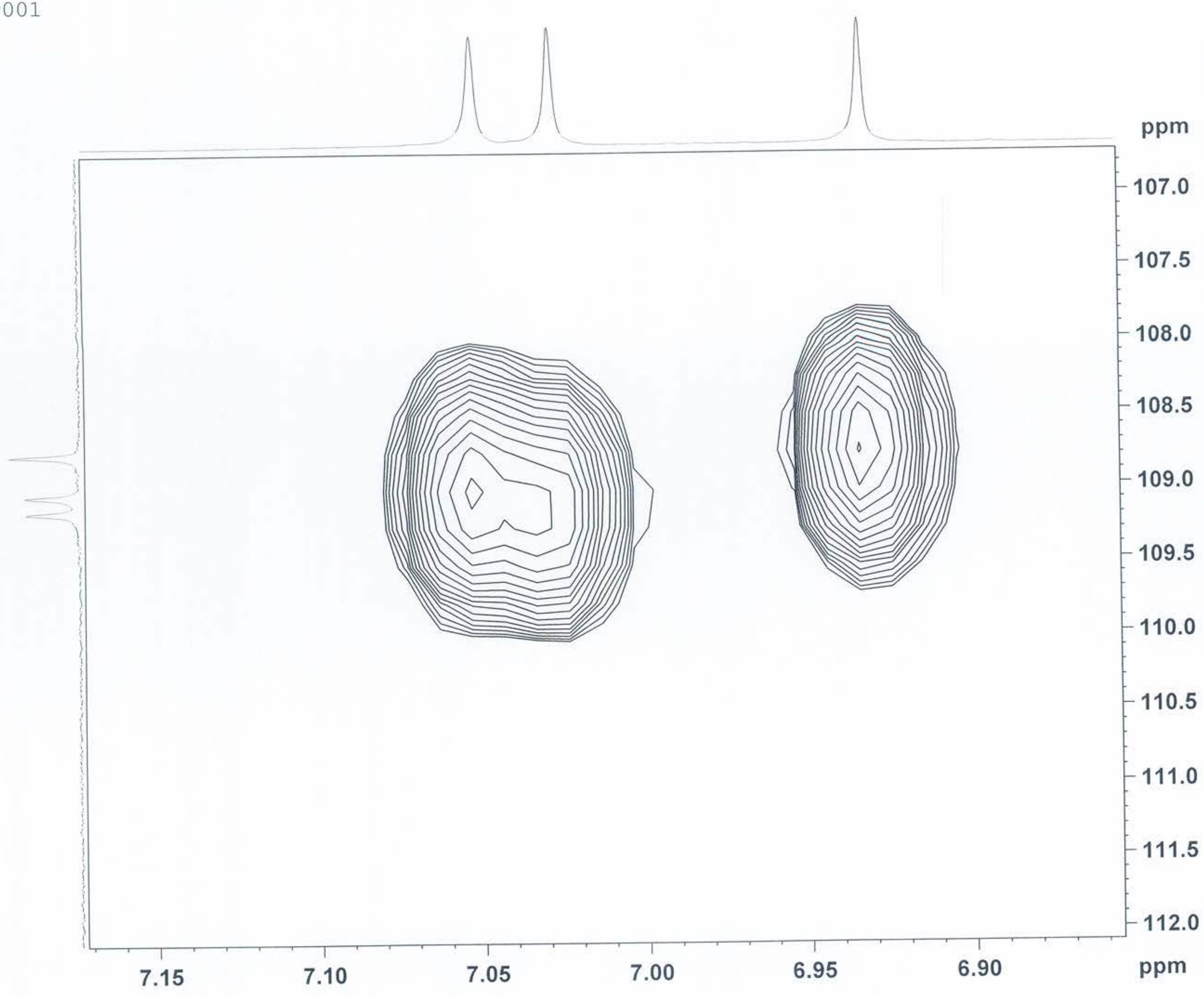

BRUKER

Current Data Parameters
NAME Component 5 of Chebulae Fructus_230401
EXPNO 6
PROCNO 1

F2 - Acquisition Parameters
Date_ 20231001
Time 16.17 h
INSTRUM Avance
PROBHD Z172446_0005 (
PULPROG hmbcetgpl3nd
TD 4096
SOLVENT DMSO
NS 64
DS 16
SWH 11904.762 Hz
FIDRES 5.812872 Hz
AQ 0.1720320 sec
RG 101
DW 42.000 usec
DE 6.50 usec
TE 298.2 K
CNST6 120.0000000
CNST7 170.0000000
CNST13 8.0000000
D0 0.00000300 sec
D1 2.00000000 sec
D6 0.06250000 sec
D16 0.00020000 sec
IN0 0.00001300 sec
TDav 1
SFO1 600.1336008 MHz
NUC1 1H
P1 11.92 usec
P2 23.84 usec
PLW1 17.17900085 W
SFO2 150.9178988 MHz
NUC2 13C
P3 11.80 usec
P24 2000.00 usec
PLW2 86.66300201 W
P16 1000.00 usec
CNST30 0.598116

F1 - Acquisition parameters
TD 256
SFO1 150.9179 MHz
FIDRES 300.625305 Hz
SW 254.973 ppm
FnMODE Echo-Antiecho

F2 - Processing parameters
SI 2048
SF 600.1300140 MHz
WDW SINE
SSB 4
LB 0 Hz
GB 0
PC 1.40

F1 - Processing parameters
SI 1024
MC2 echo-antiecho
SF 150.9028720 MHz
WDW QSINE
SSB 2
LB 0 Hz
GB 0

[illegible] 23.12.07
[illegible] 23.12.07

HP001

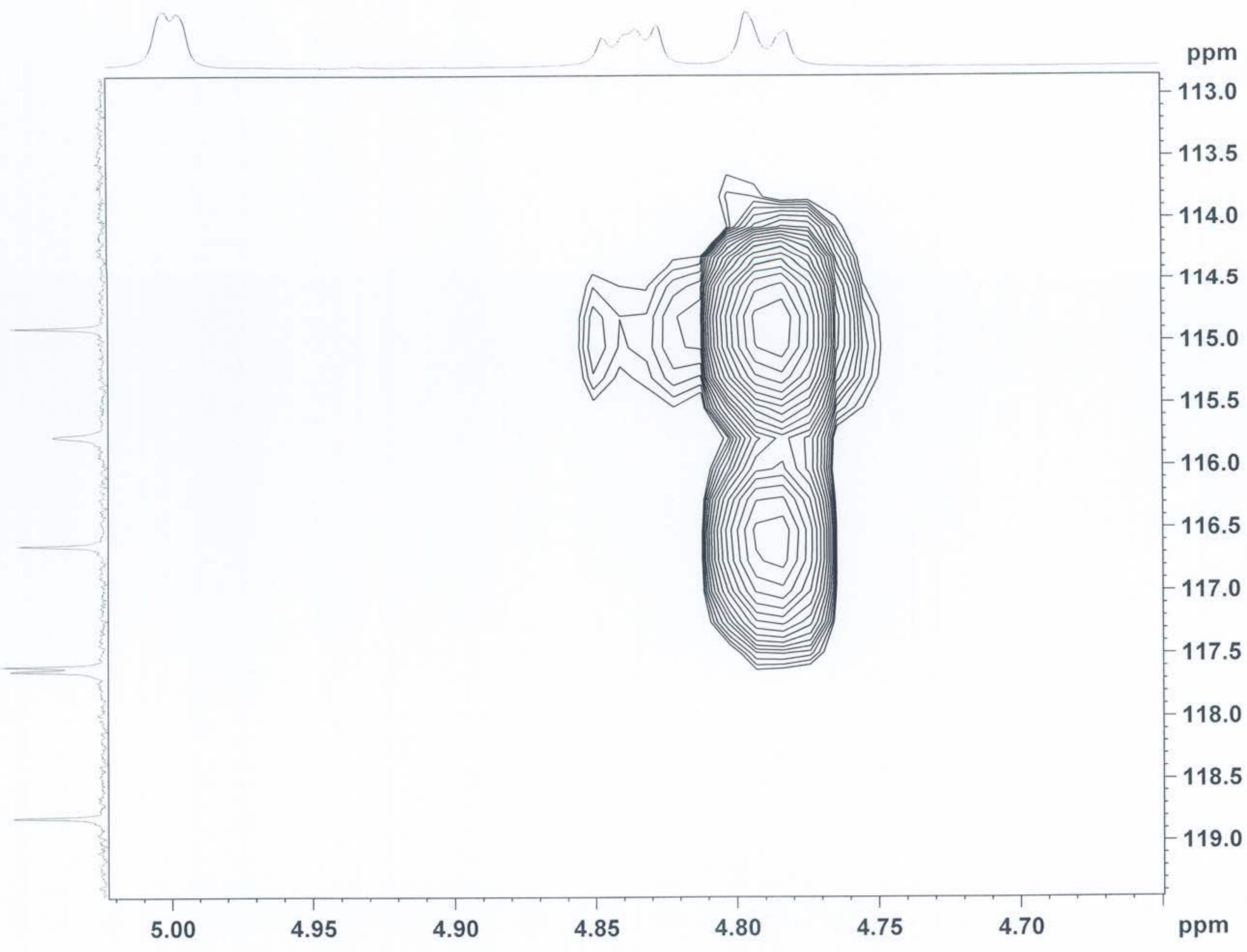

BRUKER

Current Data Parameters
NAME Component 5 of Chebulae Fructus_230401
EXPNO 6
PROCNO 1

F2 - Acquisition Parameters

Date_	20231001
Time	16.17 h
INSTRUM	Avance
PROBHD	Z172446_0005 (
PULPROG	hmbcetgpl3nd
TD	4096
SOLVENT	DMSO
NS	64
DS	16
SWH	11904.762 Hz
FIDRES	5.812872 Hz
AQ	0.1720320 sec
RG	101
DW	42.000 usec
DE	6.50 usec
TE	298.2 K
CNST6	120.0000000
CNST7	170.0000000
CNST13	8.0000000
D0	0.00000300 sec
D1	2.00000000 sec
D6	0.06250000 sec
D16	0.00020000 sec
IN0	0.00001300 sec
TDav	1
SFO1	600.1336008 MHz
NUC1	1H
P1	11.92 usec
P2	23.84 usec
PLW1	17.17900085 W
SFO2	150.9178988 MHz
NUC2	13C
P3	11.80 usec
P24	2000.00 usec
PLW2	86.66300201 W
P16	1000.00 usec
CNST30	0.598116

F1 - Acquisition parameters

TD	256
SFO1	150.9179 MHz
FIDRES	300.625305 Hz
SW	254.973 ppm
FnMODE	Echo-Antiecho

F2 - Processing parameters

SI	2048
SF	600.1300140 MHz
WDW	SINE
SSB	4
LB	0 Hz
GB	0
PC	1.40

F1 - Processing parameters

SI	1024
MC2	echo-antiecho
SF	150.9028720 MHz
WDW	QSINE
SSB	2
LB	0 Hz
GB	0

检测人：[illegible] 23.12.07
复核人：[illegible] 23.12.07

HP001

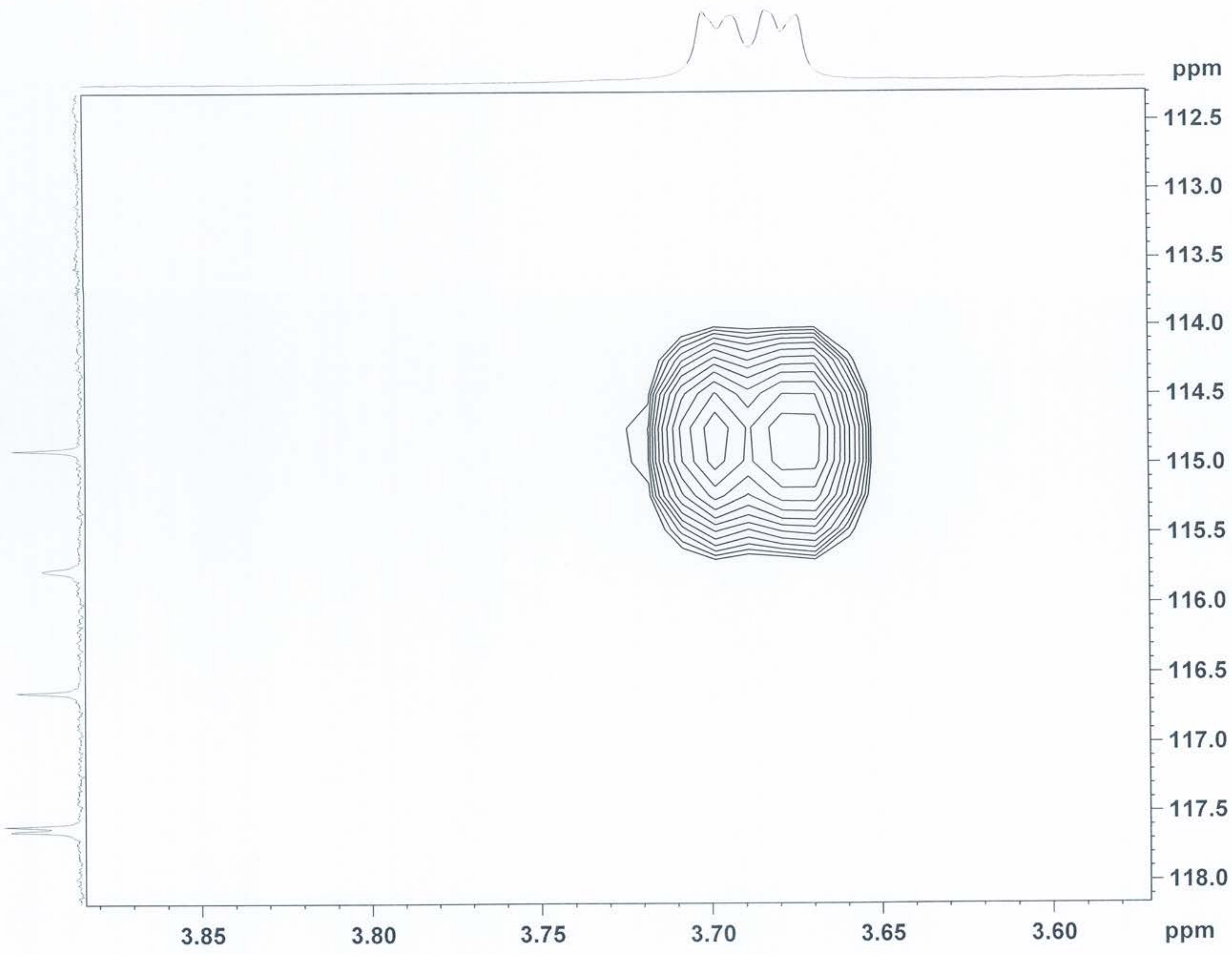

BRUKER

Current Data Parameters

NAME	Component 5 of Chebulae Fructus_230401
EXPNO	6
PROCNO	1

F2 - Acquisition Parameters

Date_	20231001
Time_	16.17 h
INSTRUM	Avance
PROBHD	Z172446_0005 (
PULPROG	hmbcetgpl3nd
TD	4096
SOLVENT	DMSO
NS	64
DS	16
SWH	11904.762 Hz
FIDRES	5.812872 Hz
AQ	0.1720320 sec
RG	101
DW	42.000 usec
DE	6.50 usec
TE	298.2 K
CNST6	120.0000000
CNST7	170.0000000
CNST13	8.0000000
D0	0.00000300 sec
D1	2.00000000 sec
D6	0.06250000 sec
D16	0.00020000 sec
IN0	0.00001300 sec
TDav	1
SFO1	600.1336008 MHz
NUC1	1H
P1	11.92 usec
P2	23.84 usec
PLW1	17.17900085 W
SFO2	150.9178988 MHz
NUC2	13C
P3	11.80 usec
P24	2000.00 usec
PLW2	86.66300201 W
P16	1000.00 usec
CNST30	0.598116

F1 - Acquisition parameters

TD	256
SFO1	150.9179 MHz
FIDRES	300.625305 Hz
SW	254.973 ppm
FnMODE	Echo-Antiecho

F2 - Processing parameters

SI	2048
SF	600.1300140 MHz
WDW	SINE
SSB	4
LB	0 Hz
GB	0
PC	1.40

F1 - Processing parameters

SI	1024
MC2	echo-antiecho
SF	150.9028720 MHz
WDW	QSINE
SSB	2
LB	0 Hz
GB	0

检测人：[illegible] 23.12.07
复核人：[illegible] 23.12.07

HP001

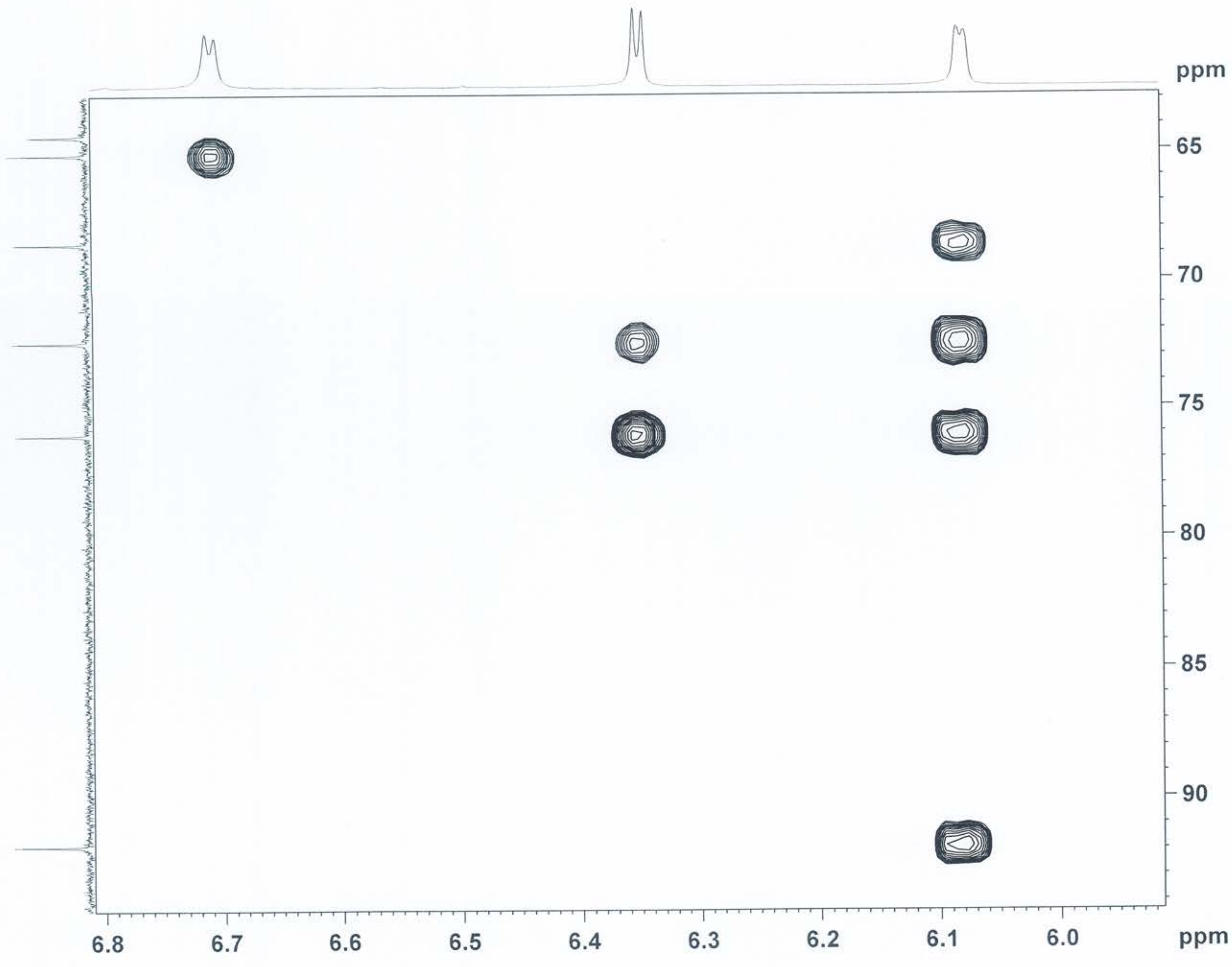

Current Data Parameters
NAME Component 5 of Chebulae Fructus_230401
EXPNO 6
PROCNO 1

F2 - Acquisition Parameters
Date_ 20231001
Time 16.17 h
INSTRUM Avance
PROBHD Z172446_0005 (
PULPROG hmbcetgpl3nd
TD 4096
SOLVENT DMSO
NS 64
DS 16
SWH 11904.762 Hz
FIDRES 5.812872 Hz
AQ 0.1720320 sec
RG 101
DW 42.000 usec
DE 6.50 usec
TE 298.2 K
CNST6 120.0000000
CNST7 170.0000000
CNST13 8.0000000
D0 0.00000300 sec
D1 2.00000000 sec
D6 0.06250000 sec
D16 0.00020000 sec
IN0 0.00001300 sec
TDav 1
SFO1 600.1336008 MHz
NUC1 1H
P1 11.92 usec
P2 23.84 usec
PLW1 17.17900085 W
SFO2 150.9178988 MHz
NUC2 13C
P3 11.80 usec
P24 2000.00 usec
PLW2 86.66300201 W
P16 1000.00 usec
CNST30 0.598116

F1 - Acquisition parameters
TD 256
SFO1 150.9179 MHz
FIDRES 300.625305 Hz
SW 254.973 ppm
FnMODE Echo-Antiecho

F2 - Processing parameters
SI 2048
SF 600.1300140 MHz
WDW SINE
SSB 4
LB 0 Hz
GB 0
PC 1.40

F1 - Processing parameters
SI 1024
MC2 echo-antiecho
SF 150.9028720 MHz
WDW QSINE
SSB 2
LB 0 Hz
GB 0

[illegible] 23.12.07
[illegible] 23.12.07

HP001

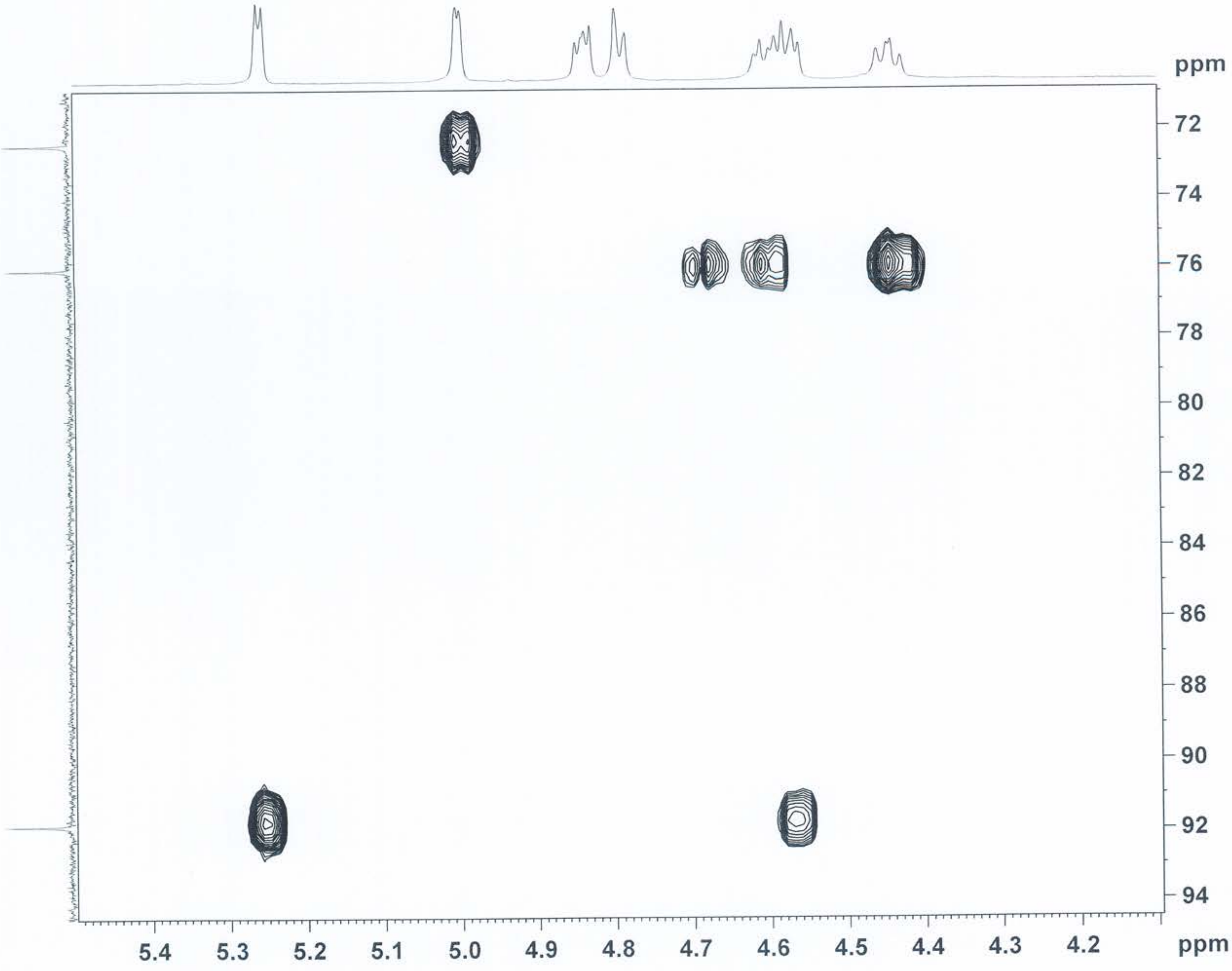

BRUKER

Current Data Parameters

NAME	Component 5 of Chebulae Fructus_230401	
EXPNO	6	
PROCNO	1	

F2 - Acquisition Parameters

Date_	20231001	
Time	16.17	h
INSTRUM	Avance	
PROBHD	Z172446_0005 (	
PULPROG	hmbcetgpl3nd	
TD	4096	
SOLVENT	DMSO	
NS	64	
DS	16	
SWH	11904.762	Hz
FIDRES	5.812872	Hz
AQ	0.1720320	sec
RG	101	
DW	42.000	usec
DE	6.50	usec
TE	298.2	K
CNST6	120.0000000	
CNST7	170.0000000	
CNST13	8.0000000	
D0	0.00000300	sec
D1	2.00000000	sec
D6	0.06250000	sec
D16	0.00020000	sec
IN0	0.00001300	sec
TDav	1	
SFO1	600.1336008	MHz
NUC1	1H	
P1	11.92	usec
P2	23.84	usec
PLW1	17.17900085	W
SFO2	150.9178988	MHz
NUC2	13C	
P3	11.80	usec
P24	2000.00	usec
PLW2	86.66300201	W
P16	1000.00	usec
CNST30	0.598116	

F1 - Acquisition parameters

TD	256	
SFO1	150.9179	MHz
FIDRES	300.625305	Hz
SW	254.973	ppm
FnMODE	Echo-Antiecho	

F2 - Processing parameters

SI	2048	
SF	600.1300140	MHz
WDW	SINE	
SSB	4	
LB	0	Hz
GB	0	
PC	1.40	

F1 - Processing parameters

SI	1024	
MC2	echo-antiecho	
SF	150.9028720	MHz
WDW	QSINE	
SSB	2	
LB	0	Hz
GB	0	

检测人：[illegible] 23.12.07
复核人：[illegible] 23.12.07

HP001

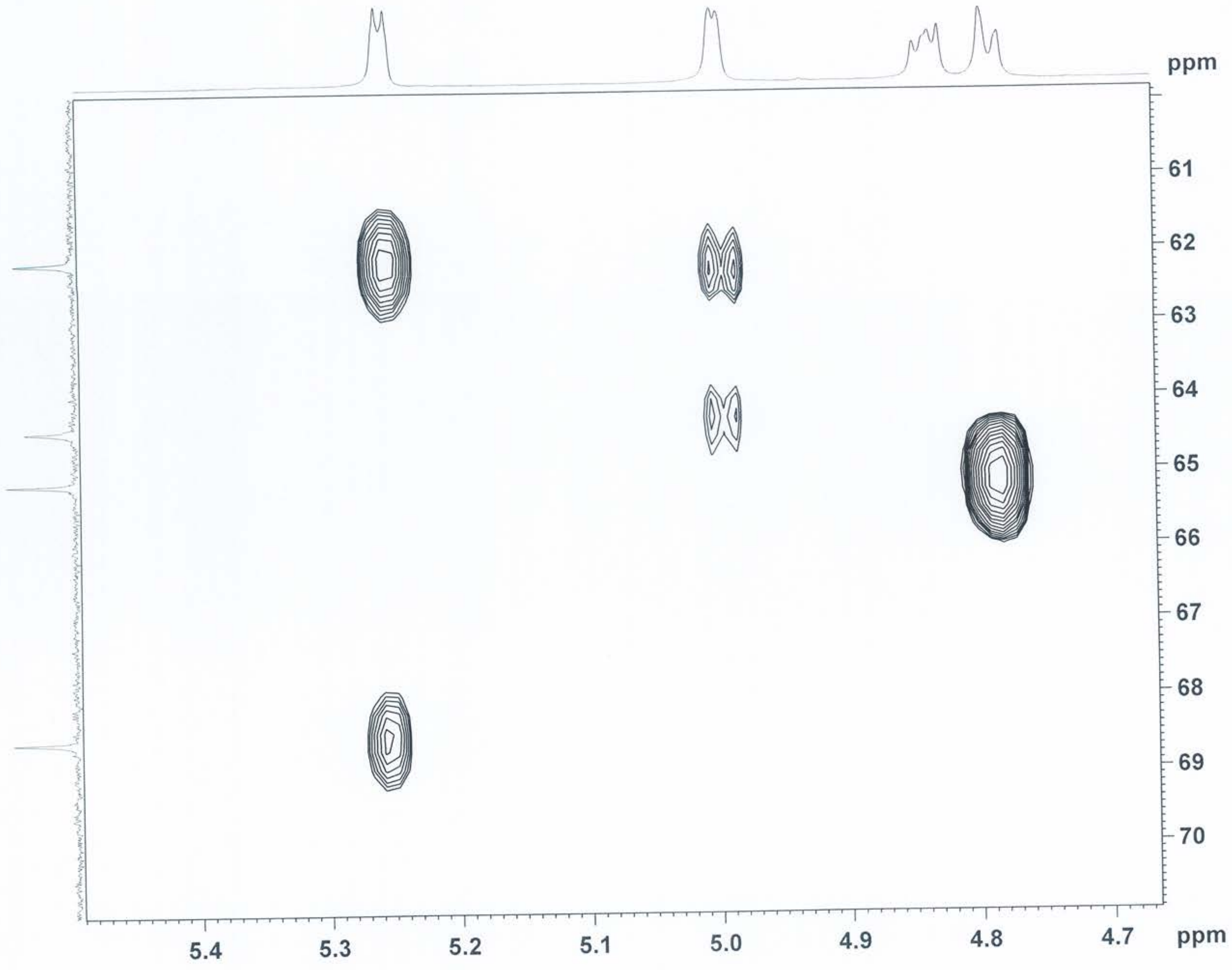

BRUKER

```
Current Data Parameters
NAME      Component 5 of
          Chebulae Fructus_230401
EXPNO                  6
PROCNO                 1

F2 - Acquisition Parameters
Date_            20231001
Time              16.17 h
INSTRUM            Avance
PROBHD     Z172446_0005 (
PULPROG    hmbcetgpl3nd
TD                   4096
SOLVENT              DMSO
NS                     64
DS                     16
SWH            11904.762 Hz
FIDRES          5.812872 Hz
AQ             0.1720320 sec
RG                    101
DW                 42.000 usec
DE                   6.50 usec
TE                  298.2 K
CNST6         120.0000000
CNST7         170.0000000
CNST13          8.0000000
D0             0.00000300 sec
D1             2.00000000 sec
D6             0.06250000 sec
D16            0.00020000 sec
IN0            0.00001300 sec
TDav                    1
SFO1          600.1336008 MHz
NUC1                   1H
P1                  11.92 usec
P2                  23.84 usec
PLW1          17.17900085 W
SFO2          150.9178988 MHz
NUC2                  13C
P3                  11.80 usec
P24               2000.00 usec
PLW2          86.66300201 W
P16               1000.00 usec
CNST30           0.598116

F1 - Acquisition parameters
TD                    256
SFO1             150.9179 MHz
FIDRES         300.625305 Hz
SW                254.973 ppm
FnMODE       Echo-Antiecho

F2 - Processing parameters
SI                   2048
SF            600.1300140 MHz
WDW                  SINE
SSB                     4
LB                   0 Hz
GB                      0
PC                   1.40

F1 - Processing parameters
SI                   1024
MC2           echo-antiecho
SF            150.9028720 MHz
WDW                 QSINE
SSB                     2
LB                   0 Hz
GB                      0
```

检测人：[illegible] 23.12.07

复核人：[illegible] 23.12.07

HP001

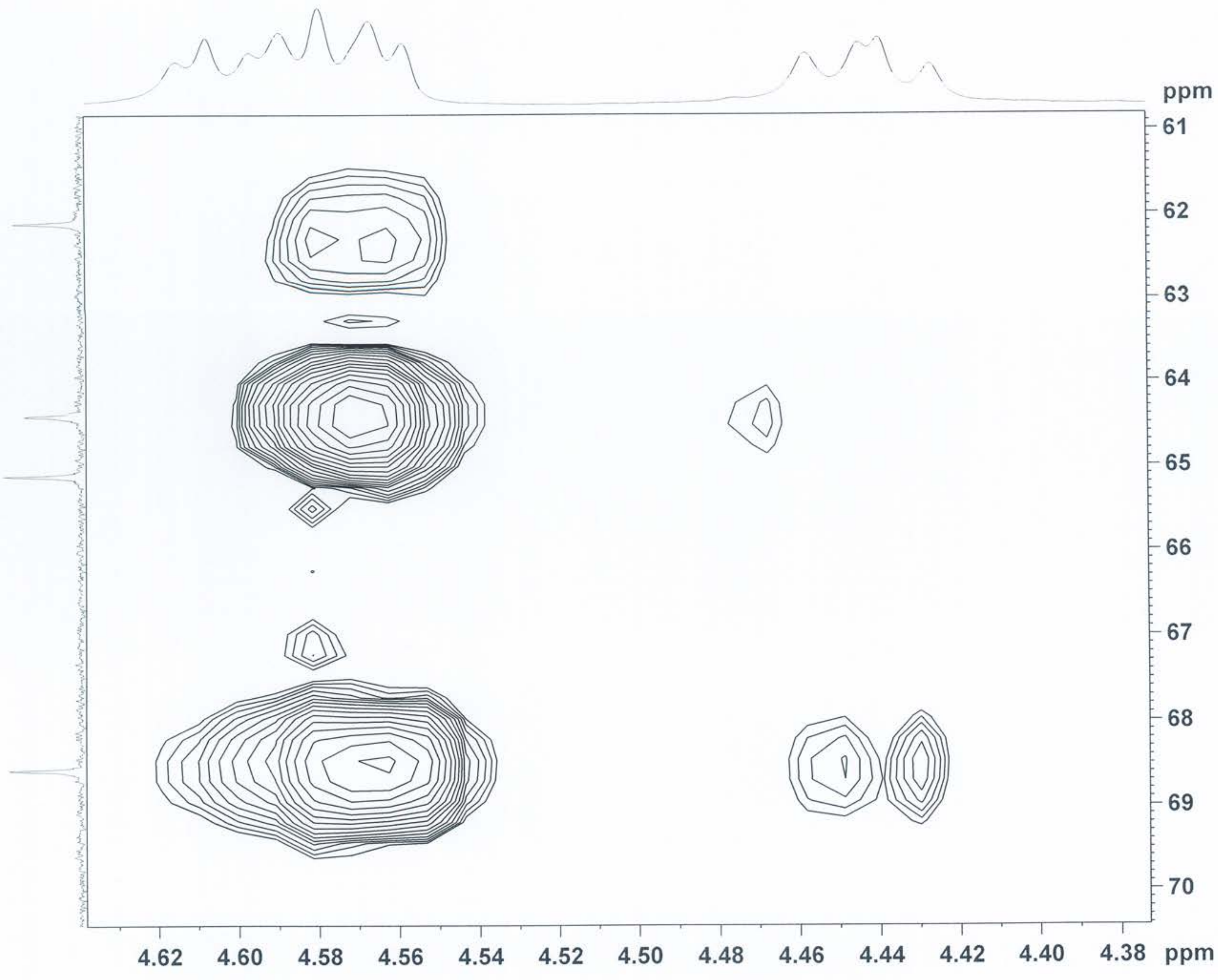

Current Data Parameters
NAME Component 5 of Chebulae Fructus_230401
EXPNO 6
PROCNO 1

F2 - Acquisition Parameters
Date_ 20231001
Time_ 16.17 h
INSTRUM Avance
PROBHD Z172446_0005 (
PULPROG hmbcetgpl3nd
TD 4096
SOLVENT DMSO
NS 64
DS 16
SWH 11904.762 Hz
FIDRES 5.812872 Hz
AQ 0.1720320 sec
RG 101
DW 42.000 usec
DE 6.50 usec
TE 298.2 K
CNST6 120.0000000
CNST7 170.0000000
CNST13 8.0000000
D0 0.00000300 sec
D1 2.00000000 sec
D6 0.06250000 sec
D16 0.00020000 sec
IN0 0.00001300 sec
TDav 1
SFO1 600.1336008 MHz
NUC1 1H
P1 11.92 usec
P2 23.84 usec
PLW1 17.17900085 W
SFO2 150.9178988 MHz
NUC2 13C
P3 11.80 usec
P24 2000.00 usec
PLW2 86.66300201 W
P16 1000.00 usec
CNST30 0.598116

F1 - Acquisition parameters
TD 256
SFO1 150.9179 MHz
FIDRES 300.625305 Hz
SW 254.973 ppm
FnMODE Echo-Antiecho

F2 - Processing parameters
SI 2048
SF 600.1300140 MHz
WDW SINE
SSB 4
LB 0 Hz
GB 0
PC 1.40

F1 - Processing parameters
SI 1024
MC2 echo-antiecho
SF 150.9028720 MHz
WDW QSINE
SSB 2
LB 0 Hz
GB 0

核对人：[illegible] 23.12.07
复核人：[illegible] 23.12.07

HP001

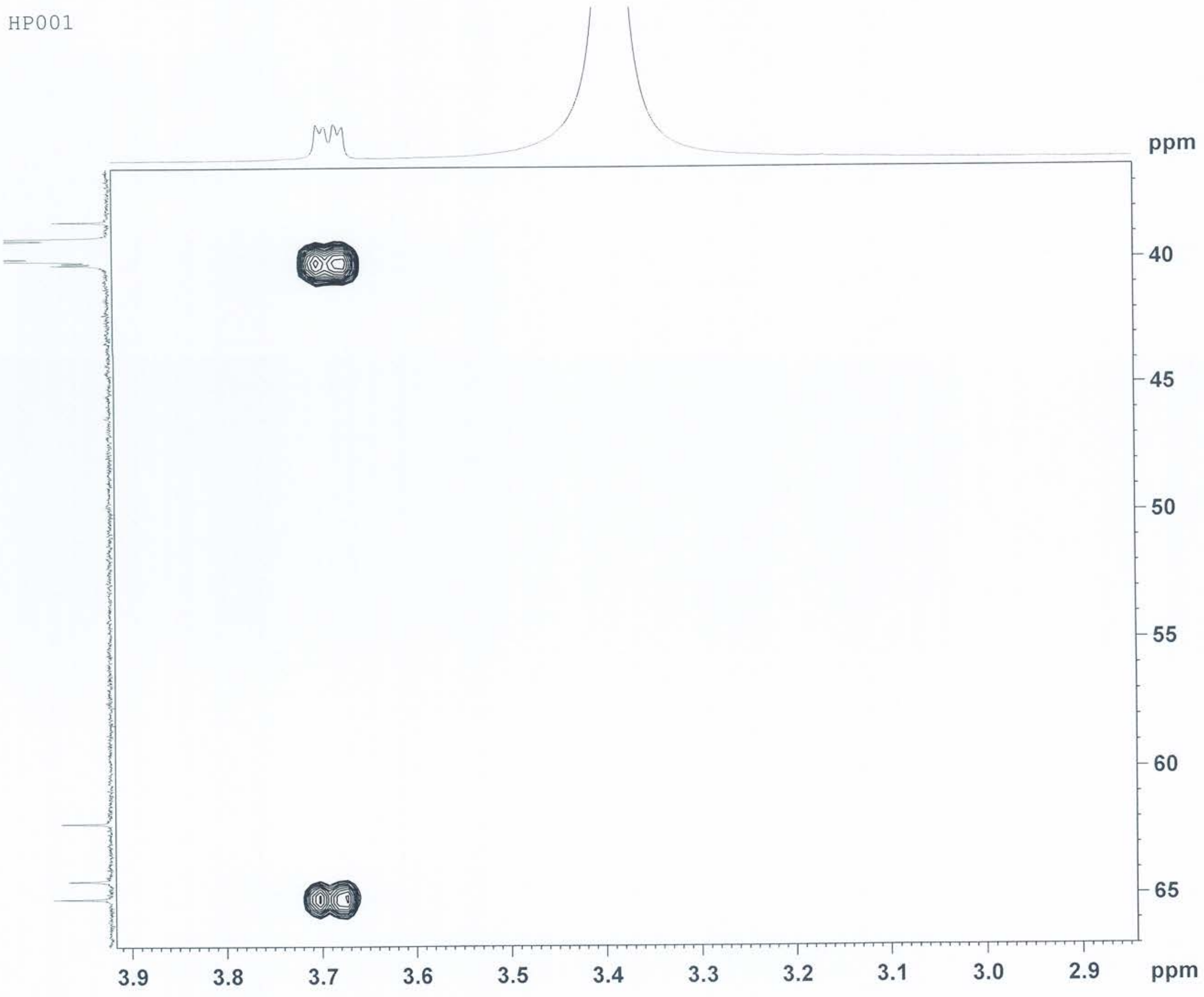

BRUKER

```
Current Data Parameters
NAME        Component 5 of
            Chebulae Fructus_230401
EXPNO                    6
PROCNO                   1

F2 - Acquisition Parameters
Date_             20231001
Time                 16.17 h
INSTRUM             Avance
PROBHD    Z172446_0005 (
PULPROG      hmbcetgpl3nd
TD                    4096
SOLVENT               DMSO
NS                      64
DS                      16
SWH             11904.762 Hz
FIDRES           5.812872 Hz
AQ              0.1720320 sec
RG                     101
DW                  42.000 usec
DE                    6.50 usec
TE                   298.2 K
CNST6         120.0000000
CNST7         170.0000000
CNST13          8.0000000
D0             0.00000300 sec
D1             2.00000000 sec
D6             0.06250000 sec
D16            0.00020000 sec
IN0            0.00001300 sec
TDav                     1
SFO1          600.1336008 MHz
NUC1                    1H
P1                   11.92 usec
P2                   23.84 usec
PLW1          17.17900085 W
SFO2          150.9178988 MHz
NUC2                   13C
P3                   11.80 usec
P24                2000.00 usec
PLW2          86.66300201 W
P16                1000.00 usec
CNST30            0.598116

F1 - Acquisition parameters
TD                     256
SFO1              150.9179 MHz
FIDRES          300.625305 Hz
SW                 254.973 ppm
FnMODE        Echo-Antiecho

F2 - Processing parameters
SI                    2048
SF             600.1300140 MHz
WDW                   SINE
SSB                      4
LB                  0 Hz
GB                  0
PC                    1.40

F1 - Processing parameters
SI                    1024
MC2           echo-antiecho
SF             150.9028720 MHz
WDW                  QSINE
SSB                      2
LB                  0 Hz
GB                  0
```

[illegible]人：[illegible] 23.12.07

复核人：[illegible] 23.12.07

HP001

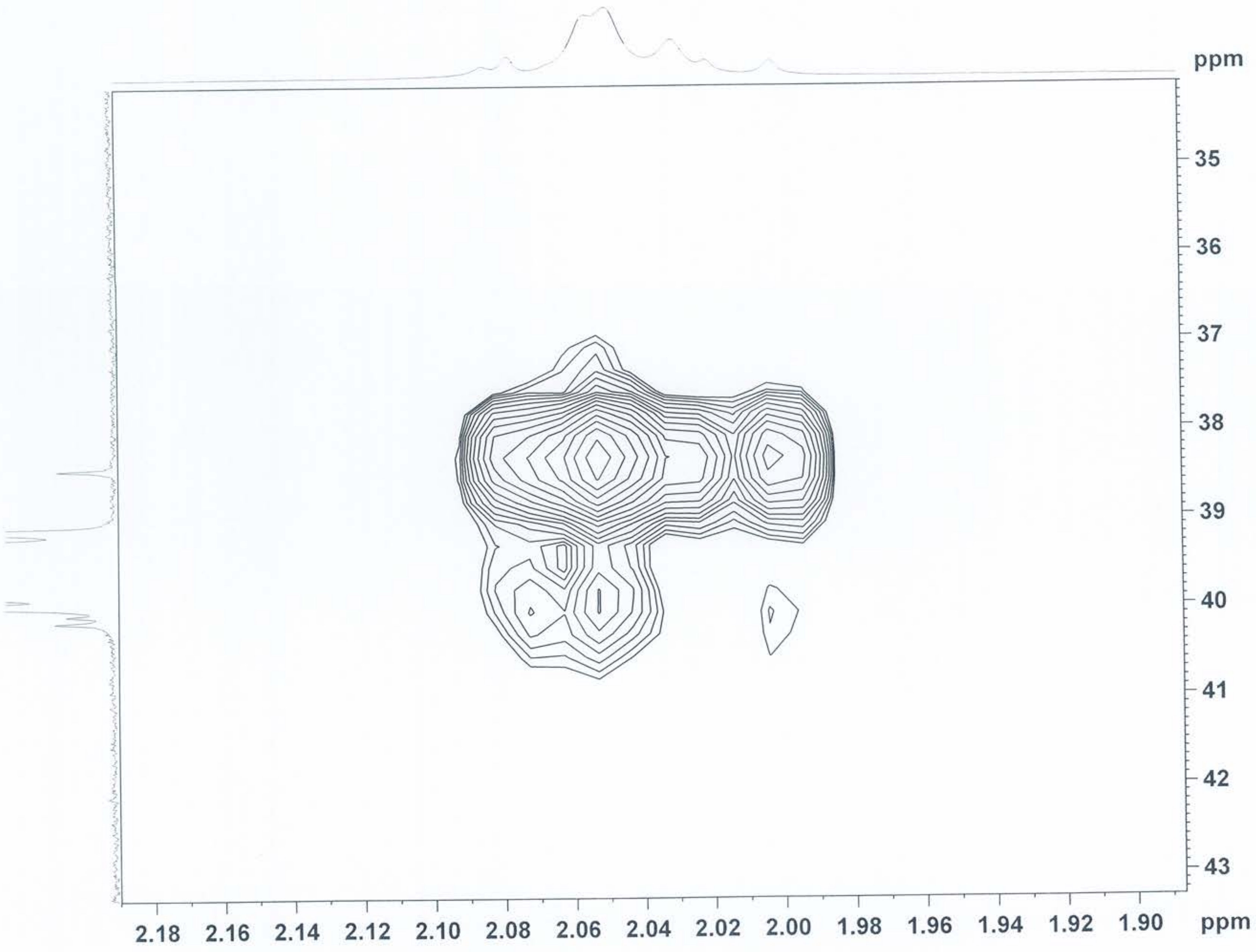

BRUKER

Current Data Parameters
NAME Component 5 of Chebulae Fructus_230401
EXPNO 6
PROCNO 1

F2 - Acquisition Parameters
Date_ 20231001
Time 16.17 h
INSTRUM Avance
PROBHD Z172446_0005 (
PULPROG hmbcetgpl3nd
TD 4096
SOLVENT DMSO
NS 64
DS 16
SWH 11904.762 Hz
FIDRES 5.812872 Hz
AQ 0.1720320 sec
RG 101
DW 42.000 usec
DE 6.50 usec
TE 298.2 K
CNST6 120.0000000
CNST7 170.0000000
CNST13 8.0000000
D0 0.00000300 sec
D1 2.00000000 sec
D6 0.06250000 sec
D16 0.00020000 sec
IN0 0.00001300 sec
TDav 1
SFO1 600.1336008 MHz
NUC1 1H
P1 11.92 usec
P2 23.84 usec
PLW1 17.17900085 W
SFO2 150.9178988 MHz
NUC2 13C
P3 11.80 usec
P24 2000.00 usec
PLW2 86.66300201 W
P16 1000.00 usec
CNST30 0.598116

F1 - Acquisition parameters
TD 256
SFO1 150.9179 MHz
FIDRES 300.625305 Hz
SW 254.973 ppm
FnMODE Echo-Antiecho

F2 - Processing parameters
SI 2048
SF 600.1300140 MHz
WDW SINE
SSB 4
LB 0 Hz
GB 0
PC 1.40

F1 - Processing parameters
SI 1024
MC2 echo-antiecho
SF 150.9028720 MHz
WDW QSINE
SSB 2
LB 0 Hz
GB 0

检测人：[illegible] 23.12.07
审核人：[illegible] 23.12.07

HP001

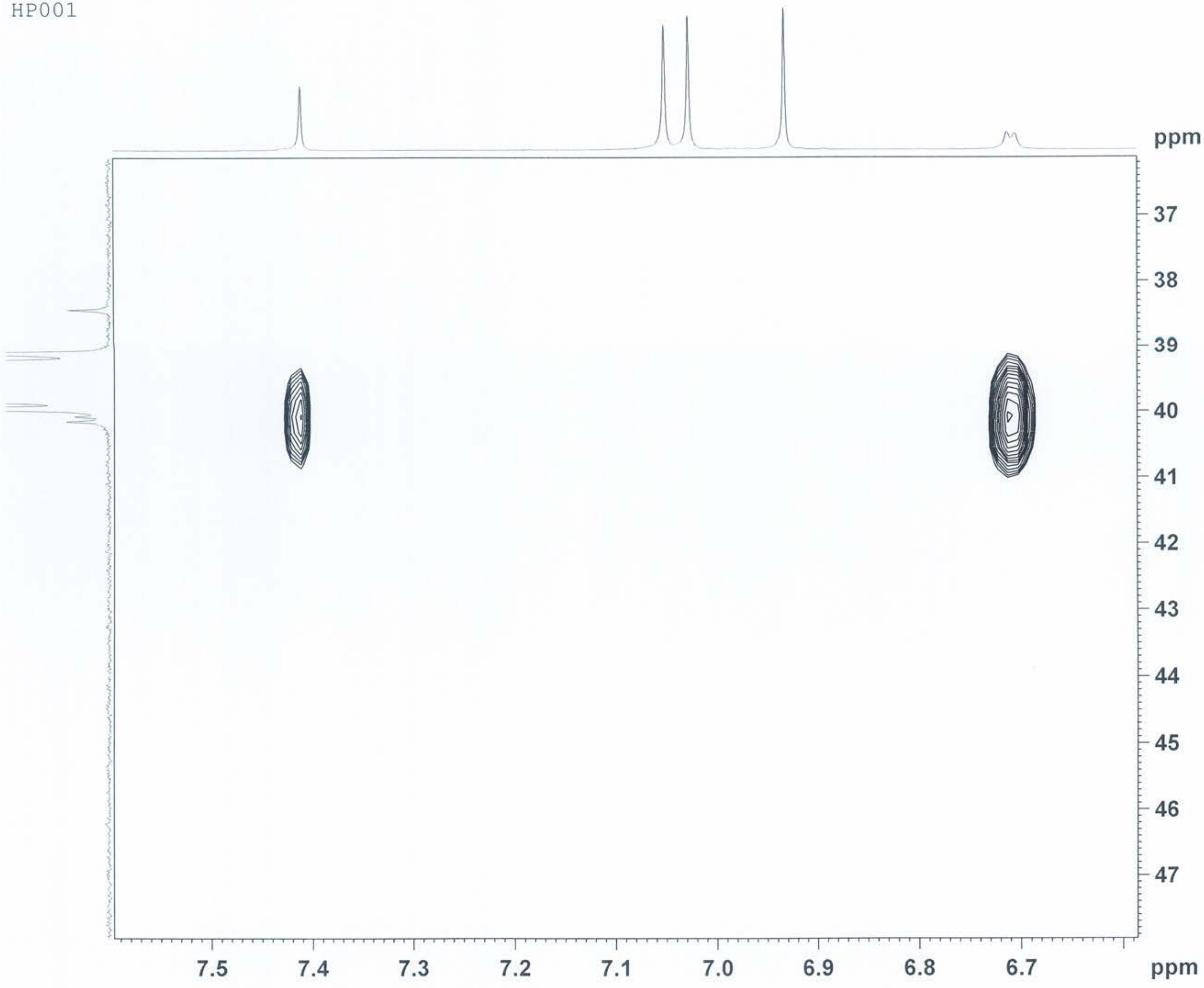

BRUKER

Current Data Parameters	
NAME	Component 5 of Chebulae Fructus_230401
EXPNO	6
PROCNO	1
F2 - Acquisition Parameters	
Date_	20231001
Time	16.17 h
INSTRUM	Avance
PROBHD	Z172446_0005 (
PULPROG	hmbcetgpl3nd
TD	4096
SOLVENT	DMSO
NS	64
DS	16
SWH	11904.762 Hz
FIDRES	5.812872 Hz
AQ	0.1720320 sec
RG	101
DW	42.000 usec
DE	6.50 usec
TE	298.2 K
CNST6	120.0000000
CNST7	170.0000000
CNST13	8.0000000
D0	0.00000300 sec
D1	2.00000000 sec
D6	0.06250000 sec
D16	0.00020000 sec
IN0	0.00001300 sec
TDav	1
SFO1	600.1336008 MHz
NUC1	1H
P1	11.92 usec
P2	23.84 usec
PLW1	17.17900085 W
SFO2	150.9178988 MHz
NUC2	13C
P3	11.80 usec
P24	2000.00 usec
PLW2	86.66300201 W
P16	1000.00 usec
CNST30	0.598116
F1 - Acquisition parameters	
TD	256
SFO1	150.9179 MHz
FIDRES	300.625305 Hz
SW	254.973 ppm
FnMODE	Echo-Antiecho
F2 - Processing parameters	
SI	2048
SF	600.1300140 MHz
WDW	SINE
SSB	4
LB	0 Hz
GB	0
PC	1.40
F1 - Processing parameters	
SI	1024
MC2	echo-antiecho
SF	150.9028720 MHz
WDW	QSINE
SSB	2
LB	0 Hz
GB	0

[illegible] 23.12.07
[illegible] 23.12.07

HP001

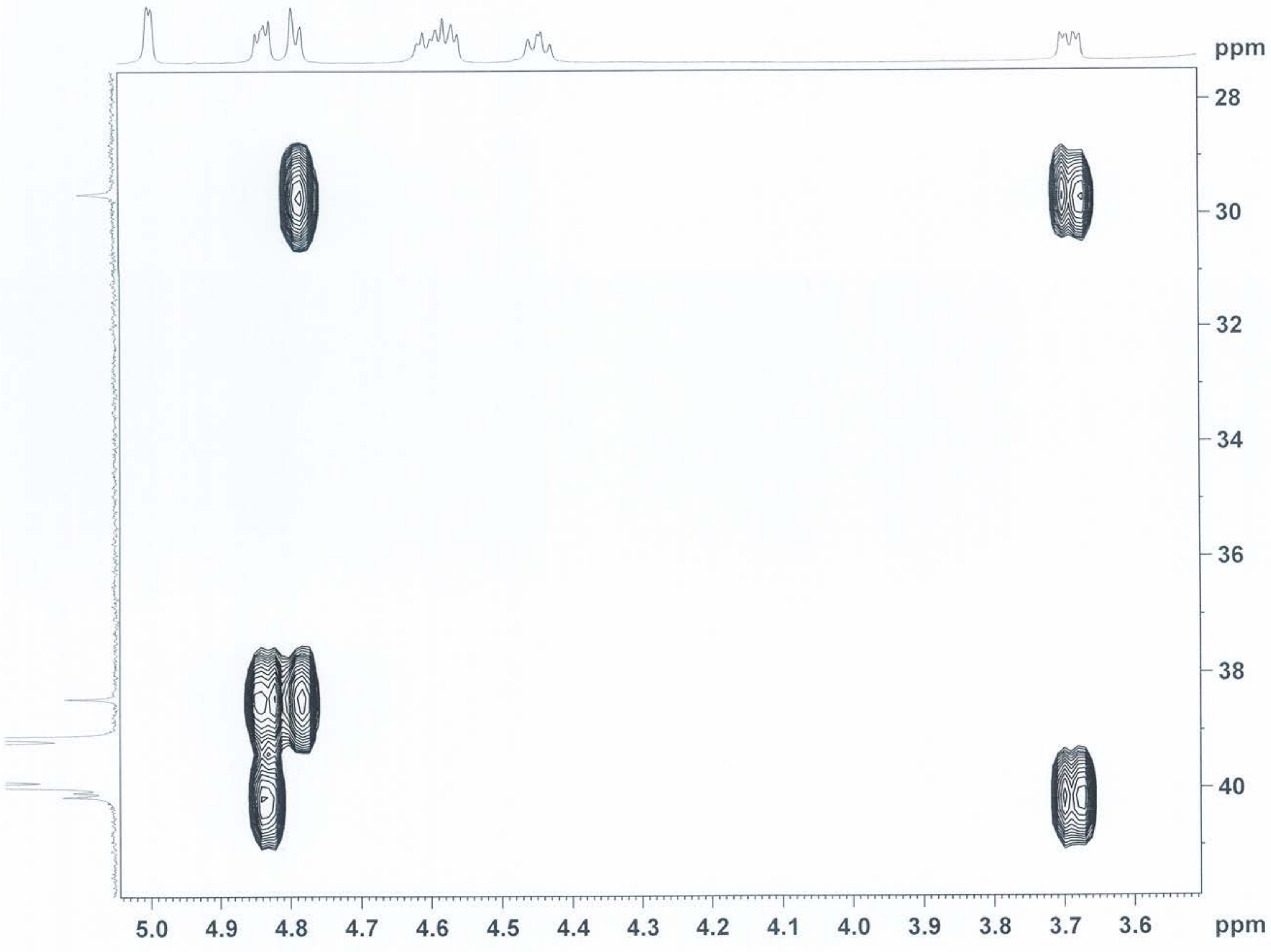

BRUKER

Current Data Parameters

NAME	Component 5 of Chebulae Fructus_230401	
EXPNO	6	
PROCNO	1	

F2 - Acquisition Parameters

Date_	20231001	
Time_	16.17	h
INSTRUM	Avance	
PROBHD	2172446_0005 (	
PULPROG	hmbcetgpl3nd	
TD	4096	
SOLVENT	DMSO	
NS	64	
DS	16	
SWH	11904.762	Hz
FIDRES	5.812872	Hz
AQ	0.1720320	sec
RG	101	
DW	42.000	usec
DE	6.50	usec
TE	298.2	K
CNST6	120.0000000	
CNST7	170.0000000	
CNST13	8.0000000	
D0	0.00000300	sec
D1	2.00000000	sec
D6	0.06250000	sec
D16	0.00020000	sec
IN0	0.00001300	sec
TDav	1	
SFO1	600.1336008	MHz
NUC1	1H	
P1	11.92	usec
P2	23.84	usec
PLW1	17.17900085	W
SFO2	150.9178988	MHz
NUC2	13C	
P3	11.80	usec
P24	2000.00	usec
PLW2	86.66300201	W
P16	1000.00	usec
CNST30	0.598116	

F1 - Acquisition parameters

TD	256	
SFO1	150.9179	MHz
FIDRES	300.625305	Hz
SW	254.973	ppm
FnMODE	Echo-Antiecho	

F2 - Processing parameters

SI	2048	
SF	600.1300140	MHz
WDW	SINE	
SSB	4	
LB	0	Hz
GB	0	
PC	1.40	

F1 - Processing parameters

SI	1024	
MC2	echo-antiecho	
SF	150.9028720	MHz
WDW	QSINE	
SSB	2	
LB	0	Hz
GB	0	

[illegible] 23.12.07

复核人[illegible] 23.12.07
